# General Relativity and the AKSZ Construction

**DOI:** 10.1007/s00220-021-04127-6

**Published:** 2021-07-02

**Authors:** G. Canepa, A. S. Cattaneo, M. Schiavina

**Affiliations:** 1 Institut für Mathematik, Universität Zürich, Winterthurerstrasse 190, 8057 Zurich, Switzerland; 2 Institute for Theoretical Physics, ETH Zurich, Wolfgang Pauli strasse 27, 8092 Zurich, Switzerland; 3 Department of Mathematics, ETH Zurich, Rämistrasse 101, 8092 Zurich, Switzerland

## Abstract

In this note the AKSZ construction is applied to the BFV description of the reduced phase space of the Einstein–Hilbert and of the Palatini–Cartan theories in every space-time dimension greater than two. In the former case one obtains a BV theory for the first-order formulation of Einstein–Hilbert theory, in the latter a BV theory for Palatini–Cartan theory with a partial implementation of the torsion-free condition already on the space of fields. All theories described here are BV versions of the same classical system on cylinders. The AKSZ implementations we present have the advantage of yielding a compatible BV–BFV description, which is the required starting point for a quantization in presence of a boundary.

## Introduction

A Lagrangian field theory ${\mathsf {F}}$ on a cylinder $\Sigma \times I$, where *I* is a “time” interval, can be given a corresponding Hamiltonian description in terms of a symplectic manifold (the phase space) of the possible initial conditions on $\Sigma$ and a Hamiltonian that describes the time evolution. If the Lagrangian is degenerate, its Euler–Lagrange equations yield, in addition to time evolution, some constraints that have to be taken into account when specifying the initial conditions. The true phase space, called the “reduced phase space”, is typically described as the symplectic reduction of the coisotropic submanifold defined by the constraints (hence the name).

This reduction is often singular, and one possible description is by means of a cohomological resolution: one introduces a complex whose cohomology is the algebra of functions of the reduced phase space. In addition, one wants this resolution to feature also the symplectic/Poisson nature of the phase space, and a solution to this problem is provided by the Batalin–Fradkin–Vilkovisky (BFV) formalism [BF83] (see also [Sch08, Sch09, Sta97]). We denote by ${\mathfrak {F}}^\partial$ the collection of data associated to the reduced phase space of a Lagrangian theory ${\mathsf {F}}$, as a BFV theory (Definition 3).

On the other hand, a flexible way to deal with a degenerate Lagrangian is the Batalin–Vilkovisky (BV) formalism [BV81], which allows a cohomological resolution of the space of solutions to the Euler–Lagrange equations modulo symmetries but is also the starting point for perturbative quantization. We denote with ${\mathfrak {F}}$ the BV data associated to a classical Lagrangian field theory ${\mathsf {F}}$, as a BV theory (Definition 1).

To quantize a Lagrangian field theory ${\mathsf {F}}$ on a cylinder $\Sigma \times I$, one needs a good relation between its associated BV and BFV data ${\mathfrak {F}}$ and ${\mathfrak {F}}^\partial$. In [CMR11] an explicit procedure was introduced to construct what in [CMR14] is called a BV–BFV theory (Definition 5), associating to the BV data ${\mathfrak {F}}$ certain BFV data denoted by ${{\,\mathrm{\textit{BFV}}\,}}({\mathfrak {F}})$ in a way suitable for quantization [CMR18]—under some regularity assumptions. In regular cases it relates ${\mathfrak {F}}$ and ${\mathfrak {F}}^\partial$, so that ${{\,\mathrm{\textit{BFV}}\,}}({\mathfrak {F}})={\mathfrak {F}}^\partial$.

While it is true that both ${\mathfrak {F}}$ and ${\mathfrak {F}}^\partial$ depend on ${\mathsf {F}}$, the relation ${{\,\mathrm{\textit{BFV}}\,}}({\mathfrak {F}})={\mathfrak {F}}^\partial$ is not guaranteed, and it is a necessary requirement for BV quantisation with boundary [CMR18]. This relation turns out to hold for a large variety of field theories, including general relativity (GR) in the Einstein–Hilbert (EH) formulation in any space-time dimension greater than 2 [CS16]. However, the procedure notably fails in the case of GR in the Palatini–Cartan (PC) formulation^1^ in $3+1$ dimensions [CS19b], as the construction of $BFV({\mathfrak {F}})$ is obstructed. However, ${\mathfrak {F}}^\partial$ exists and has been presented in [CCS20].

Conversely, given a BFV theory ${\mathfrak {F}}^\partial$ associated to a manifold $\Sigma$, there is a standard way^2^ to produce a BV theory on $\Sigma \times I$ by means of a construction due to Alexandrov, Kontsevich, Schwarz and Zaboronski (AKSZ [Ale+97]). The resulting BV theory, which we temporarily denote here by^3^${{\,\mathrm{\textit{AKSZ}}\,}}({\mathfrak {F}}^\partial )$, satisfies automatically the regularity assumptions required by the BV–BFV formalism, and we also have ${{\,\mathrm{\textit{BFV}}\,}}({{\,\mathrm{\textit{AKSZ}}\,}}({\mathfrak {F}}^\partial ))={\mathfrak {F}}^\partial$.

On the other hand, in general ${{\,\mathrm{\textit{AKSZ}}\,}}({{\,\mathrm{\textit{BFV}}\,}}({\mathfrak {F}}))$ will not be the same as ${\mathfrak {F}}$. In fact, the AKSZ construction produces a theory that is invariant under reparametrization of *I*, which is certainly different from ${\mathfrak {F}}$ if the latter does not enjoy this invariance. In this case ${{\,\mathrm{\textit{AKSZ}}\,}}({{\,\mathrm{\textit{BFV}}\,}}({\mathfrak {F}}))$ is a version of ${\mathfrak {F}}$ with “frozen time” and may be used to describe a change in the polarization chosen for the quantization of the reduced phase space (see [CMR18, Remark 2.38]). If ${\mathfrak {F}}$ is reparametrization invariant—e.g. a topological field theory or GR—we may wonder whether ${{\,\mathrm{\textit{AKSZ}}\,}}({{\,\mathrm{\textit{BFV}}\,}}({\mathfrak {F}}))$ and ${\mathfrak {F}}$ are somehow related. In the case of AKSZ topological field theories, it turns out that ${{\,\mathrm{\textit{AKSZ}}\,}}({{\,\mathrm{\textit{BFV}}\,}}({\mathfrak {F}}))$ and ${\mathfrak {F}}$ are actually the same. For more general reparametrization invariant theories we might expect the two to be equivalent, in one of the possible ways presented below.

A BV theory ${\mathfrak {F}}$ is essentially composed of a (− 1)-symplectic manifold $({\mathcal {F}},\varpi )$ and an action functional *S* over it. We say that ${\mathfrak {F}}_1$ and ${\mathfrak {F}}_2$ are strongly BV-equivalent if there is a symplectomorphism $\phi {:}\,({\mathcal {F}}_1,\varpi _1)\rightarrow ({\mathcal {F}}_2,\varpi _2)$ that relates their action functionals, i.e. $S_1=\phi ^*S_2$. This in particular implies that their BV cohomology groups are isomorphic. A nontrivial example of strong BV-equivalence is the one between PC and *BF* theory in 3 space-time dimensions [CS19a, CSS18].

If ${\mathfrak {F}}_2$ is obtained from ${\mathfrak {F}}_1$ by a partial integration of the fields^4^ (with some partial gauge fixing), we say that ${\mathfrak {F}}_2$ is an effective theory for ${\mathfrak {F}}_1$. We say that two BV theories are effectively BV-equivalent if one is (strongly BV-equivalent to) an effective theory for the other. Typical cases for this are Wilson renormalization or the passage to a second-order theory from its associated first-order formulation. Another important example is given by elimination of so-called auxiliary fields. In that case, one can argue that effective equivalence also preserves the BV cohomology [BBH95, Hen90] (see Remark 12).

A third case is when the theories ${\mathfrak {F}}_1$ and ${\mathfrak {F}}_2$ have the same space of classical solutions modulo symmetries. We speak in this case of classical equivalence. A typical case of classical equivalence is that between EH and PC. Observe that this is equivalent to just asking that the degree-zero BV cohomologies of the two theories coincide, making this kind of equivalence weaker.

In this paper we study this question for EH and PC models of gravity in any space-time dimension greater than 2, assuming that the metric encoded in the BFV data ${\mathfrak {F}}^\partial$ is nondegenerate (i.e. assuming that the manifold $\Sigma$ is either spacelike or timelike but not lightlike). In the case of EH, we show that ${\mathfrak {F}}$ and ${{\,\mathrm{\textit{AKSZ}}\,}}({{\,\mathrm{\textit{BFV}}\,}}({\mathfrak {F}}))$ are effectively equivalent, with the former being actually the first-order formulation of the latter.

In the case of PC in three dimensions, where ${{\,\mathrm{\textit{BFV}}\,}}({\mathfrak {F}})={\mathfrak {F}}^\partial$ holds, we show that ${{\,\mathrm{\textit{AKSZ}}\,}}({{\,\mathrm{\textit{BFV}}\,}}({\mathfrak {F}}))$ and ${\mathfrak {F}}$ are strongly BV equivalent, which is not unexpected, since PC is strongly BV equivalent to *BF* theory [CS19a, CSS18], and the latter is a topological AKSZ theory. Instead, for higher dimensional PC theory we show that ${{\,\mathrm{\textit{AKSZ}}\,}}({\mathfrak {F}}^\partial )$ and ${\mathfrak {F}}$ are classically equivalent,^5^ with ${\mathfrak {F}}^\partial$ the BFV data constructed from the reduced phase space of PC theory [CCS20, CS19c]. This case is particularly interesting because the BV–BFV construction for PC is obstructed in dimension 4 (and presumably higher). The data ${{\,\mathrm{\textit{AKSZ}}\,}}({\mathfrak {F}}^\partial )$ resulting from the AKSZ construction is a new BV theory defined on cylinders that is still classically equivalent to EH, but also compatible with the BV–BFV formalism (by construction via the AKSZ procedure). Classically, it is simply PC on a smaller space of fields, where part of the torsion-free condition is imposed a priori instead of through the Euler–Lagrange equations.

Our result addresses the problem presented in [CS19b], where it was pointed out that PC theory in dimension greater than three must be complemented with requirements on field configurations at the boundary in order to induce a well-defined BV–BFV structure. One possible way to construct a BV–BFV structure for PC theory is to assume vector fields generating diffeomorphisms transversal to the boundary to vanish at the boundary. Denote by $\underline{{\mathfrak {F}}}$ the resulting BV theory. In [CS19b, Section 5, Remark 34] this was shown to be insufficient to describe the full reduced phase space of GR, as the Hamiltonian constraint is lost in the process: this means that $BFV(\underline{{\mathfrak {F}}})\not ={\mathfrak {F}}^\partial$. Alternatively one may require certain components of the Lorentz connection to vanish on the boundary, although this condition is not natural for general manifolds. One way of reading our paper is to make these conditions natural on cylindrical manifolds, in the sense that we present a version of PC theory, with the compatibility requirements already implemented. In fact, the resulting AKSZ theory has the same equations of motion and the same symmetries, but the AKSZ procedure restricts the moduli space of solutions to an open subset. This is akin to restricting to globally hyperbolic solutions. One can think of the extra required conditions as imposing part of the equations of motion that fix $\omega$ to be the Levi-Civita connection for the metric induced by a tetrad *e*. This is discussed in Sect. 4.2.

Let us stress that having a well-defined BV–BFV structure is a necessary requirement for the quantisation of BV theories with boundary [CMR18]. The fact that the boundary-compatible AKSZ version of PC theory is (possibly) only classically equivalent to the original PC formulation reinforces the idea that care must be placed when attempting BV quantisation of the latter.

A related approach is the ‘parent formulation’ by Barnich and Grigoriev [BG11, Gri11] which derives an AKSZ construction of the BV theory from the jet space formalism (trivariational complex). What is crucially different in our construction is that we consider, as a target, a symplectic description of the classical boundary states. This involves a careful symplectic reduction of the naively associated boundary spaces.^6^ The result of our construction is not only a BV reformulation of the original bulk theory, but a reformulation that is compatible with the boundary as a 1-extended BV–BFV theory (see Definition 5), which is the starting point for quantum (or at least semiclassical) considerations for a theory with boundary [CMR18].

For the same reason, unlike the presymplectic AKSZ formulation presented by Alkalaev and Grigoriev [AG14] and Grigoriev [Gri16], our BV–BFV description of PC gravity is based on a symplectic structure, which is essential for quantization. This does not arise directly from a reduction of the natural presymplectic BFV structure derived from BV in the bulk, which is impossible for $N\ge 4$ as shown in [CS19b], but it is the symplectic BFV structure [CCS20] that resolves the reduced phase space of the theory.

Finally, note that in this paper we consider two separate applications of the AKSZ “reconstruction” of a parametrization-invariant bulk BV theory from its boundary BFV structure, respectively for two formulations of GR (EH and PC). We do not discuss the equivalence between EH and PC, but we investigate the appropriate BV equivalence between each formulation and its own AKSZ “reconstruction.”

These considerations do not exclude, however, some deeper connection between our construction and the ones mentioned above, which are definitely worth exploring.

The paper is organised as follow. In Sects. 1.1 and 1.2 we will outline the BV–BFV and AKSZ constructions, while Sect. 2 is a brief review of the construction of the BFV data for Einstein–Hilbert and Palatini–Cartan theories of gravity, as presented respectively in [CS16, CCS20].

Finally, in Sects. 3 and 4 we will apply the AKSZ construction to the BFV data of EH and PC gravity, respectively, and compare it with the BV data for the two formulations as presented in [CS16, CS19b].

### Relevance and outlook

This work is intended as a first reaping, as a result of a few years of sowing, in a program directed at an analysis of classical General Relativity seen through the lens of the BV formalism with boundary, an attempt at formalising its quantisation within the BV–BFV formalism [CMR18]. The program was initiated in [CS16, CS19b, Sch15], where a few inconsistencies in the behaviour of GR in the presence of boundaries in dimension 4 were detected, and was later extended in [CCS20, CS19a, CSS18], where the comparison with the three dimensional analogue was made.

This series of works is motivated by the obstruction encountered in defining the BV–BFV data for Palatini–Cartan gravity, a requirement for the BV quantisation program with boundary, which has otherwise provided very reliable and flexible (see [CMR18, CMR20] for the quantisation of BF theory, [IM19] for Yang–Mills theory in dimension 2, [CMW17, CMW19a] for split Chern–Simons theory and [CMW19b] for a general approach to a class of AKSZ models, including the Poisson sigma model). No obstruction to BV quantisation with boundary is otherwise present for Einstein–Hilbert theory, and this discrepancy points at the fact that classical equivalence of field theories might be too coarse a classification to have bearing on the respective quantum theories.

The results contained in this paper close the circle, so to speak, in the comparison of classical BV general relativity with boundary, between EH and PC formulations. As a matter of fact, while the AKSZ construction for EH theory is effectively equivalent to the BV theory analysed in [CS16], this is not the case in PC theory analysed in [CS19b] (it is only included within). This fact, together with the equivalence of the reduced phase spaces for EH and PC theories [CCS20, CS19c], can be interpreted as a confirmation that BV Palatini–Cartan theory *must* be supplemented with additional requirements on fields, or otherwise restricted, in order to be viable for BV quantisation. The requirements we find, summarised by Definition 52 are conditions on the Lorentz connection and its conjugate variable, which effectively restrict the space of fields. We find that these conditions are somewhat natural on cylinders.

The very ultimate goal of the construction presented in this paper is the grail of quantisation of gravity. We do not attempt doing it here. What we present is the preliminary setting for a perturbative quantisation on cylinders resulting in the quantum evolution operator from the initial to the final quantum space of states. Due to the degeneracy of the actions (related to gauge invariance), one needs a formalism that allows imposing gauge fixings and checking that the results are independent thereof, up to equivalences that are under control. In the absence of free boundaries (i.e., in the computation of partition functions and expectation values), there are several good methods to do this, including BRST and BV. In the presence of boundary, the best developed method is a compatible combination of BV in the bulk and BFV on the boundary. The compatibility is the main issue here, and this paper discusses it in the context of GR theories.

Performing the actual quantisation, which is far beyond the scope of this paper, implies choosing a polarization on the boundary and a gauge fixing in the bulk, computing the resulting propagators, regularizing the theory, and performing renormalisation in a compatible way with the BV–BFV data (the quantum master equation, and its version with boundary [CMR18]). In the case of gravity, one of course expects an infinite number of independent counterterms to be taken care of. Clever or miraculous ways to keep them under control are the same issue as in other treatments (without boundary): we do not claim to have a better recipe for this issue, but just to have a method to incorporate free boundaries. A minimal way to proceed, as, e.g., in [BFR16], is to allow for infinite counterterms (which is algebraically possible and allows for the construction of families of effective theories, even though the predictivity at all energies is missing).

Naturally, since the outlook of this extended program is that of addressing quantisation of General Relativity (with boundary), we wish to stress that without the observations produced in this preliminary phase, an early attempt at directly quantising PC theory might have been thwarted by the very obstructions highlighted by our investigations.

In this sense, we believe the correct preparation of a field theory for its perturbative quantisation to be of crucial importance to drive the scientific effort towards sensible questions, and divert it when evidence is presented of a potential roadblock ahead. This should be of particular interest for the scientific community heavily involved with the study of Palatini–Cartan theory as a fundamental building block for a quantum theory of gravity.

## Background

One of the goals of this paper is the construction of a BV theory on a cylindrical manifold $\Sigma \times I$ by means of the AKSZ construction, with target a BFV theory associated to $\Sigma$. In this section we introduce the basic definition of the BV(-BFV) and AKSZ formalisms, together with the relevant notions of equivalence that will allow us to compare theories. We refer to [BF83, BV77, BV81, CMR11, CMR14] for a more detailed introduction and more insight in the meaning and the motivations for the following definitions and theorems. For an introduction of the BV (-BFV) formalism and gravity see [CS19a, CS17, CS19c]. Other versions and interpretations of the BV formalism for gravity can be found in [BFR16].

### The Batalin–Vilkovisky formalism

#### Definition 1

A BV theory is a quadruple ${\mathfrak {F}}= \left( {\mathcal {F}}, S, \varpi , Q\right)$ where ${\mathcal {F}}$ is a graded manifold (the *space of BV fields*) endowed with a degree $-1$ symplectic form $\varpi$, $S{:}\,{\mathcal {F}}\rightarrow {\mathbb {R}}$ is a degree 0 functional (the *BV action*) and *Q* is the (odd) Hamiltonian vector field of *S* with respect to $\varpi$ satisfying $[Q,Q]=0$.

#### Remark 2

Since *Q* is the Hamiltonian vector field of *S*, i.e. $\iota _{Q} \varpi = \delta S$ where $\delta$ is the de Rham differential on ${\mathcal {F}}$ and $\iota _Q$ is the contraction w.r.t. *Q*, we can rewrite the equation $[Q,Q]=0$ as $(S,S)=0$ where $(\cdot ,\cdot )$ denotes the Poisson bracket defined by $\varpi$. The latter equation is called the Classical Master Equation (CME).

#### Definition 3

An exact BFV theory is a quadruple ${\mathfrak {F}}^{\partial }= \left( {\mathcal {F}}^{\partial }, S^{\partial }, \varpi ^{\partial }, Q^{\partial }\right)$ where ${\mathcal {F}}^{\partial }$ is a graded manifold (the *space of boundary fields*) endowed with a degree-0 exact symplectic form $\varpi ^{\partial }= \delta \alpha ^{\partial }$, $S^{\partial }{:}\,{\mathcal {F}}^{\partial } \rightarrow {\mathbb {R}}$ is a degree 1 functional and $Q^{\partial }$ is the Hamiltonian vector field of $S^{\partial }$ with respect to $\varpi ^{\partial }$ such that $[Q^{\partial },Q^{\partial }]=0$.

#### Remark 4

Typical examples of BV and BFV theories are modeled on sections of bundles over differentiable manifolds, possibly with boundary, with $\varpi ^{(\partial )},S^{(\partial )}$ and $Q^{(\partial )}$ respectively a local two-form, functional and vector field. Throughout the paper, when specifying BV theories, we will assume that the equations $\iota _Q\varpi =\delta S$ and $(S,S)=0$ are satisfied only up to boundary terms. The failure of said equations will be controlled by the data of a BV–BFV theory, as follows. It is often convenient, in this scenario, to define the slightly more general concept of a *relaxed* BV theory, i.e. data ${\mathfrak {F}}=({\mathcal {F}},S,\varpi ,Q)$ as in Definition 3, but without the requirement that *Q* be the Hamiltonian vector field of *S*. If we are given a BV theory on a closed manifold without boundary, we can consider the same local data as a relaxed BV theory on a manifold with boundary.

#### Definition 5

[CMR14]. A (relaxed) BV theory ${\mathfrak {F}}=\left( {\mathcal {F}},S, \varpi , Q\right)$ is said to be 1-extended to the BFV theory ${\mathfrak {F}}^\partial =\left( {\mathcal {F}}^{\partial }, S^{\partial }, \varpi ^{\partial }, Q^{\partial }\right)$ if there exists a surjective submersion $\pi {:}\,{\mathcal {F}} \rightarrow {\mathcal {F}}^{\partial }$, such that the following compatibility relation is satisfied:

1a $$\begin{aligned} \iota _{Q} \varpi = \delta S + \pi ^{*} \alpha ^{\partial } \end{aligned}$$

The data ${\mathfrak {F}}^{\uparrow 1}=\left( {\mathfrak {F}},{\mathfrak {F}}^\partial ,\pi \right)$ will be called 1-extended BV–BFV theory.

#### Remark 6

Notice that, from the data above, the following relation follows:

1b $$\begin{aligned} \iota _{Q} \iota _{Q} \varpi = 2 \pi ^{*}S^{\partial }. \end{aligned}$$

The following definitions compare two different BV (or BFV) theories.

#### Definition 7

Two B(F)V theories ${\mathfrak {F}}_1^{(\partial )}$ and ${\mathfrak {F}}_2^{(\partial )}$ are said to be strongly B(F)V-equivalent if there exists a symplectomorphism

$$\begin{aligned} \Phi {:}\,({\mathcal {F}}_1^{(\partial )}, \varpi _1^{(\partial )} ) \rightarrow ({\mathcal {F}}_2^{(\partial )}, \varpi _2^{(\partial )}) \end{aligned}$$

preserving the BV action: $\Phi ^* S_2^{(\partial )} = S_1^{(\partial )}$. The map $\Phi$ is called a strong B(F)V-equivalence.

#### Definition 8

Let ${\mathfrak {F}}_1$ and ${\mathfrak {F}}_2$ be two (relaxed) BV theories. A (relaxed) BV-inclusion ${\mathfrak {I}}{:}\, {\mathfrak {F}}_1 \rightarrow {\mathfrak {F}}_2$ is an inclusion of (super)manifolds $\iota {:}\,{\mathcal {F}}_1 \rightarrow {\mathcal {F}}_2$ such that $\varpi _1 = \iota ^* \varpi _2$ and $\iota ^*Q_1 = Q_2\iota ^*$. If the two theories are relaxed we will additionally require $\iota ^* S_2 = S_1$. In this case we say that ${\mathfrak {F}}_1$ is a BV-subspace^7^ of ${\mathfrak {F}}_2$.

#### Remark 9

Naturally, if $Q_1$ and $Q_2$ are the Hamiltonian vector fields of $S_1$ and $S_2$ respectively, the condition $\iota ^* S_2 = S_1$ is equivalent to the condition $\iota ^* Q_2 = Q_1 \iota ^*$, up to a constant.

#### Proposition 10

The composition of a strong BV equivalence and a BV inclusion is in turn a BV inclusion.

#### Proof

The map $\Phi \circ \iota$ satisfies trivially the properties of a BV inclusion. $\square$

A notion that we will need to compare theories is that of BV-pushforward. This notion is usually phrased at the quantum level [CMR18, Mne17], where the additional data of a BV Laplacian needs to be provided. However here we are interested mainly in its classical counterpart. The basic setting is the same, although we consider the following simplifying assumptions. Suppose that we have a splitting of a graded symplectic manifold $({\mathcal {F}}, \varpi )$ so that ${\mathcal {F}}= {\mathcal {F}}' \times {\mathcal {F}}''$, with $\varpi = \varpi '+\varpi ''$, and let ${\mathcal {L}}$ be a Lagrangian submanifold of $({\mathcal {F}}'', \varpi '')$ endowed with a half-density $\mu$ on ${\mathcal {F}}''$, which thus defines by restriction a density $\mu _{{\mathcal {L}}}$ on ${\mathcal {L}}$. Denote coordinates $(z',z'')$ respectively in ${\mathcal {F}}',{\mathcal {F}}''$, and let $z''\in \{x,x^\dag \}$ be Darboux adapted coordinates such that *x* parametrises ${\mathcal {L}}$ and $x^\dag$ are transversal.

#### Definition 11

We define the Batalin–Vilkovisky–Legendre transform of a functional $S\in C^\infty ({\mathcal {F}})$, with respect to the Lagrangian ${\mathcal {L}}\subset {\mathcal {F}}''$, as $S_{\text {BVL}}\in C^\infty (\mathcal {F'})$:

2 $$\begin{aligned} S_{\text {BVL}} = S(z,x_0, x^\dag =0) \end{aligned}$$

where $x_0$ is a critical point for *S* (assumed unique):

$$\begin{aligned} \frac{\delta S}{\delta x}\bigg \vert _{x^\dag =0}(x_0)=0. \end{aligned}$$

Starting from a BV theory on $({\mathcal {F}}, \varpi )$ we build a theory on $({\mathcal {F}}', \varpi ')$ by means of a gauge-fixed fiber integral along ${\mathcal {F}}''$, with gauge-fixing Lagrangian ${\mathcal {L}}$. In other words, if *S* denotes a BV action on ${\mathcal {F}}$ we consider the effective result of the BV pushforward (fiber integral) to be

3 $$\begin{aligned} \exp \left( \frac{i}{\hbar }S_{\text {eff}}\right) :=\int _{{\mathcal {L}}\subset {\mathcal {F}}''} \exp \left( \frac{i}{\hbar }S\right) \bigg \vert _{{\mathcal {L}}}\mu _{{\mathcal {L}}} \end{aligned}$$

where the integral is defined perturbatively as a power series in $\hbar$. Note that $S_\text {BVL}$ is the dominant term of $S_\text {eff}$. When *S* depends only quadratically on the variables on ${\mathcal {L}}$, the only correction is $i\hbar /2$ times the logarithm of the determinant of the quadratic form.

#### Remark 12

Let us comment briefly on the notion of equivalence of theories in the BV formalism. When the moduli spaces of solutions of the equations of motion for two theories coincide, the theories are said to be classically equivalent. A finer notion of equivalence requires that the BV cohomologies be isomorphic,^8^ and it allows for an extension to the case with boundary. One looks at the bicomplex given by the BV operator and the de Rham differential $(\Omega ^{\bullet ,\bullet }({\mathcal {F}}_i,M), Q_i-d)$, and equivalence in this sense requires two theories to have quasi-isomorphic local de Rham/BV complexes. The typical argument for equivalence involves the elimination of so-called generalised auxiliary fields and trivial pairs [BBH95, Hen90]. This notion, however, is suboptimal because it relies on homological perturbation theory, which potentially could output an infinite tower of ghosts and antighosts in the process of constructing an equivalent theory. In other words, that approach—albeit somewhat well-established—does not provide a direct answer to the question of whether two *given* BV theories (in the form of two Hamiltonian dg-manifolds) are equivalent, provided their degree-0 sectors are classically equivalent. For this, instead, a spectral sequence argument would be more appropriate.^9^

On the other hand, one could phrase equivalence in the BV–BFV formalism, requiring existence of the BV–BFV structure and equivalence of the respective BV and BFV cohomologies (an explicit example of this, is the strong equivalence of General Relativity and BF theory in three dimensions [CS19a]). This existence requirement might become an obstruction to BV–BFV equivalence^10^. For our purposes, then, even assuming that by removal of generalised auxiliary fields one may prove some BV equivalence between Palatini–Cartan and Einstein–Hilbert theories, the results of [CS19b] and of the present paper show a discrepancy of the theories in the BV–BFV sense.

### The AKSZ construction

Let *X* be a graded manifold and *N* an ordinary manifold and let $\mu _N$ be the canonical Berezinian^11^ on *T*[1]*N*.

#### Definition 13

(*Transgression map*). Consider the map

4 $$\begin{aligned} {\mathfrak {T}}^{(\bullet )}_N {:}\,\Omega ^\bullet (X) \longrightarrow \Omega ^\bullet \left( \mathrm {Map}(T[1]N,X)\right) \end{aligned}$$

defined by ${\mathfrak {T}}^{(\bullet )}_N:=p_*\mathrm {ev}^*$, where5and we set $p_*=\int \limits _N \mu _N$. We will call ${\mathfrak {T}}^{(\bullet )}_N$ the transgression map, and its evaluation a transgression.

We endow the graded manifold *X* with a function *S* of degree *n* and parity $n \mod 2$, together with a one-form $\alpha$ of degree $n-1$ and parity $n-1 \mod 2$, such that $\varpi =d\alpha$ is nondegenerate and $\{S,S\}=0$ with respect to the Poisson structure defined by $\varpi$. Then we say that *X* has a Hamiltonian dg-manifold structure, with differential $\{S,\cdot \}$.

Observing that the de Rham differential $d_{N}$ on *N* can be seen as a degree 1 vector field on $\mathrm {Map}(T[1]N,X)$ we have

#### Theorem 14

[Ale+97]. Let $(X,S,\alpha )$ a dg-manifold as described above. Consider the data

6 $$\begin{aligned} {\mathfrak {F}}^{\textsf {\tiny AKSZ}}(N;X,S,\alpha ):=\left( {\mathcal {F}}^{\textsf {\tiny AKSZ}}, S^{\textsf {\tiny AKSZ}}, \Omega ^{\textsf {\tiny AKSZ}}, Q^{\textsf {\tiny AKSZ}}\right) \end{aligned}$$

with ${\mathcal {F}}^{\textsf {\tiny AKSZ}}=\mathrm {Map}(T[1]N,X)$, $\Omega ^{\textsf {\tiny AKSZ}}:={\mathfrak {T}}^{(2)}_N(\varpi )$, the functional $S^{\textsf {\tiny AKSZ}}{:}\,{\mathcal {F}}^{\textsf {\tiny AKSZ}}\rightarrow {\mathbb {R}}$,

7 $$\begin{aligned} S^{\textsf {\tiny AKSZ}}:={\mathfrak {T}}_N^{(0)}(S) + \iota _{d_{N}}{\mathfrak {T}}_N^{(1)}(\alpha ). \end{aligned}$$

and the cohomological vector field $Q^{\textsf {\tiny AKSZ}}$ such that $\iota _{Q^{\textsf {\tiny AKSZ}}}\Omega ^{\textsf {\tiny AKSZ}}=\delta S^{\textsf {\tiny AKSZ}}$. Then, ${\mathfrak {F}}^{\textsf {\tiny AKSZ}}(N;X,S,\alpha )$ defines a BV theory.

We will call ${\mathcal {F}}^{\textsf {\tiny AKSZ}}:=\mathrm {Map}(T[1]N,X)$ the *AKSZ space of fields*. Introducing Darboux coordinates $\{p_i, q^i\}$ in *X* so that $\alpha = p_i dq^i$, the space of AKSZ fields is composed of inhomogeneous differential forms ${\mathfrak {P}}, {\mathfrak {Q}}$ on *N*. Then, if we consider *X* to be the space of sections of a bundle $E\rightarrow \Sigma$, that is to say $X=T^*[n-1]C^\infty (\Sigma ,E)$, we can write

8 $$\begin{aligned} \Omega ^{\textsf {\tiny AKSZ}} = \int \limits _{\Sigma \times N} \left[ \langle \delta {\mathfrak {P}}, \delta {\mathfrak {Q}}\rangle \right] ^{\mathrm {top}} \equiv \int \limits _{\Sigma \times N} \left[ \delta {\mathfrak {P}}_i \delta {\mathfrak {Q}}^i \right] ^{\mathrm {top}} \end{aligned}$$

where we have denoted by $\delta$ the deRham differential on spaces of maps and $C^\infty$-sections, and $\text {top}$ denotes the top-form parts of the inhomogeneous differential forms within brackets. We will drop the superscript $\text {top}$ in what follows.

Consider this elementary fact:

#### Lemma 15

Let *A*, *B*, *C* be graded manifolds, $\phi {:}\,B\rightarrow C$ an isomorphism of graded manifolds, and $\mu _A$ a measure on *A*. Consider the diagram9Then, setting ${\pi _{B}}_* = \int \mu _A \cdot$ and ${\pi _{C}}_* = \int \mu _A \cdot$, we have $\phi ^*\circ {\pi _{C}}_* = {\pi _{B}}_*\circ (\mathrm {id}\times \phi )^*$.

#### Theorem 16

Let $(X,S_X,\alpha _X)$ and $(Y,S_Y,\alpha _Y)$ be equivalent Hamiltonian dg-manifolds, i.e. there exists a diffeomorphism $\phi {:}\,X\rightarrow Y$ such that $\varpi _X=\phi ^*\varpi _Y$, and $S_X=\phi ^*S_Y$. Then ${\mathfrak {F}}^{\textsf {\tiny AKSZ}}(N;X,S_X,\alpha _X)$ and ${\mathfrak {F}}^{\textsf {\tiny AKSZ}}(N;Y,S_Y,\alpha _Y)$ are strongly equivalent BV(-BFV) theories for every manifold *N*.

#### Proof

$\phi {:}\,X\rightarrow Y$ induces an isomorphism

$$\begin{aligned} {\tilde{\phi }}{:}\, \mathrm {Maps}(T[1]N,X) \rightarrow \mathrm {Maps}(T[1]N,Y) \end{aligned}$$

by precomposing maps with $\phi ^*$ or ${\phi ^{-1}}^*$. Then, we can apply Lemma 15 with $B=\mathrm {Maps}(T[1]N,X)$, $C= \mathrm {Maps}(T[1]N,Y)$ and $A=T[1]N$. $\square$

### One-dimensional AKSZ construction

Let $I \subset {\mathbb {R}}$ be an interval, and ${\mathfrak {F}}^\partial$ an exact BFV theory. We can construct a BV theory by applying Theorem 14 on the Hamiltonian dg-manifold underlying an exact BFV theory:

$$\begin{aligned} {\mathfrak {F}}^{\textsf {\tiny AKSZ}}(I;{\mathfrak {F}}^\partial ):={\mathfrak {F}}^{\textsf {\tiny AKSZ}}(I;{\mathcal {F}}^\partial ,S^\partial ,\alpha ^\partial ). \end{aligned}$$

The resulting space of fields reads

$$\begin{aligned} {\mathcal {F}}^{\textsf {\tiny AKSZ}}= \text {Map}(T[1]I, {\mathcal {F}}^{\partial }). \end{aligned}$$

Since the target space ${\mathcal {F}}^{\partial }$ is (locally) a graded vector space, we identify the space of AKSZ fields with

$$\begin{aligned} {\mathcal {F}}^{\textsf {\tiny AKSZ}}= \Omega ^{\bullet }(I) \otimes {\mathcal {F}}^{\partial }. \end{aligned}$$

In particular, when ${\mathcal {F}}^{\partial }$ is modeled on sections of some bundle over a $(N-1)$-dimensional manifold $\Sigma$, we can view ${\mathcal {F}}^{\textsf {\tiny AKSZ}}$ as the space of sections of some (graded) bundle over $\Sigma \times I$. The space $\Omega ^{\bullet }(I)$ splits into:

$$\begin{aligned} \Omega ^{\bullet }(I) = C^{\infty }(I) \oplus \Omega ^1(I)[-1] \end{aligned}$$

hence, to each field in ${\mathcal {F}}^{\partial }$ we associate two new fields. For simplicity we denote the field in $C^{\infty }(I)\otimes {\mathcal {F}}^{\partial }$ with the same letter as the old one, and use another letter for the one in $\Omega ^1 [-1](I)\otimes {\mathcal {F}}^{\partial }$.

#### Proposition 17

[CMR18, CMR14]. Let ${\mathfrak {F}}^\partial (\Sigma )=({\mathcal {F}}_{}^\partial (\Sigma ),S_{}^\partial (\Sigma ), \varpi _{}^\partial (\Sigma ),Q_{}^\partial (\Sigma ))$ be an exact BFV theory, with ${\mathcal {F}}_{}^\partial (\Sigma ):=\Gamma (E\rightarrow \Sigma )$, and $\varpi _{}^\partial (\Sigma )=\delta \alpha _{}^\partial (\Sigma )$. Then, if $I:=[0,1]$ we have that ${\mathfrak {F}}^{\textsf {\tiny AKSZ}}(I;{\mathfrak {F}}^\partial (\Sigma ))$ is a 1-extended BV–BFV theory over ${\mathfrak {F}}^\partial (\Sigma )$ (see Definition 5).

#### Proof

Theorem 14 tells us that ${\mathfrak {F}}^{\textsf {\tiny AKSZ}}(I;{\mathfrak {F}}^\partial (\Sigma ))$ is a BV theory (up to boundary terms). If we parametrise fields in ${\mathcal {F}}^{\textsf {\tiny AKSZ}}$ as

$$\begin{aligned} {\mathfrak {P}}= p(t) + q^\dag (t)dt \quad {\mathfrak {Q}} = q(t) + p^\dag (t)dt \end{aligned}$$

we get

$$\begin{aligned} \Omega ^{\textsf {\tiny AKSZ}} = \int \limits _{\Sigma \times I } \langle \delta {\mathfrak {P}}, \delta {\mathfrak {Q}}\rangle = \int \limits _{\Sigma \times I} \left\{ \langle \delta p, \delta p^\dag \rangle + (-1)^{|q|+1} \langle \delta q, \delta q^\dag \rangle \right\} dt \end{aligned}$$

and

$$\begin{aligned} S^{\textsf {\tiny AKSZ}} = \int \limits _{\Sigma \times I} \langle p, d_Iq\rangle +[{\mathfrak {T}}_I^{(0)}(S^\partial (\Sigma )) ]^{\mathrm {top}}. \end{aligned}$$

The transgressed integrand needs to be first-order in *dt*, which leaves us with

$$\begin{aligned}{}[{\mathfrak {T}}_I^{(0)}(S^\partial (\Sigma ))]^{\mathrm {top}} \equiv S^\partial (\Sigma )[p+q^\dag dt,q+p^\dag dt] = \frac{\delta S^\partial (\Sigma )}{\delta p}(q^\dag dt) + \frac{\delta S^\partial (\Sigma )}{\delta q}(p^\dag dt) \end{aligned}$$

Then $Q^{\textsf {\tiny AKSZ}}$ splits in a transversal part plus a *tangential* one: $Q^{\textsf {\tiny AKSZ}} = Q^T + {\hat{Q}}$, where $Q^Tq^\dag = -{\dot{p}}$ and $Q^T p^\dag = {\dot{q}}$ is essentially just deRham differential on *I*, and ${\hat{Q}}$ is easily obtained:

$$\begin{aligned} {\hat{Q}} p= & {} \frac{\delta S^\partial (\Sigma )}{\delta q} \equiv Q^\partial p \quad {\hat{Q}} q = \frac{\delta S^\partial (\Sigma )}{\delta p}\equiv Q^\partial q \\ {\hat{Q}} p^\dag= & {} \frac{\delta ^2S^\partial (\Sigma )}{\delta q \delta p}(p^\dag ) + \frac{\delta ^2S^\partial (\Sigma )}{\delta p^2}(q^\dag ) \quad {\hat{Q}} q^\dag = \frac{\delta ^2S^\partial (\Sigma )}{\delta p\delta q}(q^\dag ) + \frac{\delta ^2S^\partial (\Sigma )}{\delta q^2} (p^\dag ). \end{aligned}$$

The boundary terms are easily seen in the given local chart, in fact:

$$\begin{aligned} \iota _{Q^{\textsf {\tiny AKSZ}}} \Omega ^{\textsf {\tiny AKSZ}} = \delta S^{\textsf {\tiny AKSZ}} + {\check{\alpha }} \end{aligned}$$

but ${{\check{\alpha }}}$ only sees contributions from $\langle p, dq\rangle$ and, up to sign, we get ${{\check{\alpha }}} = \alpha ^\partial (\Sigma )$, with $\delta \alpha ^\partial (\Sigma ) = \varpi ^\partial _{\Sigma }$. Then, the projection of $Q^{\textsf {\tiny AKSZ}}$ along the natural projection map from ${\mathcal {F}}^{AKSZ}$ to the space of boundary fields, which coincides with ${\mathcal {F}}^\partial (\Sigma )$, is precisely $Q_{}^\partial (\Sigma )$, concluding the argument. $\square$

#### Remark 18

A similar statement to Proposition 17 is presented in Henneaux–Bunster [HT92, Theorem 18.4.5], where one identifies the output of the above AKSZ construction with the (first-order) BV theory obtained by embedding in the BV formalism the generalised Hamiltonian formulation of a given field theory (see also [DGH90]). An analogous construction, already in the context of AKSZ theories, was presented in [BG05, GD00]. The added observation of Proposition 17 is the compatibility between BV for the bulk and BFV on the boundary, viz., what we call a 1-extended BV–BFV theory in Definition 5.

We would like to show that this construction behaves well under equivalences of the relevant BFV data.

#### Corollary 19

(Theorem 16). Let ${\mathfrak {F}}^\partial _1$ and ${\mathfrak {F}}^\partial _2$ be two strongly BFV-equivalent (exact) theories, then ${\mathfrak {F}}^{\textsf {\tiny AKSZ}}(I;{\mathfrak {F}}_1^\partial )$ and ${\mathfrak {F}}^{\textsf {\tiny AKSZ}}(I;{\mathfrak {F}}_2^\partial )$ are strongly BV-equivalent.

#### Proof

A strong BV equivalence induces an isomorphism of the underlying dg-manifolds.

$$\square $$

## BFV Theories of Gravity

### BFV Einstein–Hilbert theory

The BFV theory for GR in the Einstein–Hilbert formalism (as described in [CS16]) associates to any $(N-1)$-dimensional (pseudo)-Riemannian^12^ manifold $\Sigma$ the graded 0-symplectic manifold

10 $$\begin{aligned} {\mathcal {F}}_{EH}^\partial (\Sigma ) = T^*\underbrace{\left( S_{nd}^2(T\Sigma ) \times {\mathfrak {X}}[1](\Sigma ) \times C^\infty [1](\Sigma )\right) }_{\{{\gamma }, \xi ^\partial ,\xi ^n\}}, \end{aligned}$$

where $S^2_{nd}(\Sigma )$ denotes the space of nondegenerate symmetric tensor fields of rank two, with canonical exact symplectic structure:

11 $$\begin{aligned} \varpi _{EH}^\partial (\Sigma ) = \delta \alpha _{EH}^\partial (\Sigma ) = \delta \int \limits _{\Sigma } \langle {\Pi }, \delta {\gamma }\rangle + \langle \varphi _\partial ,\delta \xi ^\partial \rangle + \langle \varphi _n,\delta \xi ^n\rangle , \end{aligned}$$

and $\{\Pi ,\varphi _\partial ,\varphi _n\}$ denote variables in the cotangent fiber, dual to $\{\gamma ,\xi ^\partial ,\xi ^n\}$ respectively, i.e.

$$\begin{aligned} {\Pi }&\in S^2(T^*\Sigma )\otimes \mathrm {Dens}(\Sigma ),\\ \varphi _\partial&\in \Omega ^1(\Sigma )\otimes \mathrm {Dens}(\Sigma ),\\ \varphi _n&\in C^\infty (\Sigma )\otimes \mathrm {Dens}(\Sigma ). \end{aligned}$$

#### Remark 20

The components $(\gamma )^{ab}$ of a ${\gamma }\in S_{nd}^2(T\Sigma )$ can be thought of as the inverse of a (pseudo-)Riemannian metric on $\Sigma$, which we denote by $\gamma ^{-1}$. With a slight abuse of notation^13^ we will denote by $\sqrt{{\gamma }}=\sqrt{\det (\gamma _{ab})}$ the square root of the determinant of the metric on $\Sigma$ that we denote by ${\gamma }^{-1}$ everywhere else. In other words, $\sqrt{{\gamma }}$ is the usual density associated to a metric, i.e. $\sqrt{{\gamma }}\,\mathrm {d^{N-1}x}$ is a volume form on $\Sigma$. Observe that all fields in the fibres of the cotangent bundle (10) are sections of the respective dual bundles, tensored with densities. The conjugate field to $\gamma$ is a section of the second symmetric tensor power of the cotangent bundle of $\Sigma$ tensored with densities on $\Sigma$, i.e. ${\Pi }\in S^2(T^*\Sigma )\otimes \mathrm {Dens}(\Sigma )$ is of the form $\Pi = \sqrt{{\gamma }}\pi$, for $\pi \in S^2(T^*\Sigma )$. A similar decomposition holds for $\varphi _\partial ,\varphi _n$.

In addition to ${\mathcal {F}}_{EH}^\partial (\Sigma )$ and $\varpi _{EH}^\partial (\Sigma )$, the BV–BFV procedure outputs a functional of degree 1 on ${\mathcal {F}}_{EH}^\partial (\Sigma )$, called BFV action. It is given by the local expression

12 $$\begin{aligned} S_{EH}^\partial (\Sigma )=&\int \limits _{\Sigma } \left\{ H_n\xi ^n + \langle {\Pi }, L_{\xi ^\partial }{\gamma }\rangle + {\varphi }_nL_{\xi ^\partial }\xi ^n - {\gamma }({\varphi }_\partial ,d\xi ^n)\xi ^n + \left\langle {\varphi }_\partial ,\frac{1}{2}[\xi ^\partial ,\xi ^\partial ]\right\rangle \right\} \end{aligned}$$

where we have denoted the *Hamiltonian constraint density* by

13 $$\begin{aligned} H_n({\gamma },{\Pi }) = \left( \frac{1}{\sqrt{{\gamma }}}\left( \mathrm {Tr}_{{\gamma }}[{\Pi }^2] - \frac{1}{d-1}\mathrm {Tr}_{{\gamma }}[{\Pi }]^2\right) + \sqrt{{\gamma }}\left( R^\partial -2\Lambda \right) \right) \end{aligned}$$

with $R^\partial$ is the trace of the Ricci tensor with respect to the metric $\gamma ^{-1}$, $\Lambda \in {\mathbb {R}}$ is the cosmological constant, $\mathrm {Tr}_{{\gamma }}[{\Pi }^2]={\gamma }^{ab}{\gamma }^{cd}{\Pi }_{bc}{\Pi }_{ad}$ and $\mathrm {Tr}_{{\gamma }}{\Pi }$ is the density ${\gamma }^{ab}{\Pi }_{ab}$. Observe that we can also denote the *momentum constraint density* as the density-valued one-form

14 $$\begin{aligned} H_\partial {:}\,{\mathfrak {X}}(\Sigma )\rightarrow \mathrm {Dens}(\Sigma )\quad H_\partial (X) = \langle {\Pi },L_{X}{\gamma }\rangle \end{aligned}$$

for $$X\in {\mathfrak {X}}(\Sigma )$$.

#### Remark 21

One can integrate the density of Eq. (13) against a function $\lambda \in C^\infty (\Sigma )$, or integrate the density in (14) to get local functionals on fields. Then $\lambda$ and *X* play the role of Lagrange multipliers, to enforce the so-called Hamiltonian and momentum constraints.

The Hamiltonian vector field $Q_{EH}^\partial (\Sigma )$ of $S_{EH}^\partial (\Sigma )$ with respect to $\varpi _{EH}^\partial (\Sigma )$ is a differential on $C^\infty ({\mathcal {F}}_{EH}^\partial (\Sigma ))$, the BFV differential, and its cohomology in degree zero is the reduced phase space defined by the constraints $\{H_n,H_\partial \}$.

#### Definition 22

We define BFV Einstein–Hilbert theory associated to be the assignment

15 $$\begin{aligned} \Sigma \leadsto {\mathfrak {F}}^\partial _{EH}(\Sigma )=({\mathcal {F}}_{EH}^\partial (\Sigma ),S_{EH}^\partial (\Sigma ),\varpi _{EH}^\partial (\Sigma ),Q_{EH}^\partial (\Sigma )). \end{aligned}$$

### BFV Palatini–Cartan theory

Let $\Sigma$ be an $(N-1)$-dimensional compact and orientable^14^ smooth manifold and let $P \rightarrow \Sigma$ be an $SO(N-1,1)$-principal bundle. Let also ${\mathcal {V}}$ be the associated vector bundle where each fibre is isomorphic to $(V, \eta )$, an *N*-dimensional vector space with a pseudo-Riemannian inner product $\eta$ on it. We further identify $\mathfrak {so}(N-1,1) \cong \bigwedge ^2 {\mathcal {V}}$ using $\eta$.

Furthermore we use the following notation:

$$\begin{aligned} \Omega _{\partial }^{i,j}:= \Omega ^i\left( \Sigma , \textstyle {\bigwedge ^j} {\mathcal {V}}\right) . \end{aligned}$$

The BFV data for PC theory has been described in [CCS20] for $N \ge 4$ and in [CS19a] for $N=3$, the following discussion will be nontrivial for $N\ge 4$, see Remark 28. The classical fields of the theory are then $e \in \Omega _{nd}^1(\Sigma , {\mathcal {V}})$—i.e $\Omega _{\partial }^{1,1}$ plus the nondegeneracy condition that the induced morphism $T\Sigma \rightarrow {\mathcal {V}}$ should be injective—and the equivalence class of a connection $\omega \in {\mathcal {A}}(\Sigma )$ under the *e*-dependent relation $\omega \sim \omega + v$ for *v* such that $W_{e^{N-3}}^{1,1}(v)=0$, where

$$\begin{aligned} W_{e^{N-3}}^{1,1}{:}\,\Omega ^{1,1}_\partial \rightarrow \Omega ^{2,2}_\partial , \quad W_{e^{N-3}}^{1,1}(v) = e^{N-3}\wedge v. \end{aligned}$$

We denote this equivalence class and the quotient space it belongs to by $[\omega ] \in {\mathcal {A}}^{\mathrm {red}}(\Sigma )$. We further assume that the boundary metric

16 $$\begin{aligned} g^\partial _{ij}:=\eta (e_i,e_j) \end{aligned}$$

is nondegenerate.

The symplectic manifold $F^\partial _{PC}$ of (degree-0) boundary fields for PC theory is then the total space of the fibre bundle $F^\partial _{PC} \longrightarrow \Omega ^1_{\text {nd}}(\Sigma ,{\mathcal {V}})$, with fiber ${\mathcal {A}}^{\mathrm {red}}$. The manifold $F^\partial _{PC}$ is obtained as the symplectic reduction of the naive boundary two-form ${\check{\varpi }}=\int e^{N-3}\delta e\delta \omega$, which is pre-symplectic since $\mathrm {ker}({\check{\varpi }}) \simeq \mathrm {ker}(W^{1,2}_{e^{N-3}})\not =\{0\}$, as described in [CS19c]. Instead of working with equivalence classes of connections, it is convenient to fix a nonvanishing section $\epsilon _n\in \Gamma ({\mathcal {V}})$ and enforce a condition called *structural constraint*, which was introduced in [CCS20]:

17 $$\begin{aligned} (N-3)\epsilon _n e^{N-4} d_{\omega } e \in \text {Im} W_{e^{N-3}}^{1,1}, \end{aligned}$$

in the space of boundary tetrads and connections. In order to do this one restricts to tetrads *e* that are linearly independent from $\epsilon _n$. In general this implies working in patches over the space of the *e*-fields. However, if $g^\partial$ is space-like, we may choose once and for all $\epsilon _n$ to be time-like, which provides a global choice on the space of the *e*-fields.

#### Remark 23

Considering the boundary by itself, the constraint (17) is one of the possible ways to fix the transformations in the kernel of the presymplectic form. If we take the bulk as well into account, it assumes a more fundamental role: it is the necessary and sufficient condition for the transversal equations of motions to be solvable. Indeed, the (bulk) equations of motion split into a *tangential* equation

$$\begin{aligned} e^{N-3} d_\omega e = 0 \end{aligned}$$

and a transversal one

$$\begin{aligned} (N-3)e_n e^{N-4} d_\omega e = e^{N-3} (d_\omega e)_n, \end{aligned}$$

which tells us that $e_n e^{N-4} d_\omega e$ must be in the image of $W_{e^{N-3}}^{1,1}$. Then, upon using the tangential equation, we can replace $e_n$ with some fixed $\epsilon _n$. See Sect. 4.2.

Denoting by ${\mathsf {S}}\subset \Omega _{nd}^1(\Sigma ,{\mathcal {V}})\times {\mathcal {A}}(\Sigma )$ the submanifold defined by Eq. (17), we have:

#### Proposition 24

[CCS20, CS19c]. There exists a symplectomorphism

$$\begin{aligned} {F}^\partial _{PC}(\Sigma ) \longrightarrow {\mathsf {S}}. \end{aligned}$$

Effectively, then, one can work on ${\mathsf {S}}$. The main advantage of this explicit description of the symplectic space of boundary fields is that it allows to explicitly compute the *symplectic* BFV data for PC theory (see Remark 29).

In order to write down the BFV data it is sufficient to fix the equivalence class $[\omega ]\in {\mathcal {A}}^{\mathrm {red}}(\Sigma )$ using (17) as done in [CCS20], however, when extending $F_{PC}^\partial$ to a graded manifold we can choose to modify the structural constraint by adding terms in the ghosts and antifields. This will turn out to be convenient in what follows.

#### Definition 25

Consider the graded manifold

18 $$\begin{aligned} \check{{\mathcal {F}}}_{PC}(\Sigma ):= \Omega ^{1,1}_{\text {nd}}(\Sigma ,{\mathcal {V}})\times {\mathcal {A}}(\Sigma ) \times T^* \left( \Omega _{\partial }^{0,2}[1]\oplus {\mathfrak {X}}[1](\Sigma ) \oplus C^\infty [1](\Sigma )\right) , \end{aligned}$$

where we denote fields by

- $e \in \Omega ^{1,1}_{\text {nd}}(\Sigma ,{\mathcal {V}})$ and $\omega \in {\mathcal {A}}(\Sigma )$ in degree zero,
- $c \in \Omega _{\partial }^{0,2}[1]$, $\xi \in {\mathfrak {X}}[1](\Sigma )$ and $\lambda \in \Omega ^{0,0}[1]$ in degree one,
- $c^\dag \in \Omega _{\partial }^{3,2}[-1]$, $\lambda ^\dag \in \Omega _{\partial }^{3,4}[-1]$ and $\xi ^\dag \in \Omega _\partial ^{1,0}[-1]\otimes \Omega _{\partial }^{3,4}$ in degree minus one,

together with a fixed section $\epsilon _n \in \Gamma (M,{\mathcal {V}})$, completing the image of *e* to a basis of ${\mathcal {V}}$. We define the *BFV structural constraint* to be condition^15^:

19 $$\begin{aligned} (N-3)\epsilon _n e^{N-4} d_{\omega } e + \left( L_\xi ^\omega \epsilon _n - [c,\epsilon _n]\right) ^{(a)} c^\dag _a \in \text {Im} W_{e^{N-3}}^{1,1} \end{aligned}$$

where $\{(a),(n)\}$ denote components with respect to a basis $\{e_a, \epsilon _n\}$. We define the *space of BFV fields* ${\mathcal {F}}_{PC}^\partial (\Sigma )$ to be the space of solutions of the BFV structural constraints within ${\check{{\mathcal {F}}}}_{PC}(\Sigma )$.

In order to have a better expression of the BFV structure, following [CCS20, Section 5.2], we introduce the field $y^\dag \in \Omega _{\partial }^{3,3}[-1]$ such that the original fields $\lambda ^\dag$ and $\xi ^{\dag '}$ are recovered through $\epsilon _n y^\dag = -\lambda ^\dag$ and $e_a y^\dag = -\xi _a^{\dag '}$. This also allows us to write a single expression for all $N \ge 3$.

To complete the definition of the BFV data for Palatini–Cartan theory we consider a degree 1 functional and a symplectic form^16^ given, respectively, by:

20 $$\begin{aligned} S_{PC}^\partial (\Sigma )&= \int _{\Sigma } c e^{N-3} d_{\omega } e + \iota _{\xi } e e^{N-3} F_{\omega } + \epsilon _n \lambda e^{N-3} F_{\omega } +\frac{1}{2} [c,c] c^{\dag } - L^{\omega }_{\xi } c c^{\dag }\nonumber \\&\quad +\, \,\frac{1}{2} \iota _{\xi }\iota _{\xi } F_{\omega }c^{\dag } -[c, \epsilon _n \lambda ]y^{\dag } + L^{\omega }_{\xi } (\epsilon _n \lambda )y^{\dag } + \frac{1}{2}\iota _{[\xi ,\xi ]}e y^{\dag }, \end{aligned}$$

21 $$\begin{aligned} \varpi _{PC}^\partial (\Sigma )&= \int _{\Sigma } e^{N-3} \delta e \delta \omega + \delta c \delta c^\dag + \delta \omega \delta (\iota _\xi c^\dag ) - \delta \lambda \epsilon _n \delta y^\dag +\iota _{\delta \xi } \delta (e y^\dag ). \end{aligned}$$

Note that each term of the integrals belongs to $\Omega ^{N-1,N}$, which can be canonically identified, using $\sqrt{|\det \eta |}$, with the space of densities on $\Sigma$. A detailed explanation can be found in [CCS20]. However, we will not write down the factor $\sqrt{|\det \eta |}$ explicitly.

#### Definition 26

We define BFV Palatini–Cartan theory to be the assignment

22 $$\begin{aligned} \Sigma \leadsto {\mathfrak {F}}^\partial _{PC}(\Sigma )=({\mathcal {F}}_{PC}^\partial (\Sigma ),S_{PC}^\partial (\Sigma ),\varpi _{PC}^\partial (\Sigma ),Q_{PC}^\partial (\Sigma )). \end{aligned}$$

with $Q_{PC}^\partial (\Sigma )$ the Hamiltonian vector field of $S_{PC}^\partial (\Sigma )$ with respect to $\varpi _{PC}^\partial (\Sigma )$.

#### Remark 27

Notice that the data presented in Definition 26 are equivalent to the BFV data presented in [CCS20]. The cohomological vector field of a function on (the presymplectic manifold) $\check{{\mathcal {F}}}_{PC}(\Sigma )$ is uniquely fixed by the tangency to the BFV structural constraint (19). In [CCS20] the equivalent choice of the constraint (17) is made, and the resulting *Q*’s differ along $\omega$ by a term in the kernel of $W_{e^{N-3}}^{1,2}$.^17^ We make this choice in this paper because it makes it easier to compare the AKSZ construction for the constrained space ${\mathcal {F}}^\partial _{PC}(\Sigma )$ with a constrained version of PC, see Remark 28.

#### Remark 28

In [CS19b] two of the authors showed that the natural BV data for PC theory (in dimension $N\ge 4$) cannot be extended to a BV–BFV theory (Definition 5). Since the degree-0 part of our AKSZ target will be the constrained space ${\mathcal {F}}_{PC}^\partial (\Sigma )$, the extension to the cylinder^18^ of Eq. (19) provides an explicit realisation of (one of) the conditions that must be imposed on the bulk BV fields, in order to have a 1-extended BV–BFV theory. For $N=3$, the additional condition imposed by (17) is void, explaining why 3d PC theory can be (fully) extended without additional requirements on the fields [CS19a]. We will comment further on this in Sect. 4.2.

#### Remark 29

The main difficulty in constructing BFV data for PC theory comes from the requirement that $({\mathcal {F}}^\partial _{PC},\varpi ^\partial _{PC})$ be a symplectic manifold, as opposed to pre-symplectic (cf. with [AG14, Gri16]). We stress that this requirement is essential for quantisation. To the best of our knowledge, a complete description of the *symplectic* BFV structure for PC gravity was not available before [CCS20]. The complexity of the symplectic reduction arising in the classical description of the degree-0 boundary structure [CCS20, CS19c], as well as the obstruction in the BV–BFV induction procedure [CS19b], are relevant features peculiar to this formulation of gravity.

#### Remark 30

The conventions we choose for the fields in (20) and in (21) differ from those in [CS19a, Proposition 21]. In order to make contact between the formulas one has to perform the following change of variables:

$$\begin{aligned} e^{\dag } \mapsto y^{\dag }&\quad \omega ^{\dag } \mapsto c^{\dag } \\ c \mapsto - c&\quad \xi \mapsto -\xi \quad \xi ^{n} \mapsto - \lambda . \end{aligned}$$

In the case $N \ge 4$ some signs differ from the ones in [CCS20] due to a sign convention for $\lambda$.

## AKSZ EH

We explore here the idea of reconstructing the $(d+1)$-dimensional BV extension of Einstein–Hilbert theory by means of the AKSZ construction, with target the BFV data for Einstein–Hilbert theory (as presented in Sect. 2.1, based on [CS16]). In order to do this one looks at the space ${\mathcal {F}}^{\textsf {\tiny AKSZ}}_{EH}:=\mathrm {Maps}(T[1]I,{\mathcal {F}}_{EH}^\partial (\Sigma ))$, with *I* an interval. In a chart, to consider the transgression map of Eq. (13) means to look at fields composed of a 0-form and a 1-form on the interval *I*, with values in ${\mathcal {F}}_{EH}^\partial (\Sigma )$ and fixed total degree. For the case at hand we will then have

23a $$\begin{aligned} {\mathfrak {G}} = {\gamma }(t) + {\Pi }^\dag (t) dt&{\mathfrak {Z}}^n = \xi ^{n}(t) + \eta (t) dt&{\mathfrak {Z}}^a = \xi ^{a}(t) + \beta ^a(t) dt \end{aligned}$$

23b $$\begin{aligned} {\mathfrak {P}} = {\Pi }(t) + {\gamma }^\dag (t) dt&{\mathfrak {H}}_n = {\varphi }_n(t) + \xi ^\dag _n(t) dt&{\mathfrak {H}}_a = {\varphi }_a(t) + \xi ^\dag _a(t) dt \end{aligned}$$

where, for all $t\in I$, we parametrise ${\mathcal {F}}^{\textsf {\tiny AKSZ}}_{EH}$ with fields^19^

$$\begin{aligned} {\gamma }(t)\in \mathrm {Map}(I,S_{nd}^2(T\Sigma )), \quad&{\gamma }^\dag (t)\in \mathrm {Map}(I,S^2[-1](T^*\Sigma )), \\ {\Pi }(t)\in \mathrm {Map}(I,S^2(T^*\Sigma )), \quad&{\Pi }^\dag (t)\in \mathrm {Map}(I,S[-1]^2(T\Sigma )), \\ \eta (t), \beta ^a(t)\in \mathrm {Map}(I,C^\infty (\Sigma )), \quad&{\varphi }_n(t),{\varphi }_a(t)\in \mathrm {Map}(I,\mathrm {Dens}[-1](\Sigma )), \\ \xi ^n(t), \xi ^a(t)\in \mathrm {Map}(I,C^\infty [1](\Sigma )), \quad&\xi ^\dag _n(t),\xi ^\dag _a(t)\in \mathrm {Map}(I,\mathrm {Dens}[-2](\Sigma )), \end{aligned}$$

where we required $\gamma (t)$ to be nondegenerate for all $t\in I$. Now, observe that $\eta , \xi ^n$ are functions on $\Sigma$ whereas $\beta ^a, \xi ^a$ can be considered as the components of vector (fields) tangent to $\Sigma$, which we will denote by $\beta$ and $\xi ^\partial$. Similarly, we can promote ${\varphi }_a$ and $\xi ^\dag _a$ into $\Sigma$-density valued one forms, which we will denote by ${\varphi }_\partial , \xi ^\dag _\partial$. For simplicity of notation we will often use a unified index $\rho \in \{n,a\}$, so that ${\varphi }_\rho \in \{{\varphi }_n,{\varphi }_a\}$ and $\xi ^\dag _\rho \in \{\xi ^\dag _n,\xi ^\dag _a\}$.

### Remark 31

Notice once again that we are using (nondegenerate) sections of $S^2(TM)$ instead of actual metrics. Occasionally we will need to raise/lower indices using $\gamma$ and its “inverse” which we will denote by ${\gamma }^{-1}$. See Remark 20.

In what follows it will be useful to denote the *Kinetic* part of the Hamiltonian functional [Eq. (13)] as

24 $$\begin{aligned} {\mathcal {K}}:=\frac{1}{\sqrt{{\gamma }}}\left( \mathrm {Tr}_{{\gamma }}[{\Pi }^2] - \frac{1}{d-1}\mathrm {Tr}_{{\gamma }}[{\Pi }]^2\right) , \end{aligned}$$

and the *cosmological Einstein tensor* with respect to a metric ${\gamma }^{-1}$ with cosmological constant $\Lambda$ will be

25 $$\begin{aligned} {\mathbf {G}}[{\gamma },\Lambda ] = R[{\gamma }] + \left( \Lambda - \frac{1}{2} \mathrm {Tr}_{{\gamma }} R[{\gamma }]\right) {\gamma }^{-1}, \end{aligned}$$

where $R[{\gamma }]$ is the Ricci–Riemann tensor of ${\gamma }$. We also introduce a tensor-valued second order operator ${\mathbf {D}}_{{\gamma }}$ that on functions $\phi \in C^\infty (\Sigma )$ acts as

26 $$\begin{aligned} {\mathbf {D}}_{{\gamma }}\phi = \gamma ^{-1} \Delta ^\partial \phi - \nabla ^\partial \odot \nabla ^\partial \phi \end{aligned}$$

where $\nabla ^\partial$ denotes the Levi-Civita connection of ${\gamma }$, and we denoted by $\Delta ^\partial =\nabla ^\partial \cdot \nabla ^\partial$ the Laplace–Beltrami operator. In a coordinate chart this reads:

$$\begin{aligned}{}[{\mathbf {D}}_{{\gamma }}\phi ]_{ab}={{\gamma }}_{ab}{{\gamma }}^{cd}\nabla ^\partial _c\nabla ^\partial _d\phi - \nabla ^\partial _a\nabla ^\partial _b\phi . \end{aligned}$$

In what follows we will also use the metric gradient, i.e. the vector field $\mathrm {grad}_{\gamma }\phi = {\gamma }(d\phi ,\cdot )$. Since the covariant derivative $\nabla ^\partial$ will not explicitly appear in what follows, we shall employ the symbol $\nabla$ to introduce a shorthand notation for the metric gradient:

$$\begin{aligned} \nabla _{\gamma }\phi \equiv \frac{1}{2}\mathrm {grad}_{\gamma }\phi . \end{aligned}$$

Then, it is a matter of a straightforward calculation to show that

$$\begin{aligned} {\mathbf {D}}_{{\gamma }}\phi = \frac{1}{2} \left( \gamma ^{-1} \mathrm {Tr}[{\mathcal {L}}_{\nabla _{\gamma }\phi }\gamma ] - {\mathcal {L}}_{\nabla _{\gamma }\phi }\gamma ^{-1}\right) . \end{aligned}$$

With this in mind we can state the following:

### Theorem 32

The AKSZ data ${\mathfrak {F}}_{EH}^{\textsf {\tiny AKSZ}}(I;{\mathfrak {F}}^\partial _{EH}(\Sigma ))$ are given by the $(-1)$-shifted symplectic manifold

$$\begin{aligned} {\mathcal {F}}^{\textsf {\tiny AKSZ}}_{EH}&\simeq T^*[-1]\left( \mathrm {Map}(I,S_{nd}^2(T\Sigma ) \times S^2(T^*\Sigma )\times C^\infty (\Sigma )\times {\mathfrak {X}}(\Sigma )\right. \\&\left. \quad \times {\mathfrak {X}}[1](\Sigma )\times C^\infty [1](\Sigma ))\right) \end{aligned}$$

27 $$\begin{aligned} \Omega ^{\textsf {\tiny AKSZ}}_{EH} = \int \limits _{\Sigma \times I} \left\{ - \langle \delta {\gamma }, \delta {\gamma }^\dag \rangle + \langle \delta {\Pi }, \delta {\Pi }^\dag \rangle + \delta \xi ^{\rho }\delta \xi ^\dag _\rho + \delta \eta \delta {\varphi }_n + \delta \beta ^a\delta {\varphi }_a\right\} dt \end{aligned}$$

and the AKSZ action functional:

28a $$\begin{aligned} S^{\textsf {\tiny AKSZ}}_{EH}&= \int \limits _{\Sigma \times I} \Big \{\langle {\Pi }, {\dot{{\gamma }}}\rangle - \langle {\varphi }_\rho , {\dot{\xi }}^\rho \rangle +H_n \eta + H_\partial (\beta ) - \langle {\gamma }^\dag , L_{\xi ^{\partial }}{\gamma }\rangle + \langle {\Pi }^\dag , L_{\xi ^{\partial }}{\Pi }\rangle \end{aligned}$$

28b $$\begin{aligned}&\quad -\, \left( \frac{\delta {\mathcal {K}}}{\delta {\gamma }}({\Pi }^\dag ) + \frac{\delta {\mathcal {K}}}{\delta {\Pi }}({\gamma }^\dag )\right) \xi ^n - \sqrt{{\gamma }}\langle {\Pi }^\dag , {\mathbf {G}}[{\gamma },\lambda ] \xi ^n + {\mathbf {D}}_{{\gamma }}(\xi ^n) \rangle \end{aligned}$$

28c $$\begin{aligned}&\quad + \,\langle {\varphi }_\partial , \nabla _\gamma \eta \xi ^{n} - \eta \nabla _\gamma \xi ^{n} + L_{\xi ^\partial } \beta \rangle - {\varphi }_n L_{\beta }\xi ^n +{\varphi }_n L_{\xi ^\partial }\eta \end{aligned}$$

28d $$\begin{aligned}&\quad +\, \left\langle \xi ^\dag _\partial , \frac{1}{2} [\xi ^{\partial },\xi ^{\partial }] + \xi ^{n}\nabla _\gamma \xi ^{n} \right\rangle + {\Pi }^\dag ({\varphi }_\partial ,d\xi ^{n})\xi ^{n} + \xi _n^\dag L_{\xi ^{\partial }}\xi ^{n} \Big \} dt, \end{aligned}$$

together with its Hamiltonian vector field $Q^{\textsf {\tiny AKSZ}}_{EH}$.

### Proof

The prescription of Theorem 14, suggests that to construct the data in ${\mathfrak {F}}^{\textsf {\tiny AKSZ}}(I;{\mathfrak {F}}^\partial _{EH})$ we need to compute

29 $$\begin{aligned} \Omega ^{\textsf {\tiny AKSZ}}_{EH} = {\mathfrak {T}}_I^{(2)}\varpi _{EH}^\partial (\Sigma ) = \int \limits _{\Sigma \times I} \langle \delta {\mathfrak {P}}, \delta {\mathfrak {G}} \rangle + \langle \delta {\mathfrak {H}}_\rho , \delta {\mathfrak {Z}}^\rho \rangle . \end{aligned}$$

By selecting the top-form part of the integrand and observing that $|dt|=1$ we get

30 $$\begin{aligned} \Omega ^{\textsf {\tiny AKSZ}}_{EH}= \int \limits _{\Sigma \times I} \left\{ - \langle \delta {\gamma }^\dag , \delta {\gamma }\rangle + \langle \delta {\Pi }, \delta {\Pi }^\dag \rangle + \delta \xi ^\dag _\rho \delta \xi ^{\rho } + \delta {\varphi }_n \delta \eta + \delta {\varphi }_a\delta \beta ^a \right\} dt \end{aligned}$$

where the sign comes from $\langle \delta ({\gamma }^\dag dt), \delta {\gamma }\rangle = - \langle \delta {\gamma }^\dag , \delta {\gamma }\rangle dt$, since $|\delta {\gamma }|=1$, while $\delta \xi ^\dag _\rho dt \delta \xi ^\rho = \delta \xi ^\dag _\rho \delta \xi ^\rho dt$, since $|\delta \xi ^\rho |=2$. $\Omega ^{\textsf {\tiny AKSZ}}$ is a $(-1)$-symplectic structure on $\mathrm {Maps}(T[1]I,{\mathcal {F}}_{EH}^\partial (\Sigma ))$, a BV 2-form.

Now, from $\alpha _{EH}^\partial (\Sigma )$ we can construct a degree-0 functional on ${\mathcal {F}}^{\textsf {\tiny AKSZ}}_{EH}$ by first applying the transgression map, which yields the 1-form

$$\begin{aligned} {\mathfrak {T}}^{(1)}_I\alpha ^\partial \in \Omega ^1(\mathrm {Maps}(T[1]I,{\mathcal {F}}_{EH}^\partial (\Sigma ))), \end{aligned}$$

and then contracting it with the de Rham differential on *I* seen as an odd cohomological vector field $d_I$. In a local chart this is tantamount to replacing $\delta \leadsto d_I:= dt\frac{d}{dt}$, so that

31 $$\begin{aligned} \iota _{d_I}{\mathfrak {T}}^{(1)}_I\alpha _{EH}^\partial (\Sigma ) = \int \limits _{\Sigma \times I} \left\{ \langle {\Pi }, {\dot{{\gamma }}}\rangle - \langle {\varphi }_\rho , {\dot{\xi }}^\rho \rangle \right\} dt. \end{aligned}$$

where the sign comes from the fact that $\langle {{\varphi }_\rho } dt,\,{\dot{\xi }}^\rho \rangle = -\langle {{\varphi }_\rho }, {\dot{\xi }}^\rho \rangle dt$. Finally, we want to compute the AKSZ action functional

$$\begin{aligned} S^{\textsf {\tiny AKSZ}}_{EH}:={\mathfrak {T}}_I^{(0)}(S_{EH}^\partial (\Sigma )) + \iota _{d_{I}}{\mathfrak {T}}_I^{(1)}(\alpha _{EH}^\partial (\Sigma )). \end{aligned}$$

This calculation is completely analogous to the previous ones, and it is mostly straightforward. One needs to pay attention to the signs, so it is worthwhile to stress that

$$\begin{aligned} {\Pi }^\dag dt\, ({\varphi }_\partial , d\xi ^n) \xi ^n = [{\Pi }^\dag ]^{ab} dt {\varphi }_a \partial _b \xi ^n \xi ^n = [{\Pi }^\dag ]^{ab} {\varphi }_a \partial _b \xi ^n \xi ^n dt = {\Pi }^\dag ({\varphi }_\partial , d\xi ^n) \xi ^n dt \end{aligned}$$

while

$$\begin{aligned}&-{\gamma }(\xi ^\dag _\partial dt, d\xi ^n)\xi ^n - {\gamma }({\varphi }_\partial , d \eta \, dt\,\xi ^n + d \xi ^n\eta dt) \\&\quad = \left( \langle \xi ^\dag _\partial , \xi ^n \nabla _{{\gamma }}\xi ^n\rangle + \langle {\varphi }_\partial , \nabla _{{\gamma }}\eta \xi ^n - \eta \nabla _{{\gamma }}\xi ^n\rangle \right) dt \end{aligned}$$

Finally, at first order in *dt*,

$$\begin{aligned} H_n({\gamma }+ {\Pi }^\dag dt, {\Pi }+ {\gamma }^\dag ) \xi ^n = \frac{\delta (H_n\xi ^n)}{\delta {\gamma }}({\Pi }^\dag dt) + \frac{\delta (H_n \xi ^n)}{\delta {\Pi }}({\gamma }^\dag dt ), \end{aligned}$$

and $dt\, \xi ^n = - \xi ^n dt$. We write the formulas above as derivatives of the functional $H_n\xi ^n$ to stress that total derivatives will appear, due to the term $R^\partial$ in $H_n$. Recalling the expression for $H_n$ of Eq. (13) and the definition of ${\mathcal {K}}$, ${\mathbf {G}}[\gamma ,\Lambda ]$ and ${\mathbf {D}}_\gamma$ of Eqs. (24)–(26), the variation of $H_n \xi ^n$ with respect to ${\gamma }$ yields

$$\begin{aligned} \frac{\delta (H_n\xi ^n)}{\delta {\gamma }} = \frac{\delta {\mathcal {K}}}{\delta {\gamma }}\xi ^n + \frac{\delta (\sqrt{{\gamma }}R^\partial \xi ^n)}{\delta {\gamma }} = \frac{\delta {\mathcal {K}}}{\delta {\gamma }}\xi ^n + \sqrt{{\gamma }}\left( {\mathbf {G}}[{\gamma },\Lambda ] \xi ^n + {\mathbf {D}}_{{\gamma }}(\xi ^n)\right) + d(\dots ). \end{aligned}$$

The total derivative term is exact with respect to the tangent differential $d_\Sigma$. It can be discarded, provided $\Sigma$ has no boundary (which we are assuming throughout), so:

32 $$\begin{aligned} H_n({\gamma }+ {\Pi }^\dag dt, {\Pi }+ {\gamma }^\dag ) \xi ^n= & {} -\left( \frac{\delta {\mathcal {K}}}{\delta {\Pi }}({\gamma }^\dag ) + \frac{\delta {\mathcal {K}}}{\delta {\gamma }}({\Pi }^\dag )\right) \xi ^n dt - \sqrt{{\gamma }}\left\langle {\Pi }^\dag ,{\mathbf {G}}[{\gamma },\Lambda ] \xi ^n \right. \nonumber \\&\left. +\, {\mathbf {D}}_{{\gamma }}(\xi ^n)\right\rangle dt. \end{aligned}$$

$$\square $$

### Remark 33

In order to compute the cohomological vector field $Q^{\textsf {\tiny AKSZ}}_{EH}$ we enforce the Hamiltonian condition $\iota _{Q^{\textsf {\tiny AKSZ}}_{EH}} \Omega ^{\textsf {\tiny AKSZ}}_{EH}= \delta S^{\textsf {\tiny AKSZ}}_{EH}$ dropping all possible boundary terms. It reads (we omit the expression for $Q^{\textsf {\tiny AKSZ}}_{EH}\xi ^\dag$ and $Q^{\textsf {\tiny AKSZ}}_{EH}{\varphi }$):

33a $$\begin{aligned} Q^{\textsf {\tiny AKSZ}}_{EH} {\gamma }&= \frac{\delta H_n}{\delta {\Pi }}\xi ^n + L_{\xi ^\partial }\gamma \end{aligned}$$

33b $$\begin{aligned} Q^{\textsf {\tiny AKSZ}}_{EH} {\Pi }&= - \frac{\delta {\mathcal {K}}}{\delta {\gamma }}\xi ^n - \sqrt{{\gamma }}\left( {\mathbf {G}}[{\gamma },\Lambda ] \xi ^n + {\mathbf {D}}_{{\gamma }}(\xi ^n)\right) + L_{\xi ^\partial }{\Pi }{ + } {\varphi }\odot d\xi ^n \xi ^n \end{aligned}$$

33c $$\begin{aligned} Q^{\textsf {\tiny AKSZ}}_{EH} \eta&= - {\dot{\xi }}{}^n + L_{\xi ^{\partial }}\eta - L_\beta \xi ^n \end{aligned}$$

33d $$\begin{aligned} Q^{\textsf {\tiny AKSZ}}_{EH} \beta&= - {\dot{\xi }}{}^\partial + L_{\xi ^\partial }\beta + \nabla _{{\gamma }} \eta \,\xi ^n - \eta \, \nabla _{{\gamma }}\xi ^n - \nabla _{{\Pi }^\dag }\xi ^n \xi ^n \end{aligned}$$

33e $$\begin{aligned} Q^{\textsf {\tiny AKSZ}}_{EH} \xi ^\partial&= \frac{1}{2}[\xi ^\partial ,\xi ^\partial ] + \xi ^n\nabla _{{\gamma }}\xi ^n \end{aligned}$$

33f $$\begin{aligned} Q^{\textsf {\tiny AKSZ}}_{EH} \xi ^n&= L_{\xi ^\partial }\xi ^n \end{aligned}$$

33g $$\begin{aligned} Q^{\textsf {\tiny AKSZ}}_{EH}{\gamma }^\dag&= {\dot{{\Pi }}} + \frac{\delta {\mathcal {K}}}{\delta {\gamma }}\eta + \sqrt{{\gamma }}\left( {\mathbf {G}}[{\gamma },\Lambda ] \eta + {\mathbf {D}}_{{\gamma }}(\eta )\right) + L_\beta {\Pi }+ L_{\xi ^\partial } {\gamma }^\dag \end{aligned}$$

33h $$\begin{aligned}&\quad +\, \, \xi ^\dag _\partial \odot d\xi ^n\xi ^n -{\varphi }_\partial \odot d\eta \xi ^n { +} \eta {\varphi }_\partial \odot d\xi ^n \end{aligned}$$

33i $$\begin{aligned}&\quad +\, \, \left[ \frac{\delta ^2{\mathcal {K}}}{\delta {\gamma }^2}({\Pi }^\dag ) + \frac{\delta ^2H_n}{\delta {\gamma }\delta {\Pi }}({\gamma }^\dag )\right] \xi ^n - \frac{1}{2}{\gamma }^{-1} \langle {\Pi }^\dag , {\mathbf {G}}[{\gamma },\lambda ] \xi ^n + {\mathbf {D}}_{{\gamma }}(\xi ^n) \rangle \end{aligned}$$

33j $$\begin{aligned}&\quad -\, \sqrt{{\gamma }}\left\langle {\Pi }^\dag , \frac{\delta {\mathbf {G}}[{\gamma },\lambda ]}{\delta {\gamma }} \xi ^n + \frac{\delta {\mathbf {D}}_{{\gamma }}(\xi ^n)}{\delta {\gamma }}\right\rangle \end{aligned}$$

33k $$\begin{aligned} Q^{\textsf {\tiny AKSZ}}_{EH}{\Pi }^\dag&={\dot{{\gamma }}} + \frac{\delta {\mathcal {K}}}{\delta {\Pi }}\eta + L_\beta {\gamma }+ L_{\xi ^\partial } {\Pi }^\dag { -} \left[ \frac{\delta ^2{\mathcal {K}}}{\delta {\Pi }^2}({\gamma }^\dag ) + \frac{\delta ^2{\mathcal {K}}}{\delta {\gamma }\delta {\Pi }}({\Pi }^\dag )\right] \xi ^n. \end{aligned}$$

### Remark 34

We notice that the term $\sqrt{{\gamma }}{\mathbf {D}}_{{\gamma }}(\cdot )$ is the contribution to the field equations for a metric due to the presence of a Brans–Dicke “dilaton” field, whose role is played by the ghost $\xi ^n$ in the BFV action $S_{EH}^\partial (\Sigma )$ and by $\eta$ in $S^{\textsf {\tiny AKSZ}}_{EH}$.

### Pushforward

We would like to compute the BV pushforward of ${\mathfrak {F}}^{\textsf {\tiny AKSZ}}$ along the symplectic submanifold $({\Pi },{\Pi }^\dag )\in {\mathcal {F}}''=T^*[-1]\mathrm {Map}(I,S^2(T^*\Sigma ))\subset {\mathcal {F}}^{\textsf {\tiny AKSZ}}_{EH}$.

#### Remark 35

This is the same as evaluating $S_{\text {eff}}$ from Eq. (3). Since $S^{\textsf {\tiny AKSZ}}_{EH}$ is only quadratic in $\Pi$, the calculation reduces to computing the Batalin–Vilkovisky–Legendre transform $S_{\text {BVL}}$ of $S^{\textsf {\tiny AKSZ}}_{EH}$ with respect to ${\mathcal {L}}$, as in Definition 11, plus a correction in the integration measure for the remaining (second-order) effective theory. Note that Eq. 2 is equivalent to setting to zero the r.h.s. of Eq. (33k), together with $\Pi ^\dag =0$.

Recall that we are assuming $\gamma (t)$ to be a nondegenerate section of $S^2(T\Sigma )$ for every *t*, i.e. it represents the inverse of a metric, and dually ${\Pi }(t)\in S^2(T^*\Sigma )$. We can use ${\gamma }$ and its inverse (denoted ${\gamma }^\flat$) to raise/lower indices: explicitly, if ${\gamma }=\gamma ^{ab}\partial _a\odot \partial _b$, we have ${\gamma }^\flat =\gamma _{ab}dx^a\odot dx^b$, with $\gamma ^{ab}\gamma _{bc}=\delta ^a_c$. Then, for $X\in S^2(T^*\Sigma ),\ Y\in S^2(T\Sigma )$ we define $(X^\sharp )^{ab} := {\gamma }^{ac}{\gamma }^{bd}X_{bc}$ and $(Y^\flat )_{ab} = {\gamma }_{ac}{\gamma }_{bd}Y^{cd}$.

#### Definition 36

Consider the space of fields ${{\mathcal {F}}}_{R}(\Sigma \times I){}\subseteq {\mathcal {F}}^{\textsf {\tiny AKSZ}}$ as

34 $$\begin{aligned} {{\mathcal {F}}}_{R}(\Sigma \times I){} :=T^*[-1]\left( \mathrm {Map}\left( I,S_{nd}^2(T\Sigma ) \times T[1]\left( C^\infty (\Sigma )\times {\mathfrak {X}}(\Sigma )\right) \right) \right) \end{aligned}$$

parametrised by $(\gamma ,\eta ,\beta ,\xi ^n,\xi ^\partial ,\varphi _n,\varphi _\partial ,\xi ^\dag _n,\xi ^\dag _\partial )$, with $\iota _{EH}{:}\,{{\mathcal {F}}}_{R}(\Sigma \times I){}\rightarrow {\mathcal {F}}^{AKSZ}$ the inclusion map.

#### Theorem 37

The BV-pushforward of ${\mathfrak {F}}^{\textsf {\tiny AKSZ}}(I;{\mathfrak {F}}^\partial _{EH}(\Sigma ))$ with respect to the Lagrangian submanifold ${\mathcal {L}}=\{({\Pi },{\Pi }^\dag )\in {\mathcal {F}}''\ |\ {\Pi }^\dag =0\}$ is the BV theory given by

$$\begin{aligned} {{\mathfrak {F}}}_{R}(\Sigma \times I):=({{\mathcal {F}}}_{R}(\Sigma \times I),{S}_{R}(\Sigma \times I){}, {\varpi }_{R}(\Sigma \times I){}) \end{aligned}$$

where

35 $$\begin{aligned} {S}_{R}(\Sigma \times I){}&= \int \limits _{{\mathbb {R}}}dt\int \limits _{\Sigma } -\eta \sqrt{{\gamma }}\left[ \left( \langle K^\sharp , K\rangle - \mathrm {Tr}(K)^2\right) + R^\partial - 2\Lambda \right] \nonumber \\&\quad -\,\langle {\gamma }^\dag , L_{\xi ^\partial }{\gamma }\rangle - 2\langle K^\sharp , {\gamma }^\dag \rangle \xi ^n -\langle {\varphi }_\rho , {\dot{\xi }}^\rho \rangle \nonumber \\&\quad +\, \, \langle {\varphi }_\partial , \nabla _\gamma \eta \xi ^{n} - \eta \nabla _\gamma \xi ^{n} + L_{\xi ^\partial } \beta \rangle +{\varphi }_n \left( - L_{\beta }\xi ^n +L_{\xi ^\partial }\eta \right) \nonumber \\&\quad +\, \, \left\langle \xi ^\dag _\partial , \xi ^{n}\nabla _\gamma \xi ^{n} + \frac{1}{2} [\xi ^{\partial },\xi ^{\partial }] \right\rangle + \xi _n^\dag L_{\xi ^{\partial }}\xi ^{n} \end{aligned}$$

with $$K := \frac{\eta ^{-1}}{2} \left( {\dot{{\gamma }}} + L_\beta {\gamma }\right) ^\flat $$, and

36 $$\begin{aligned} {\varpi }_{R}(\Sigma \times I){}=\iota _{EH}^*\Omega ^{\textsf {\tiny AKSZ}}_{EH}. \end{aligned}$$

#### Proof

As discussed in Remark 35, we are interested in finding the effective action one obtains by means of the perturbative expansion of the integral

37 $$\begin{aligned} \exp \left( \frac{i}{\hbar }S_{\text {eff}}\right) :=\int \limits _{{\mathcal {L}}\subset {\mathcal {F}}''} \exp \left( \frac{i}{\hbar }S^{\textsf {\tiny AKSZ}}_{EH}\right) \end{aligned}$$

because $S^{\textsf {\tiny AKSZ}}_{EH}\vert _{{\mathcal {L}}}$ is quadratic in $\Pi$, through the term ${\mathcal {K}}(\Pi )\eta$. Observing that

38 $$\begin{aligned} \frac{\delta {\mathcal {K}}}{\delta {\Pi }}&= \frac{2}{\sqrt{{\gamma }}}\left( {\Pi }^\sharp - \frac{{\gamma }}{d-1} \mathrm {Tr}({\Pi })\right) \end{aligned}$$

39 $$\begin{aligned} \frac{\delta ^2 {\mathcal {K}}}{\delta {\Pi }^2}({\gamma }^\dag )&= \frac{2}{\sqrt{{\gamma }}}\left( ({\gamma }^\dag )^\sharp - \frac{{\gamma }}{d-1} \mathrm {Tr}({\gamma }^\dag )\right) , \end{aligned}$$

we have that Eq. (33k) reads

40 $$\begin{aligned}&\frac{2}{\sqrt{{\gamma }}}\left( {\Pi }^\sharp - \frac{{\gamma }}{d-1} \mathrm {Tr}({\Pi })\right) \nonumber \\&\quad = -\eta ^{-1}\left( {\dot{{\gamma }}} + L_\beta {\gamma }{ -} \frac{2}{\sqrt{{\gamma }}}\left( ({\gamma }^\dag )^\sharp - \frac{{\gamma }}{d-1} \mathrm {Tr}({\gamma }^\dag )\right) \xi ^n\right) + F({\Pi }^\dag ) \end{aligned}$$

where $\mathrm {Tr}(X) = {\gamma }^{ab}X_{ab}$. We will use the symbol $\approx$ to denote the enforcing of Eq. (33k) and of ${\Pi }^\dag =0$. Then, requiring ${\Pi }^\dag =0$ and defining

41 $$\begin{aligned} K := \frac{\eta ^{-1}}{2} \left( {\dot{{\gamma }}} + L_\beta {\gamma }\right) ^\flat , \end{aligned}$$

we obtain that

42 $$\begin{aligned} {\Pi }\approx - \sqrt{{\gamma }}\left( K - \mathrm {Tr}(K) {\gamma }^\flat \right) + \eta ^{-1} {\gamma }^\dag \xi ^n. \end{aligned}$$

It is easy to compute now

43 $$\begin{aligned} H_n \approx \sqrt{{\gamma }}\left[ \left( \langle K^\sharp , K\rangle - \mathrm {Tr}(K)^2\right) + R^\partial - 2\Lambda \right] - 2\eta ^{-1}\langle K^\sharp , {\gamma }^\dag \rangle \xi ^n \end{aligned}$$

which, together with

44 $$\begin{aligned} \langle {\Pi }, {\dot{{\gamma }}} + L_\beta {\gamma }\rangle \approx -2 \sqrt{{\gamma }}\left[ \left( \langle K^\sharp , K\rangle - \mathrm {Tr}(K)^2\right) + R^\partial - 2\Lambda \right] + 2\eta ^{-1}\langle K^\sharp , {\gamma }^\dag \rangle \xi ^n \end{aligned}$$

and

45 $$\begin{aligned} -\frac{\delta H_n}{\delta {\Pi }}({\gamma }^\dag )\xi ^n \approx + 2\langle K^\sharp , {\gamma }^\dag \rangle \xi ^n, \end{aligned}$$

yields

46 $$\begin{aligned} S^{\textsf {\tiny AKSZ}}_{EH}&\approx \int \limits _{{\mathbb {R}}}dt\int \limits _{\Sigma } -\eta \sqrt{{\gamma }}\left[ \left( \langle K^\sharp , K\rangle - \mathrm {Tr}(K)^2\right) + R^\partial - 2\Lambda \right] \nonumber \\&\quad -\,\langle {\gamma }^\dag , L_{\xi ^\partial }{\gamma }\rangle + 2\langle K^\sharp , {\gamma }^\dag \rangle \xi ^n -\langle {\varphi }_\rho , {\dot{\xi }}^\rho \rangle \nonumber \\&\quad +\, \, \langle {\varphi }_\partial , \nabla _\gamma \eta \xi ^{n} - \eta \nabla _\gamma \xi ^{n} + L_{\xi ^\partial } \beta \rangle +{\varphi }_n \left( - L_{\beta }\xi ^n +L_{\xi ^\partial }\eta \right) \nonumber \\&\quad +\, \, \left\langle \xi ^\dag _\partial , \xi ^{n}\nabla _\gamma \xi ^{n} + \frac{1}{2} [\xi ^{\partial },\xi ^{\partial }] \right\rangle + \xi _n^\dag L_{\xi ^{\partial }}\xi ^{n} =:{S}_{R}(\Sigma \times I){}. \end{aligned}$$

So, formula (46) shows that $S_{\text {eff}} = {S}_{R}(\Sigma \times I){} + O(\hbar )$. The $\hbar$ correction is the (logarithm of the) determinant of the operator defining the quadratic form ${\mathcal {K}}$, i.e. the determinant of the deWitt super metric^20^ [DeW67]

$$\begin{aligned} {\mathsf {W}}_\gamma ^{ijkl} = \frac{1}{\sqrt{\gamma }}\left( \gamma ^{ik}\gamma ^{jl} - \frac{1}{d-1} \gamma ^{ij}\gamma ^{kl}\right) , \end{aligned}$$

or, more invariantly

47 $$\begin{aligned} {\mathsf {W}}_\gamma (\Pi ,\Pi ) = \frac{1}{\sqrt{\gamma }} \left( \langle \Pi ^\sharp ,\Pi \rangle - \frac{1}{d-1}\mathrm {Tr}_\gamma [\Pi ]^2\right) , \end{aligned}$$

and it will have the effect of correcting the overall measure on the residual BV space of fields ${{\mathcal {F}}}_{R}(\Sigma \times I){}$. $\square$

#### Remark 38

Up to boundary, we can compute ${Q}_{R}(\Sigma \times I){}$ (denoted hereinafter by ${Q}_R$) to be:

48a $$\begin{aligned} {Q}_R\gamma&= L_{\xi ^\partial }\gamma - \eta ^{-1}({\dot{\gamma }} + L_\beta \gamma ) \xi ^n \end{aligned}$$

48b $$\begin{aligned} {Q}_R\eta&= - L_{\beta }\xi ^n +L_{\xi ^\partial }\eta \end{aligned}$$

48c $$\begin{aligned} {Q}_R\beta&= \nabla _{\gamma }\eta \xi ^{n} - \eta \nabla _{\gamma }\xi ^{n} + L_{\xi ^\partial } \beta \end{aligned}$$

48d $$\begin{aligned} {Q}_R\xi ^n&= L_{\xi ^{\partial }}\xi ^{n} \end{aligned}$$

48e $$\begin{aligned} {Q}_R\xi ^\partial&= \xi ^{n}\nabla _{\gamma }\xi ^{n} + \frac{1}{2} [\xi ^{\partial },\xi ^{\partial }] \end{aligned}$$

and similarly for antifields.

### Reconstruction of Einstein–Hilbert theory

In this section we wish to show that the BV pushforward of the AKSZ theory constructed in Sect. 3.1 is strongly equivalent to Einstein–Hilbert theory in the BV formalism.

To do this, we begin by considering the following definitions:

49a $$\begin{aligned} {\widetilde{\xi }}^{}&= -\eta ^{-1}\xi ^n (\partial _t + \beta ) + \xi ^\partial \end{aligned}$$

49b $$\begin{aligned} {\widetilde{g}}&= -\eta ^{-2} \partial _t\odot \partial _t - 2 \eta ^{-2} \beta \odot \partial _t+ {\gamma }- \eta ^{-2} \beta \odot \beta \end{aligned}$$

#### Lemma 39

We have the following relations

50a $$\begin{aligned} \frac{1}{2}[{\widetilde{\xi }}^{},{\widetilde{\xi }}^{}]&= {Q}_R{\widetilde{\xi }}^{}, \end{aligned}$$

50b $$\begin{aligned} L_{{\widetilde{\xi }}^{}}{\widetilde{g}}&= {Q}_R{\widetilde{g}}. \end{aligned}$$

#### Proof

It is a straightforward calculation to show

$$\begin{aligned} {Q}_R{\widetilde{\xi }}^{}&= \eta ^{-2} ({Q}_R\eta ) \xi ^{n}(\partial _t + \beta ) + \eta ^{-1}{Q}_R\xi ^n (\partial _t + \beta ) + {Q}_R\xi ^\partial + \eta ^{-1}\xi ^n {Q}_R\beta \\&= \left( - \eta ^{-2} {\dot{\xi }}{}^n \xi ^{n} - \eta ^{-2}L_\beta \xi ^{n} \xi ^{n} + L_{\xi ^\partial } (-\eta ^{-1} \xi ^{n}) \right) (\partial _t + \beta ) \\&\quad -\, \eta ^{-1}\xi ^n {\dot{\xi }}{}^\partial + \eta ^{-1}\xi ^n L_{\xi ^\partial }\beta + \frac{1}{2}[\xi ^\partial ,\xi ^\partial ]. \end{aligned}$$

Observe that the “algebroid term” (see Remark 40, below) $\xi ^n\nabla _{{\gamma }}\xi ^n$ in ${Q}_R{\xi ^\partial }$ cancels out with part of $\eta ^{-1}\xi ^n {Q}_R\beta$. On the other hand this coincides with

$$\begin{aligned} \frac{1}{2}[{\widetilde{\xi }}^{},{\widetilde{\xi }}^{}]&= \frac{1}{2} [ -\eta ^{-1}\xi ^n(\partial _t + \beta ) + \xi ^\partial , - \eta ^{-1}\xi ^n(\partial _t + \beta ) + \xi ^\partial ] \\&= \left( \eta ^{-2}\xi ^n(\partial _t + \beta )\xi ^n + L_{\xi ^\partial } (-\eta ^{-1}\xi ^n)\right) (\partial _t + \beta )\\&\quad -\, \eta ^{-1}\xi ^n{\dot{\xi }}{}^\partial +\eta ^{-1}\xi ^nL_{\xi ^\partial }\beta + \frac{1}{2}[\xi ^\partial ,\xi ^\partial ], \end{aligned}$$

proving the first claim. We compute

$$\begin{aligned} L_{{\widetilde{\xi }}^{}}{\widetilde{g}}&= - 2\eta ^{-3}{\dot{\xi }}{}^n\partial _t\partial _t + 2\eta ^{-3}L_{\xi ^\partial }\eta \partial _t\partial _t + 2\eta ^{-2}{\dot{\xi }}{}\partial _t - 2\eta ^{-4}\xi ^nL_\beta \eta \partial _t\partial _t \\&\quad -\, 2\eta ^{-2}\partial _t\left( \eta ^{-1}\xi ^n\beta \right) \partial _t -4\eta ^{-4}\xi ^n\partial _t\eta \beta \partial _t + 4\eta ^{-3} L_{\xi ^\partial }\eta \beta \partial _t - 4\eta ^{-4} L_\beta \eta \xi ^n \beta \partial _t \\&\quad -\, 2\eta ^{-2} L_{\xi ^\partial }\beta \partial _t - 2\eta ^{-2}L_\beta \left( \eta ^{-1}\xi ^n\right) \partial _t\partial _t + 2\eta ^{-2}\partial _t\left( \xi ^\partial - \eta ^{-1}\xi ^n\beta \right) \beta \\&\quad -\, 2\eta ^{-2}L_\beta \left( \eta ^{-1}{\xi ^n}{}\right) \beta \partial _t - 2\eta ^{-2}\partial _t\left( \eta ^{-1}{\xi ^n}{}\right) \beta \partial _t + 2\eta ^{-2}\xi ^n {\dot{\beta }}\partial _t - \eta ^{-1}{\xi ^n}{}{{\dot{\gamma }}}^{ab}\partial _a\partial _b \\&\quad +\, L_{\xi ^\partial }\left( \gamma ^{ab}\partial _a\partial _b\right) - \eta ^{-1}{\xi ^n}{} L_\beta (\gamma ^{ab})\partial _a\partial _b + 2\nabla _{{\gamma }}\left( \eta ^{-1}{\xi ^n}{}\right) \partial _t + 2\nabla _{{\gamma }}\left( \eta ^{-1}{\xi ^n\beta }{}\right) \partial _c \\&\quad -2\eta ^{-4}\xi ^n {{\dot{\eta }}} \beta \beta + 2\eta ^{-3}L_{\xi }\eta \beta \beta - 2\eta ^{-4}\xi ^nL_\beta \eta \beta \beta + 2\eta ^{-3} \xi ^n{{\dot{\beta }}}\beta -\eta ^{-2} L_{\xi ^\partial }\left( \beta \beta \right) \\&\quad -\, 2\eta ^{-2}L_\beta \left( \eta ^{-1}{\xi ^n}{}\right) (\beta \partial _t +\beta \beta ) \end{aligned}$$

where we recall that expressions like $L_{\xi ^\partial }(\beta )$ denote the Lie derivative of the vector field $\beta =\beta ^a\partial _a$ along $\xi ^\partial$. On the other hand we have

$$\begin{aligned} {Q}_R(-\eta ^{-2})\partial _t\partial _t&= \left( - 2 \eta ^{-3} {\dot{\xi }}{}^n + 2 \eta ^{-3}L_{\xi ^\partial }\eta - 2\eta ^{-3}L_\beta \xi ^n\right) \partial _t\partial _t\\ {Q}_R(-2\eta ^{-2}\beta )\partial _t&= \left( -4\eta ^{-3}{\dot{\xi }}{}^n\beta + 4\eta ^{-3} L_{\xi ^\partial }\eta - 4\eta ^{-3} L_\beta \xi ^n\beta \right) \partial _t \\&\quad +\, \, \left( 2\eta ^{-2}{\dot{\xi }}{}^\partial - 2\eta ^{-2} L_{\xi ^\partial }\beta - 2\eta ^{-2}\nabla _{{\gamma }}\eta \xi ^n + 2\eta ^{-1} \nabla _{{\gamma }}\xi ^n\right) \partial _t\\ {Q}_R(\gamma ^{ab})\partial _a\partial _b&= -\eta ^{-1}\xi ^n {\dot{{\gamma }}} - \eta ^{-1}\xi ^nL_\beta {\gamma }+ L_{\xi ^\partial }{\gamma }\\ {Q}_R(-\eta ^{-2}\beta \beta )&= - 2\eta ^{-3}{\dot{\xi }}{}^n \beta \beta + 2\eta ^{-3} L_{\xi ^\partial }\eta \beta \beta - 2\eta ^{-3}L_\beta \xi ^n\beta \beta \\&\quad +\, \, 2\eta ^{-2}\beta {\dot{\xi }}^\partial - 2\eta ^{-2} L_{\xi ^\partial }\beta \beta - 2\eta ^{-2}\left( \nabla _{{\gamma }}\eta \xi ^n - \eta \nabla _{{\gamma }}\xi ^n\right) \beta \end{aligned}$$

And it is a matter of a straightforward, but lengthy computation to show that the two expressions coincide. Indeed, subtracting one from the other we obtain

51 $$\begin{aligned}&L_{{\widetilde{\xi }}^{}}{\widetilde{g}}- {Q}_R{\widetilde{g}}\nonumber \\&\quad = 2 \eta ^{-3}(-\eta ^{-1} \xi ^n ( {\dot{\eta }} + L_\beta (\eta )) \beta \beta + 2\eta ^{-3}\xi ^n{\dot{\beta }}\beta - 2\eta ^{-2}L_\beta (\eta ^{-1})\xi ^n (\beta \partial _t + \beta \beta ) \nonumber \\&\ \qquad -\, 2\eta ^{-4}\xi ^n L_\beta (\eta ) \partial _t^2 + 2\eta ^{-4}{\dot{\eta }}\xi ^n \beta \partial _t - 2\eta ^{-3}\xi ^n {\dot{\beta }}\xi ^n \partial _t - 4\eta ^{-4}\xi ^n ({\dot{\eta }} + L_\beta \eta ) \beta \partial _t\nonumber \\&\ \qquad +\, \, 2\eta ^{-3}\xi ^n {\dot{\beta }} \partial _t + 2\eta ^{-4} L_\beta \eta (\partial _t^2 + \beta \partial _t) + 2\eta ^{-4}{\dot{\eta }}\xi ^n(\beta \partial _t + \beta \beta ) - 2\eta ^{-3}\xi ^n{\dot{\beta }}\beta \equiv 0 \end{aligned}$$

$$\square $$

#### Remark 40

Using Lemma 39 we wish to interpret (49) as a map of Lie algebroids. Consider the (trivial) vector bundle over

$$\begin{aligned} \mathrm {Map}(I,S_{nd}^2(T\Sigma ) \times C^\infty (\Sigma )\times {\mathfrak {X}}(\Sigma ))\simeq \mathcal {PR}^\Sigma (\Sigma \times I), \end{aligned}$$

where $\mathcal {PR}^{\Sigma }(\Sigma \times I)$ denotes pseudo-Riemennian metrics on $\Sigma \times I$ such that their restriction to $\Sigma$ is nondegenerate, with fibre

$$\begin{aligned} \mathrm {Map}(I,C^\infty (\Sigma ) \times {\mathfrak {X}}(\Sigma ))\simeq {\mathfrak {X}}(\Sigma \times I). \end{aligned}$$

We consider two different Lie algebroid structures on this vector bundle. One is the action algebroid with bracket given by the bracket of $(d+1)$-vector fields, and anchor given by Lie derivatives on metrics. The other algebroid structure is given by formulas (38), with (48a)–(48c) defining the anchor map, and (48d) and (48e) specifying the bracket of sections. Observe that the morphism of algebroids (49) does not preserve constant sections, as the splitting of a generic vector field ${\widetilde{\xi }}$ depends on the so-called *lapse* $\eta$ and *shift* $\beta$, which are coordinates on the base of the fibre bundle. The latter algebroid encodes the algebraic relations of the constraints of Einstein–Hilbert theory,^21^ and was carefully studied by other means in [BFW13]. It was also mentioned as a motivating example for the notion of Hamiltonian Lie Algebroid, introduced in [BW18]. It is an interesting question to check whether this construction satisfies the Hamiltonian requirements for an algebroid.

To proceed, we need to recall the BV data associated with Einstein–Hilbert theory, in the ADM formalism. Given a pseudo-Riemannian (inverse) metric ${\widetilde{g}}$ on a manifold *M*, we can perform a $d+1$ decomposition and rewrite it as^22^

$$\begin{aligned} \begin{array}{c} {\widetilde{g}}^{\mu \nu }=\left( \begin{array}{cc} -\eta ^{-2} &{}\quad -\eta ^{-2}\beta ^b \\ -\eta ^{-2}\beta ^a &{}\quad \gamma ^{ab}-\eta ^{-2}\beta ^a\beta ^b\end{array}\right) \end{array} \end{aligned}$$

In the case where *M* has a boundary, we can define the second fundamental form of the boundary submanifold $K_{ab}$ and its trace *K* by means of the boundary covariant derivative $\nabla ^\partial$ (the Levi-Civita connection of $\gamma$) as follows

52 $$\begin{aligned} K_{ab}=\frac{1}{2} \eta ^{-1}(2\nabla ^\partial _{(a}\beta _{b)} + \partial _t\gamma _{ab}) \quad K=\gamma ^{ab}K_{ab} \end{aligned}$$

where *t* denotes a coordinate transverse to the boundary $\partial M$. Finally, notice that

$$\begin{aligned} (L_\beta \gamma )^{cd}\gamma _{ac}\gamma _{bd} = - 2\nabla ^\partial _{(a}\beta _{b)} \quad ({\dot{\gamma }})^{cd}\gamma _{ac}\gamma _{bd} = - \partial _t\gamma _{ab}. \end{aligned}$$

#### Definition 41

Let $({\mathcal {F}}_{EH}(M), \Omega _{EH}(M))$ be the symplectic manifold

$$\begin{aligned} {\mathcal {F}}_{EH}(M) :=T^*[-1]\left( \mathcal {PR}^{\partial M}(M) \times {\mathfrak {X}}[1](M)\right) \end{aligned}$$

with its canonical symplectic structure, and $\mathcal {PR}^{\partial M}(M)$ denotes pseudo-Riemennian metrics on *M* such that their restriction to $\partial M$ is nondegenerate. Consider the functional

53 $$\begin{aligned} S_{EH}(M) = \int \limits _{M} \Big \{-\eta \sqrt{\gamma }(\epsilon (K_{ab}K^{ab} - K^2) +R^\partial -2\Lambda )\Big \} + {\widetilde{g}}^\dag L_{{\widetilde{\xi }}} {\widetilde{g}}+ \frac{1}{2}\iota _{[{\widetilde{\xi }},{\widetilde{\xi }}]}{\widetilde{\xi }}^{\dag } \end{aligned}$$

and denote by $Q_{EH}(M)$ the Hamiltonian vector field of $S_{EH}(M)$, up to boundary terms. Then, the assignment of the tuple

$$\begin{aligned} {\mathfrak {F}}_{EH}=({\mathcal {F}}_{EH}(M), S_{EH}(M), \Omega _{EH}(M), Q_{EH}(M))) \end{aligned}$$

to every (d+1)-dimensional manifold *M* that admits a Lorentzian structure will be called Einstein–Hilbert theory in the BV formalism.

#### Remark 42

The sign convention used above is obtained from the standard ADM decomposition by redefining $(\eta ,\beta ) \rightarrow (-\eta ,-\beta )$. This matches our conventions below. This change is due to the choice of using inverse metrics for the first order formulation, instead of metrics (in fact $\Pi _{ab} \partial _t \gamma ^{ab} = -\Pi ^{ab} \partial _t \gamma _{ab}$).

#### Theorem 43

Einstein–Hilbert theory in the BV formalism ${\mathfrak {F}}_{EH}(\Sigma \times I)$ is strongly equivalent to ${{\mathfrak {F}}}_{R}(\Sigma \times I){}$. Explicitly, the isomorphism of the underlying symplectic dg-manifolds reads:

54a $$\begin{aligned} {\widetilde{g}}&= -\eta ^{-2}\partial _t\partial _t - 2 \eta ^{-2}\beta \partial _t + \gamma -\eta ^{-2} \beta \beta \end{aligned}$$

54b $$\begin{aligned} {\widetilde{\xi }}^{}&= -\eta ^{-1} \xi ^n \partial _t + \xi ^\partial - \eta ^{-1} \xi ^n \beta \end{aligned}$$

54c $$\begin{aligned} {\widetilde{\xi }}^{\dag }&= \xi ^\dag _\partial - \left( \eta \xi ^\dag _n + \iota _\beta \xi ^\dag _\partial \right) dt \nonumber \\ {\widetilde{g}}^\dag&= \left( \frac{1}{2} \eta ^3 \varphi _n - \eta ^2 \varphi _a \beta ^a - \gamma ^\dag _{ab}\beta ^a\beta ^b + \eta \beta ^a \xi ^\dag _a \xi ^n + \frac{1}{2}\eta ^{-1}\xi ^\dag _n \xi ^n\right) dt^2 \end{aligned}$$

54d $$\begin{aligned}&\quad +\, \, \left( \frac{1}{2} \eta ^2 \varphi _a + \gamma ^\dag _{ab}\beta ^b - \frac{1}{2} \eta \xi ^\dag _a\xi ^n\right) dx^a dt - \gamma ^\dag _{ab} dx^a dx^b \end{aligned}$$

with inverse:

55a $$\begin{aligned} \eta&=[-{\widetilde{g}}^{tt}]^{-\frac{1}{2}} \end{aligned}$$

55b $$\begin{aligned} \beta ^a&= - [-{\widetilde{g}}^{tt}]^{-1} {\widetilde{g}}^{ta} \end{aligned}$$

55c $$\begin{aligned} {\gamma }^{ab}&= [-{\widetilde{g}}^{tt}]^{-1} {\widetilde{g}}^{ta} {\widetilde{g}}^{tb} \end{aligned}$$

55d $$\begin{aligned} \xi ^{n}&= - [-{\widetilde{g}}^{tt}]^{-\frac{1}{2} }{\widetilde{\xi }}^{t} \end{aligned}$$

55e $$\begin{aligned} \xi ^{a}&= {\widetilde{\xi }}^{a} + [-{\widetilde{g}}^{tt}]{\widetilde{g}}^{ta}{\widetilde{\xi }}^{t} \end{aligned}$$

55f $$\begin{aligned} {\gamma }^\dag _{ab}&= {\widetilde{g}}^\dag _{ab} \end{aligned}$$

55g $$\begin{aligned} {\varphi }_a&= 2 [-{\widetilde{g}}^{tt}]{\widetilde{g}}^\dag _{at} + 2 {\widetilde{g}}_{ab}^\dag {\widetilde{g}}^{tb} + {\widetilde{\xi }}^{\dag }_a{\widetilde{\xi }}^{t} \end{aligned}$$

55h $$\begin{aligned} {\varphi }_n&= 2[-{\widetilde{g}}^{tt}]^{\frac{3}{2} }{\widetilde{g}}^\dag _{tt} - 4[-{\widetilde{g}}^{tt}]^{\frac{1}{2} } {\widetilde{g}}^\dag _{ta} {\widetilde{g}}^{ta} + 2[-{\widetilde{g}}^{tt}]^{-\frac{1}{2} }{\widetilde{g}}^\dag _{ab} {\widetilde{g}}^{ta}{\widetilde{g}}^{tb} \end{aligned}$$

55i $$\begin{aligned}&\quad +\, [-{\widetilde{g}}^{tt}]^{\frac{1}{2} }{\widetilde{\xi }}^{\dag }_n {\widetilde{\xi }}^{t} -[-{\widetilde{g}}^{tt}]^{-\frac{1}{2}} g^{ta} {\widetilde{\xi }}^{\dag }_a{\widetilde{\xi }}^{a} \end{aligned}$$

55j $$\begin{aligned} \xi ^\dag _n&= - [-{\widetilde{g}}^{tt}]^{\frac{1}{2} }{\widetilde{\xi }}^{\dag }_n + [-{\widetilde{g}}^{tt}]^{-\frac{1}{2}}{\widetilde{\xi }}^{\dag }_a {\widetilde{g}}^{ta} \end{aligned}$$

55k $$\begin{aligned} \xi ^{\dag }_a&= {\widetilde{\xi }}^{\dag }_a. \end{aligned}$$

#### Proof

We begin observing that the definitions of *K* in (41) and *K* in (52) coincide up to sign, after identifying ${\widetilde{g}}$ with the expression of Eq. (49b). Since the expression $S_{ADM}:=-\eta \sqrt{\gamma }(\epsilon (K_{ab}K^{ab} - K^2) +R^\partial -2\Lambda )$ is quadratic in *K*, we conclude that the degree-zero part of (53) and (46) coincide. This means that the two theories are classically equivalent,^23^ and (49b) is the map between second-order and first-order Einstein–Hilbert theory.

We endeavour now to find the explicit expression for ${\widetilde{g}}^\dag$ and ${\widetilde{\xi }}^{\dag }$ so that

$$\begin{aligned} \phi ^*(\langle {\widetilde{g}}^\dag , \delta {\widetilde{g}}\rangle + \langle {\widetilde{\xi }}^{\dag }, \delta {\widetilde{\xi }}^{}\rangle ) = - \langle \delta {\gamma }^\dag , \delta {\gamma }\rangle + \langle {\Pi }, \delta {\Pi }^\dag \rangle + \xi ^\dag _\rho \delta \xi ^{\rho } + {\varphi }_n \delta \eta + {\varphi }_a\delta \beta ^a. \end{aligned}$$

It is straightforward to compute

56 $$\begin{aligned}&\phi ^*(\langle {\widetilde{g}}^\dag , \delta {\widetilde{g}}\rangle + \langle {\widetilde{\xi }}^{\dag }, \delta {\widetilde{\xi }}^{}\rangle ) \nonumber \\&\quad = - \left[ (\phi ^*{\widetilde{\xi }}^{\dag })_n\eta ^{-1} + (\phi ^*{\widetilde{\xi }}^{\dag })_a\eta ^{-1}\beta ^a\right] \delta \xi ^n + (\phi ^*{\widetilde{\xi }}^{\dag })_a\delta \xi ^a \nonumber \\&\qquad +\, \left[ 2(\phi ^*{\widetilde{g}}^\dag )_{tt} \eta ^{-3} + 4 (\phi ^*{\widetilde{g}}^\dag )_{at} \eta ^{-3} \beta ^a + 2(\phi ^*{\widetilde{g}}^\dag )_{ab} \eta ^{-3}\beta ^a\beta ^b \right] \delta \eta \nonumber \\&\qquad -\, \left[ (\phi ^*{\widetilde{\xi }}^{\dag })_t \eta ^{-2} \xi ^n - (\phi ^*{\widetilde{\xi }}^{\dag })_a \eta ^{-2} \beta ^a \xi ^n\right] \delta \eta +(\phi ^*{\widetilde{g}}^\dag )_{ab}\delta \gamma ^{ab} \nonumber \\&\qquad +\, \, \left[ 2 (\phi ^*{\widetilde{g}}^\dag )_{at}\eta ^{-2} - 2(\phi ^*{\widetilde{g}}^\dag )_{ab}\eta ^{-2}\beta ^b + (\phi ^*{\widetilde{\xi }}^{\dag })_a\eta ^{-1}\xi ^n\right] \delta \beta ^a \end{aligned}$$

which leaves us with the intermediate expression:

57a $$\begin{aligned} \xi ^\dag _a&= (\phi ^*{\widetilde{\xi }}^{\dag })_a \end{aligned}$$

57b $$\begin{aligned} \xi ^\dag _n&= - \left[ (\phi ^*{\widetilde{\xi }}^{\dag })_t\eta ^{-1} + (\phi ^*{\widetilde{\xi }}^{\dag })_a\eta ^{-1}\beta ^a\right] \end{aligned}$$

57c $$\begin{aligned} {\gamma }^\dag _{ab}&=-(\phi ^*{\widetilde{g}}^\dag )_{ab} \end{aligned}$$

57d $$\begin{aligned} {\varphi }_a&=-2 (\phi ^*{\widetilde{g}}^\dag )_{at}\eta ^{-2} - 2(\phi ^*{\widetilde{g}}^\dag )_{ab}\eta ^{-2}\beta ^b + (\phi ^*{\widetilde{\xi }}^{\dag })_a\eta ^{-1}\xi ^n \nonumber \\ {\varphi }_n&= 2(\phi ^*{\widetilde{g}}^\dag )_{tt} \eta ^{-3} + 4 (\phi ^*{\widetilde{g}}^\dag )_{at} \eta ^{-3} \beta ^a + 2(\phi ^*{\widetilde{g}}^\dag )_{ab} \eta ^{-3}\beta ^a\beta ^b \end{aligned}$$

57e $$\begin{aligned}&\quad -\, (\phi ^*{\widetilde{\xi }}^{\dag })_t \eta ^{-2} \xi ^n - (\phi ^*{\widetilde{\xi }}^{\dag })_a \eta ^{-2} \beta ^a \xi ^n \end{aligned}$$

Starting from the top and solving downwards, we easily get

58 $$\begin{aligned} (\phi ^*{\widetilde{\xi }}^{\dag })_a&= \xi ^\dag _a \end{aligned}$$

59 $$\begin{aligned} (\phi ^*{\widetilde{\xi }}^{\dag })_n&= - \eta \xi ^\dag _n - \xi ^\dag _a \beta ^a \end{aligned}$$

60 $$\begin{aligned} (\phi ^*{\widetilde{g}}^\dag )_{ab}&= -\gamma ^\dag _{ab} \end{aligned}$$

61 $$\begin{aligned} (\phi ^*{\widetilde{g}}^\dag )_{at}&= -\frac{1}{2}\eta ^2{\varphi }_a + {\gamma }^\dag _{ab}\beta ^b + \frac{1}{2} \eta \xi ^\dag _a\xi ^n \end{aligned}$$

62 $$\begin{aligned} (\phi ^*{\widetilde{g}}^\dag )_{tt}&= \frac{1}{2} \eta ^{3} {\varphi }_n + \eta ^{2}{\varphi }_a\beta ^a - {\gamma }^\dag _{ab}\beta ^a\beta ^b - \eta \xi ^\dag _a\beta ^a\xi ^n - \frac{1}{2} \eta \xi ^\dag _n\xi ^n \end{aligned}$$

Alternatively, from (57), observing that the assignment (49) can be inverted to yield

$$\begin{aligned} \phi ^{-1}{}^*\eta = [-{\widetilde{g}}^{tt}]^{-\frac{1}{2}}, \quad \phi ^{-1}{}^*\beta ^a = - [-{\widetilde{g}}^{tt}]^{-1} {\widetilde{g}}^{ta}, \quad \phi ^{-1}{}^*\gamma ^{ab} = [-{\widetilde{g}}^{tt}]^{-1} {\widetilde{g}}^{ta}{\widetilde{g}}^{tb} \end{aligned}$$

together with

$$\begin{aligned} \phi ^{-1}{}^*\xi ^n = -[-{\widetilde{g}}^{tt}]^{-\frac{1}{2}}{\widetilde{\xi }}^{t}; \quad \phi ^{-1}{}^*\xi ^a = {\widetilde{\xi }}^{a} + [-{\widetilde{g}}^{tt}]^{-1}{\widetilde{g}}^{ta}{\widetilde{\xi }}^{t} \end{aligned}$$

we can similarly obtain the inverse:

$$\begin{aligned} \phi ^{-1}{}^*\eta&=[-{\widetilde{g}}^{tt}]^{-\frac{1}{2}}\\ \phi ^{-1}{}^*\beta ^a&= - [-{\widetilde{g}}^{tt}]^{-1} {\widetilde{g}}^{ta}\\ \phi ^{-1}{}^*{\gamma }^{ab}&= [-{\widetilde{g}}^{tt}]^{-1} {\widetilde{g}}^{ta} {\widetilde{g}}^{tb}\\ \phi ^{-1}{}^*\xi ^{n}&= - [-{\widetilde{g}}^{tt}]^{-\frac{1}{2} }{\widetilde{\xi }}^{t}\\ \phi ^{-1}{}^*\xi ^{a}&= {\widetilde{\xi }}^{a} + [-{\widetilde{g}}^{tt}]{\widetilde{g}}^{ta}{\widetilde{\xi }}^{t}\\ \phi ^{-1}{}^*{\gamma }^\dag _{ab}&= -{\widetilde{g}}^\dag _{ab}\\ \phi ^{-1}{}^*\xi ^{\dag }_a&= {\widetilde{\xi }}^{\dag }_a\\ \phi ^{-1}{}^*\xi ^\dag _n&= - {\widetilde{\xi }}^{\dag }_n[-{\widetilde{g}}^{tt}]^{\frac{1}{2} } + {\widetilde{\xi }}^{\dag }_a [-{\widetilde{g}}^{tt}]^{-\frac{1}{2}}{\widetilde{g}}^{ta}\\ \phi ^{-1}{}^*{\varphi }_a&= - 2 {\widetilde{g}}^\dag _{at} [-{\widetilde{g}}^{tt}] + 2 {\widetilde{g}}_{ab}^\dag {\widetilde{g}}^{tb} - {\widetilde{\xi }}^{\dag }_a{\widetilde{\xi }}^{t}\\ \phi ^{-1}{}^*{\varphi }_n&= 2{\widetilde{g}}^\dag _{tt} [-{\widetilde{g}}^{tt}]^{\frac{3}{2} } - 4 {\widetilde{g}}^\dag _{ta}[-{\widetilde{g}}^{tt}]^{\frac{1}{2} } {\widetilde{g}}^{ta} + 2{\widetilde{g}}^\dag _{ab}[-{\widetilde{g}}^{tt}]^{-\frac{1}{2} } {\widetilde{g}}^{ta}{\widetilde{g}}^{tb} \\&\quad +\, \, [-{\widetilde{g}}^{tt}]^{\frac{1}{2} }{\widetilde{\xi }}^{\dag }_t {\widetilde{\xi }}^{t} -[-{\widetilde{g}}^{tt}]^{-\frac{1}{2}} g^{ta} {\widetilde{\xi }}^{\dag }_a{\widetilde{\xi }}^{t}. \end{aligned}$$

Now, using again the intermediate expressions (57) let us consider the following terms, coming from Eq. (46):

$$\begin{aligned}&\xi ^\dag _n L_{\xi ^\partial }\xi ^n = -(\phi ^*{\widetilde{\xi }}^{\dag })_t \eta ^{-1}L_{\xi ^\partial }\xi ^n - (\phi ^*{\widetilde{\xi }}^{\dag })_a\eta ^{-1}\beta ^a L_{\xi ^\partial }\xi ^n\\&\langle \xi ^\dag _\partial ,(\xi ^n\nabla _{{\gamma }}\xi ^n + \frac{1}{2}[\xi ^\partial ,\xi ^\partial ]\rangle = \langle (\phi ^*{\widetilde{\xi }}^{\dag })_\partial ,(\xi ^n\nabla _{{\gamma }}\xi ^n + \frac{1}{2}[\xi ^\partial ,\xi ^\partial ]\rangle {\varphi }_n \left( L_{\xi ^\partial }\eta - L_{\beta }\xi ^n - {\dot{\xi }}^n\right) \\&= \eta ^{-3}\left[ 2(\phi ^*{\widetilde{g}}^\dag )_{tt} + 4 (\phi ^*{\widetilde{g}}^\dag )_{at}\beta ^a + (\phi ^*{\widetilde{g}}^\dag )_{ab}\beta ^a\beta ^b\right] \left( L_{\xi ^\partial }\eta -L_\beta \xi ^n -{\dot{\xi }}^n\right) \\&\quad -\, \eta ^{-2}\left[ (\phi ^*{\widetilde{\xi }}^{\dag })_n \xi ^n + (\phi ^*{\widetilde{\xi }}^{\dag })_a \beta ^a \xi ^n\right] \left( L_{\xi ^\partial }\eta -L_{\beta }\xi ^n - {\dot{\xi }}^n\right) \\&\langle {\varphi }_\partial ,\left( \nabla _{{\gamma }}\xi ^n -\eta \nabla _{{\gamma }}\xi ^n + L_{\xi ^\partial }\beta \right) \rangle \\&\quad =-2\eta ^{-2}\left( (\phi ^*{\widetilde{g}}^\dag )_{at} +(\phi ^*{\widetilde{g}}^\dag )_{ab}\beta ^b\right) \left( (\nabla _{{\gamma }}\eta )^a\xi ^n - \eta (\nabla _{{\gamma }}\xi ^n)^a +(L_{\xi ^\partial }\beta )^a - {\dot{\xi }}^a\right) \\&\quad +\, (\phi ^*{\widetilde{\xi }}^{\dag })_a \eta ^{-1} \xi ^n\left( (L_{\xi ^\partial }\beta )^a -\eta (\nabla _{{\gamma }}\xi ^n)^a - {\dot{\xi }}^a\right) \\&\langle {\gamma }^\dag , \eta ^{-1} \left( {\dot{{\gamma }}} + L_\beta {\gamma }\right) \xi ^n - L_{\xi ^\partial }{\gamma }\rangle = -(\phi ^*{\widetilde{g}}^\dag )_{ab}\left( \eta ^{-1}\left( {\dot{{\gamma }}} + L_\beta {\gamma }\right) \xi ^n - L_{\xi ^\partial }{\gamma }\right) ^{ab}. \end{aligned}$$

Then, summing all terms on the left hand side and factoring $(\phi ^*{\widetilde{\xi }}^{\dag })_t$, $(\phi ^*{\widetilde{\xi }}^{\dag })_a$ and $(\phi ^*{\widetilde{g}}^{\dag })$, we obtain

$$\begin{aligned}&(\phi ^*{\widetilde{\xi }}^{\dag })_t\left[ -L_{\xi ^\partial }(\eta ^{-1}\xi ^n) +\eta ^{-2}\xi ^n\left( L_{\beta }\xi ^n + {\dot{\xi }}^n\right) \right] \\&\quad +\, (\phi ^*{\widetilde{g}}^\dag )_{tt}\left[ 2\eta ^{-3}\left( L_{\xi ^\partial }\eta - L_\beta \xi ^n - {\dot{\xi }}^n\right) \right] +\left\langle (\phi ^*{\widetilde{\xi }}^{\dag })_\partial , \frac{1}{2}[\xi ^\partial ,\xi ^\partial ] \right\rangle \\&\quad +\, \, \left\langle (\phi ^*{\widetilde{\xi }}^{\dag })_\partial , - L_{\xi ^\partial }(\eta ^{-1}\xi ^n)\beta + \eta ^{-1}L_{\xi ^\partial }\beta \xi ^n + \eta ^{-2} \beta \xi ^n L_\beta \xi ^n + \eta ^{-2}\beta \xi ^n {\dot{\xi }}^n - \eta ^{-1}\xi ^n {\dot{\xi }}^\partial \right\rangle \\&\quad +\, (\phi ^*{\widetilde{g}}^\dag )_{ab}\left[ -\eta ^{-1}{\dot{{\gamma }}}^{ab}\xi ^n - \eta ^{-1}(L_\beta {\gamma })^{ab}\xi ^n + (L_{\xi ^\partial }{\gamma })^{ab} + 4\eta ^{-3} \beta ^a\left( L_{\xi ^\partial }\eta - L_\beta \xi ^n - {\dot{\xi }}^n\right) \right] \\&\quad +\, (\phi ^*{\widetilde{g}}^\dag )_{ab}\left[ -2\eta ^{-2}\left( (\nabla _{{\gamma }}\eta )^a\xi ^n - \eta (\nabla _{{\gamma }}\xi ^n)^a +(L_{\xi ^\partial }\beta )^a - {\dot{\xi }}^a\right) \right] \\&\quad +\, (\phi ^*{\widetilde{g}}^\dag )_{ab}\left[ 2\eta ^{-3}\beta ^a\beta ^b\left( L_{\xi ^\partial }\eta - L_\beta \xi ^n - {\dot{\xi }}^n\right) \right] \\&\quad +\, \, (\phi ^*{\widetilde{g}}^\dag )_{ab}\left[ -2\eta ^{-2}\beta ^b\left( (\nabla _{{\gamma }}\eta )^a\xi ^n - \eta (\nabla _{{\gamma }}\xi ^n)^a +(L_{\xi ^\partial }\beta )^a - {\dot{\xi }}^a\right) \right] \end{aligned}$$

Which, using Lemma 39, can be shown to be

63 $$\begin{aligned} \phi ^*\left( {\widetilde{g}}^\dag L_{{\widetilde{\xi }}}{\widetilde{g}}+ \iota _{[{\widetilde{\xi }},{\widetilde{\xi }}]}{\widetilde{\xi }}^\dag \right) \end{aligned}$$

leading to

64 $$\begin{aligned} \phi ^*S_{EH}(\Sigma \times I) = {S}_{R}(\Sigma \times I){}. \end{aligned}$$

$$\square $$

#### Remark 44

We would like to stress here that the results in this section are a “strictification” of the general construction of a solution of the classical master equation for the extended Hamiltonian, as presented by Henneaux and Bunster [HT92, Theorem 18.8]. Indeed, the Hamiltonian analysis for a field theory relies on a (possibly) non-reduced version of the strict BFV data we consider, where strict indicates that we require all spaces of fields to be smooth symplectic manifolds. The AKSZ construction yields a BV theory (Theorem 32) which is effectively equivalent to the natural BV extension of Einstein–Hilbert theory (Theorems 37, 43). It could be argued that this effective equivalence preserves the BV cohomology [BBH95, DGH90, Hen90]. However, note that the quantisation procedure outlined in [CMR18] does indeed require the strict version of a BV–BFV structure,^24^ and its existence is not to be taken for granted, as was shown in [CS19b, CS17].

## AKSZ PC

Following the construction outlined in Sect. 1.3, starting from the BFV theory of Palatini–Cartan gravity (see Sect. 2.2), we can construct the AKSZ space of fields ${\mathcal {F}}_{PC}^{\textsf {\tiny AKSZ}}$. We will use the following notation:

65a $$\begin{aligned} {\mathfrak {e}}&= e + f^\dag&{\mathfrak {w}}&= \omega + u^\dag \end{aligned}$$

65b $$\begin{aligned} {\mathfrak {c}}&= c + w&{\mathfrak {x}}&=\xi + z \end{aligned}$$

65c $$\begin{aligned} {\mathfrak {l}}&= \lambda + \mu&{\mathfrak {c}}^\dag&= k^\dag + c^\dag \end{aligned}$$

65d $$\begin{aligned} {\mathfrak {y}}^\dag&= e^\dag + y^\dag&\end{aligned}$$

where

66 $$\begin{aligned} \begin{aligned} e&\in C^{\infty }(I)\otimes \Omega _{nd}^1(\Sigma , {\mathcal {V}})&f^\dag&\in \Omega ^1[-1](I) \otimes \Omega ^1(\Sigma , {\mathcal {V}}) \\ \omega&\in C^{\infty }(I)\otimes {\mathcal {A}}^{}(\Sigma )&u^\dag&\in \Omega ^1[-1](I)\otimes {\mathcal {A}}^{}(\Sigma ) \\ c&\in \Omega ^0[1]( I \times \Sigma , \textstyle {\bigwedge ^2}{\mathcal {V}})&w&\in \Omega ^1[-1](I)\otimes \Omega ^0[1]( \Sigma , \textstyle {\bigwedge ^2}{\mathcal {V}}) \\ \xi&\in C^{\infty }(I)\otimes {\mathfrak {X}}[1](\Sigma )&z&\in \Omega ^1[-1](I)\otimes {\mathfrak {X}}[1](\Sigma ) \\ \lambda&\in C^\infty [1](I \times \Sigma )&\mu&\in \Omega ^1[-1](I)\otimes C^\infty [1](\Sigma ) \\ k^\dag&\in C^{\infty }(I)\otimes \Omega ^{N-1}[-1](\Sigma ,\textstyle {\bigwedge ^{N-2}} {\mathcal {V}})&c^\dag&\in \Omega ^1[-1](I)\otimes \Omega ^{N-1}[-1](\Sigma ,\textstyle {\bigwedge ^{N-2}} {\mathcal {V}})\\ e^\dag&\in C^{\infty }(I)\otimes \Omega ^{N-1}[-1](\Sigma ,\textstyle {\bigwedge ^{N-1}} {\mathcal {V}})&y^\dag&\in \Omega ^1[-1](I)\otimes \Omega ^{N-1}[-1](\Sigma ,\textstyle {\bigwedge ^{N-1}} {\mathcal {V}}) \end{aligned} \end{aligned}$$

such that, for some $\sigma \in C^{\infty }(I)\otimes \Omega ^1(\Sigma , {\mathcal {V}})$ and $B\in \Omega ^1[-1](I) \otimes \Omega ^1(\Sigma , {\mathcal {V}})$, they satisfy the *structural AKSZ constraints*:

67a $$\begin{aligned}&\epsilon _n \left\{ (N-4)f^\dag e^{N-5} d_\omega e + e^{N-4} d_{\omega } f^\dag + e^{N-4} [u^\dag , e]\right\} \nonumber \\&\quad +\left( \iota _z d_{\omega }\epsilon _n\!-\![w-\iota _\xi u^\dag , \epsilon _n]\right) ^{\!(a)} k^\dag _a+ X^{(a)} c^\dag _a + \left( X^{(b)}f^\dag _b\right) ^{(a)}k^\dag _a= f^\dag e^{N-4} \sigma + e^{N-3} B; \end{aligned}$$

67b $$\begin{aligned}&\epsilon _n e^{N-4} d_{\omega } e + X^{(a)} k^\dag _a = e^{N-3}\sigma ; \end{aligned}$$

where $X= \left( L_\xi ^\omega \epsilon _n - [c,\epsilon _n]\right) \in \Gamma (M,{\mathcal {V}})$, while $\epsilon _n\in \Gamma (M,{\mathcal {V}})$ is a fixed section, and the indices $\{(a),(n)\}$ denote components with respect to a basis $\{e_a, \epsilon _n\}$.

### Remark 45

Observe that our target for the AKSZ construction for Palatini–Cartan theory is the BFV theory defined in Definition 26, whose space of fields ${\mathcal {F}}_{PC}^\partial$ is defined by the structural constraint (19). As a consequence, the BFV constraint must be imposed on the AKSZ fields at every point of *I*. As the AKSZ fields consists of a 0- and 1-form component, along the interval, the structural constraints now has a 0-form and a 1- form component corresponding to (67b) and (67a), respectively. Despite the apparent complexity of these two equations, it is worth noting that they fix certain components of the AKSZ fields $\omega$ and $u^\dag$. See Sect. 4.2 for an interpretation.

### Remark 46

Recall that to define the BFV structure for PC theory we needed a fixed section $\epsilon _n\in \Gamma (\Sigma ,{\mathcal {V}})$ (cf. Definition 25). Note that $\epsilon _n$ is not a field of the theory but is part of the structure that defines it (more like a coupling constant). For this reason, in the AKSZ construction $\epsilon _n$ does not depend on the coordinate $x^n$ of the interval *I*. In the following, we will regard $\epsilon _n$ as a given section of $\Gamma (M,V)$ satisfying $d_I(\epsilon _n)=0$.

### Theorem 47

The AKSZ data ${\mathfrak {F}}_{PC}^{\textsf {\tiny AKSZ}}(I; {\mathfrak {F}}_{PC}^{\partial })$ are given by the quadruple

$$\begin{aligned} {\mathfrak {F}}_{PC}^{\textsf {\tiny AKSZ}}(I; {\mathfrak {F}}_{PC}^{\partial })=({\mathcal {F}}_{PC}^{\textsf {\tiny AKSZ}}, S_{PC}^{\textsf {\tiny AKSZ}}, \varpi _{PC}^{\textsf {\tiny AKSZ}}, Q_{PC}^{\textsf {\tiny AKSZ}}) \end{aligned}$$

where:

$$\begin{aligned} {\mathcal {F}}_{PC}^{\textsf {\tiny AKSZ}}&\simeq T^*[-1](\mathrm {Map} (I, {\mathcal {F}}^{\partial }_{PC})) \\ \varpi _{PC}^{\textsf {\tiny AKSZ}}&= \int _{I\times \Sigma } \delta (e^{N-3} f^\dag ) \delta \omega +e^{N-3} \delta e \delta u^\dag + \delta w \delta k^\dag + \delta c \delta c^\dag + \delta u^\dag \delta (\iota _\xi k^\dag )\\&\quad +\, \, \delta \omega \delta (\iota _z k^\dag )+ \delta \omega \delta (\iota _\xi c^\dag ) - \delta \mu \epsilon _n \delta e^\dag - \delta \lambda \epsilon _n \delta y^\dag \\&\quad +\, \iota _{\delta z}\delta (e e^\dag ) +\iota _{\delta \xi } \delta (f^\dag e^\dag )+\iota _{\delta \xi } \delta (e y^\dag ); \\ S_{PC}^{\textsf {\tiny AKSZ}}&=\int _{I\times \Sigma } w e^{N-3} d_{\omega } e + (N-3) c e^{N-4} f^\dag d_{\omega } e + c e^{N-3} [u^\dag , e] + c e^{N-3} d_{\omega } f^\dag \\&\quad +\, \iota _{z} e e^{N-3} F_{\omega } + \iota _{\xi }( e^{N-3} f^\dag ) F_{\omega } + \iota _{\xi } e e^{N-3} d_{\omega }u^\dag + \epsilon _n \mu e^{N-3} F_{\omega } \\&\quad +\, (N-3) \epsilon _n \lambda e^{N-4} f^\dag F_{\omega } + \epsilon _n \lambda e^{N-3} d_{\omega }u^\dag + [w,c] k^{\dag } + \frac{1}{2} [c,c] c^{\dag } \\&\quad -\, \iota _{z} d_{\omega } c k^{\dag } - [\iota _{\xi }u^\dag ,c] k^{\dag } - \iota _{\xi } d_{\omega }w k^{\dag } - \iota _{\xi } d_{\omega } c c^{\dag } + \iota _{z}\iota _{\xi } F_{\omega }k^{\dag }\\&\quad +\, \frac{1}{2} \iota _{\xi }\iota _{\xi } d_{\omega }u^\dag k^{\dag } + \frac{1}{2} \iota _{\xi }\iota _{\xi } F_{\omega }c^{\dag } - [w, \epsilon _n \lambda ]e^{\dag } - [c, \epsilon _n \mu ]e^{\dag } - [c, \epsilon _n \lambda ]y^{\dag } \\&\quad +\, \iota _{z}d_{\omega } (\epsilon _n \lambda )e^{\dag } + [\iota _{\xi }u^\dag ,\epsilon _n \lambda ]e^{\dag } + \iota _{\xi }d_{\omega } (\epsilon _n \mu )e^{\dag } + \iota _{\xi }d_{\omega } (\epsilon _n \lambda )y^{\dag }\\&\quad +\, \iota _{[z,\xi ]}e e^{\dag } + \frac{1}{2}\iota _{[\xi ,\xi ]}f^\dag e^{\dag } + \frac{1}{2}\iota _{[\xi ,\xi ]}e y^{\dag } \\&\quad +\, \frac{1}{N-2} e^{N-2} d_I \omega + c d_I k^\dag + d_I \omega \iota _\xi k^\dag - \iota _{d_I \xi } e e^\dag + d_I \lambda \epsilon _n e^\dag . \end{aligned}$$

and $Q_{PC}^{\textsf {\tiny AKSZ}}$ is defined as $Q_{PC}^{\textsf {\tiny AKSZ}}= Q_{PC}^{\text {dR}} + Q_{PC}^{\text {lift}}$ where ${Q}_{PC}^{\text {lift}}$ is the tangent lift of $Q^{\partial }_{PC}$ to $\mathrm {Map}(T[1]I,{\mathcal {F}}_{PC}^\partial (\Sigma ))$ and $Q_{PC}^{dR}$ is the lift of the de Rham differential $d_I$.

### Proof

This is a straightforward application of the AKSZ prescription outlined in Sect. 1.3. Using the transgression map we can build a symplectic form ${\mathcal {F}}_{PC}^{\textsf {\tiny AKSZ}}$

68 $$\begin{aligned} \varpi ^{\textsf {\tiny AKSZ}}_{PC} = \int _{I\times \Sigma }&{\mathfrak {e}}^{N-3} \delta {\mathfrak {e}} \delta {\mathfrak {w}} + \delta {\mathfrak {c}} \delta {\mathfrak {c}}^\dag + \delta {\mathfrak {w}} \delta (\iota _{{\mathfrak {x}}} {\mathfrak {c}}^\dag ) - \delta {\mathfrak {l}} e_n \delta {\mathfrak {y}}^\dag + \iota _{\delta {\mathfrak {x}}} \delta ({\mathfrak {e}}{\mathfrak {y}}^\dag ) \end{aligned}$$

from which we obtain the claimed expression using (65). Analogously the AKSZ action can be constructed using the transgression map from the boundary one-form $\alpha ^{\partial }$ and from the boundary action $S^{\partial }$. Namely we have

69 $$\begin{aligned} S^{\textsf {\tiny AKSZ}}_{PC}&=\int _{I\times \Sigma } \frac{1}{N-2} {\mathfrak {e}}^{N-2} d_I {\mathfrak {w}} + {\mathfrak {c}} d_I {\mathfrak {c}}^\dag + d_I {\mathfrak {w}} \iota _{{\mathfrak {x}}} {\mathfrak {c}}^\dag - \iota _{d_I {\mathfrak {x}}} {\mathfrak {e}} {\mathfrak {y}}^\dag + d_I {\mathfrak {l}} \epsilon _n {\mathfrak {y}}^\dag \nonumber \\&\quad {\mathfrak {c}} {\mathfrak {e}} d_{{\mathfrak {w}}} {\mathfrak {e}} + \iota _{{\mathfrak {x}}} {\mathfrak {e}} {\mathfrak {e}} F_{{\mathfrak {w}}} + \epsilon _n {\mathfrak {l}} {\mathfrak {e}} F_{{\mathfrak {w}}} +\frac{1}{2} [{\mathfrak {c}},{\mathfrak {c}}] {\mathfrak {c}}^{\dag } - L^{{\mathfrak {w}}}_{{\mathfrak {x}}} {\mathfrak {c}} {\mathfrak {c}}^{\dag }+ \frac{1}{2} \iota _{{\mathfrak {x}}}\iota _{{\mathfrak {x}}} F_{{\mathfrak {w}}}{\mathfrak {c}}^{\dag }\nonumber \\&\quad -[{\mathfrak {c}}, \epsilon _n {\mathfrak {l}} ]{\mathfrak {y}}^{\dag } + L^{{\mathfrak {w}}}_{{\mathfrak {x}}} (\epsilon _n {\mathfrak {l}}){\mathfrak {y}}^{\dag } + \frac{1}{2}\iota _{[{\mathfrak {x}},{\mathfrak {x}}]}{\mathfrak {e}} {\mathfrak {y}}^{\dag }. \end{aligned}$$

Again the claimed expression can be obtained straightforwardly from (65). $\square$

### Remark 48

The invariance of the constraints (67b) and (67a) with respect to $Q_{PC}^{\textsf {\tiny AKSZ}}$ is guaranteed by the invariance of the structural constraint on the boundary (19) with respect to $Q^{{{\,\mathrm{\textit{BFV}}\,}}}_{PC}$, and by the properties of the AKSZ construction.

From Theorem 14 we know that ${\mathfrak {F}}_{PC}^{\textsf {\tiny AKSZ}}(I; {\mathfrak {F}}_{PC}^{\partial })$ yields a BV theory on the manifold $I \times \Sigma$. Furthermore, by Proposition 17 these data satisfy also the BV–BFV axioms of Eq. (5).

### Definition 49

We call *nondegenerate* AKSZ PC theory the data ${\mathfrak {F}}_{PC_\star }^{\textsf {\tiny AKSZ}}$ obtained by restricting the space of fields of ${\mathfrak {F}}_{PC}^{\textsf {\tiny AKSZ}}(I;{\mathfrak {F}}^\partial _{PC}(\Sigma ))$ to those maps whose $\mu$ component [as defined by Eq. (65)] is nonvanishing.

In [CS19b] two of the authors proved that, using the natural symmetries of PC theory, the resulting BV theory ${\mathfrak {F}}_{PC}$ does not satisfy the BV–BFV axioms (it is not a 1-extended BV theory) unless additional requirements on the fields are enforced. Next section will be devoted to the comparison between ${\mathfrak {F}}_{PC}(\Sigma \times I)$ and ${\mathfrak {F}}_{PC_\star }^{\textsf {\tiny AKSZ}}(I;{\mathfrak {F}}^\partial _{PC}(\Sigma ))$.

### Comparison of BV data for PC theory

We want to compare the AKSZ-BV theory of Theorem 47 with the one proposed for PC-gravity by two of the authors [CS19b], which we briefly recall here. Let *M* be an *N*-dimensional manifold with $N>2$.

#### Definition 50

We call *standard* BV theory for PC gravity the BV data

$$\begin{aligned} {\mathfrak {F}}_{PC}(M)= ({\mathcal {F}}_{PC}(M), S_{PC}(M), \varpi _{PC}(M), Q_{PC}(M)) \end{aligned}$$

where

$$\begin{aligned} {\mathcal {F}}_{PC}(M):=T^*[-1]\left( \Omega _{nd}^1(M, {\mathcal {V}}) \oplus {\mathcal {A}}(M) \oplus {\mathfrak {X}}[1](M) \oplus \Omega ^0[1](M,\mathrm {ad}P)\right) \end{aligned}$$

and the fields in the base are denoted by $({\mathbf {e}}, \varvec{\omega }, \varvec{\xi }^{}, {\mathbf {c}})$, while the corresponding variables in the cotangent fibre are denoted by $({\mathbf {e}}^{\dag }, \varvec{\omega }^{\dag }, \varvec{\xi }^{\dag }, {\mathbf {c}}^{\dag })$;

$$\begin{aligned} \varpi _{PC}(M)&= \int _M \delta {\mathbf {e}}\delta {\mathbf {e}}^{\dag } + \delta \varvec{\omega }\delta \varvec{\omega }^{\dag }+ \delta {\mathbf {c}}\delta {\mathbf {c}}^{\dag } + \iota _{\delta \varvec{\xi }^{}} \delta \varvec{\xi }^{\dag }; \\ S_{PC}(M)&=\int _M \frac{1}{N-2} {\mathbf {e}}^{N-2} F_{\varvec{\omega }} + \left( \iota _{\varvec{\xi }^{}} F_{\varvec{\omega }} - d_{\varvec{\omega }} {\mathbf {c}}\right) \varvec{\omega }^\dag - \left( L_{\varvec{\xi }^{}}^{\varvec{\omega }}{\mathbf {e}}- [{\mathbf {c}},{\mathbf {e}}]\right) {\mathbf {e}}^\dag \\&\quad +\, \int _M \frac{1}{2}\left( \iota _{\varvec{\xi }^{}}\iota _{\varvec{\xi }^{}} F_{\varvec{\omega }} - [{\mathbf {c}},{\mathbf {c}}]\right) {\mathbf {c}}^\dag +\frac{1}{2}\iota _{[\varvec{\xi }^{},\varvec{\xi }^{}]}\varvec{\xi }^{\dag }. \end{aligned}$$

The explicit expression of the cohomological vector field $Q_{PC}$, defined by the equation $\iota _{Q_{PC}}\varpi _{PC}= \delta S_{PC}$, will be useful in the following:

$$\begin{aligned} Q_{PC} {\mathbf {e}}&= L_{\varvec{\xi }^{}}^{\varvec{\omega }}{\mathbf {e}}- [{\mathbf {c}},{\mathbf {e}}] \\ Q_{PC} \varvec{\omega }&= \iota _{\varvec{\xi }^{}} F_{\varvec{\omega }} - d_{\varvec{\omega }} {\mathbf {c}}\\ Q_{PC} {\mathbf {c}}&= \frac{1}{2}\iota _{\varvec{\xi }^{}}\iota _{\varvec{\xi }^{}} F_{\varvec{\omega }} - \frac{1}{2}[{\mathbf {c}},{\mathbf {c}}]\\ Q_{PC} \varvec{\xi }^{}&= \frac{1}{2} [\varvec{\xi }^{}, \varvec{\xi }^{}]\\ Q_{PC} {\mathbf {e}}^{\dag }&= {\mathbf {e}}^{N-3} F_{\varvec{\omega }} + L_{\varvec{\xi }^{}}^{\varvec{\omega }} {\mathbf {e}}^{\dag } - [{\mathbf {c}}, {\mathbf {e}}^{\dag }]\\ Q_{PC} \varvec{\omega }^{\dag }&= {\mathbf {e}}^{N-3} d_{\varvec{\omega }} {\mathbf {e}}- d_{\varvec{\omega }} \iota _{\varvec{\xi }^{}} \varvec{\omega }^{\dag } - [{\mathbf {c}}, \varvec{\omega }^{\dag }] + \iota _{\varvec{\xi }^{}}[{\mathbf {e}}, {\mathbf {e}}^{\dag }]-\frac{1}{2} d_{\varvec{\omega }} \iota _{\varvec{\xi }^{}} \iota _{\varvec{\xi }^{}} {\mathbf {c}}^{\dag }\\ Q_{PC} {\mathbf {c}}^{\dag }&= - d_{\varvec{\omega }} \varvec{\omega }^\dag - [{\mathbf {e}}, {\mathbf {e}}^{\dag }] - [{\mathbf {c}}, {\mathbf {c}}^{\dag }] \\ Q_{PC} \varvec{\xi }^{\dag }_{\bullet }&= F_{\varvec{\omega }\bullet } \varvec{\omega }^\dag - (d_{\varvec{\omega }\bullet }{\mathbf {e}}){\mathbf {e}}^{\dag }+\iota _{\varvec{\xi }^{}}F_{\varvec{\omega }\bullet } {\mathbf {c}}^\dag + L_{\varvec{\xi }^{}}^{\varvec{\omega }}\varvec{\xi }^{\dag }_{\bullet } + (d_{\varvec{\omega }}\iota _{\varvec{\xi }^{}} \varvec{\xi }^{\dag })_{\bullet }. \end{aligned}$$

Here we used the symbol $\bullet$ to remind the reader that $\varvec{\xi }^\dag$ is a one-form with values in densities, and on the right hand side we highlight the one-form part of the expression.

#### Remark 51

Throughout the analysis we should always keep in mind that, while Definition 50 is valid for any manifold *M* (possibly with boundary), the AKSZ theory obtained in Theorem 47 is by construction defined on a manifold diffeomorphic to a cylinder: $M=\Sigma \times I$. Furthermore, as we will see in this section, the fields in ${\mathcal {F}}^{\textsf {\tiny AKSZ}}_{PC^\star }$ correspond to those in the standard BV theory for PC but with an additional constraint.

The product structure of *M* induces a splitting of fields:

70 $$\begin{aligned} \begin{aligned} {\mathbf {e}}&= {\widetilde{e}}+ {{\widetilde{e}}}_ndx^n&{\mathbf {e}}^\dag&= {\widetilde{e}}{}^\dag + {{\widetilde{e}}}^{\dag }_ndx^n \\ \varvec{\omega }&= {\widetilde{\omega }}+ {{\widetilde{\omega }}}_ndx^n&\varvec{\omega }^\dag&= {\widetilde{\omega }}{}^\dag + {{\widetilde{\omega }}}^{\dag }_ndx^n \\ \varvec{\xi }&= {\widetilde{\xi }}+ {{\widetilde{\xi }}}{}^n\partial _n&{\varvec{\xi }}^\dag&= {\underline{{\widetilde{\xi }}}}{}^\dag + {{\widetilde{\xi }}}^{\dag }_ndx^n \end{aligned} \end{aligned}$$

More compactly we can write field components in the $x^n$ direction as ${\underline{{\widetilde{e}}}}_n = {\widetilde{e}}_n dx^n$, ${\underline{{\widetilde{\xi }}}}{}^n= {\widetilde{\xi }}{}^n\partial _n$ and so on. Observe that $\varvec{\xi }{}^\dag$ is a one-form with values in densities on *M*, so we can identify two $dx^n$ contributions: we denote by ${\underline{{\widetilde{\xi }}}}_n{}^{\!\!\!\dag }$ the $dx^n$-component (of the one-form part) of $\varvec{\xi }{}^\dag$ and by ${\underline{{\widetilde{\xi }}}}$ the rest, stressing that the image of ${\underline{{\widetilde{\xi }}}}$ is nontrivial along $dx^n$. This decomposition allows us to define the maps

71 $$\begin{aligned} {W}_{{\widetilde{e}}{}^{N-3}}^{i,j}{:}\,\Omega ^i\left( M,\textstyle {\bigwedge ^j}{\mathcal {V}}\right) \rightarrow \Omega ^{i+N-3}\left( M,\textstyle {\bigwedge ^{j+N-3}}{\mathcal {V}}\right) ; \quad {W}_{{\widetilde{e}}{}^{N-3}}^{i,j}(v) = {\widetilde{e}}{}^{N-3} \wedge v. \end{aligned}$$

Let us now fix a nonzero section $\epsilon _n \in \Gamma (M,{\mathcal {V}})$ such that $d_I\epsilon _n=0$. We will then restrict the field ${\widetilde{e}}$ not to have components parallel to $\epsilon _n$. This is a restriction on the space of fields (it actually defines an open subspace). The nondegeneracy of *e* implies that (*i*) ${\tilde{e}}$ and $\epsilon _n$ form a basis of *V* at every point, and (*ii*) ${\widetilde{e}}_n$ becomes a linear combination of ${\widetilde{e}}$ and $\epsilon _n$, with nonzero $\epsilon _n$-component. Denote by $X^{\{\mu \}}$ the components of a field *X* with respect to the basis given by ${\widetilde{e}}$ and $\epsilon _n$ (i.e. $X = X^{\{b\}}{\widetilde{e}}_b+X^{\{n\}}{\epsilon }_n$).

Additionally, we consider the quantity

72 $$\begin{aligned} {\mathfrak {W}}^\dag := {\underline{{\widetilde{\omega }}}}^{\dag }_n - {\widetilde{\omega }}_a{}^{\!\!\!\dag } {{\widetilde{e}}}_n^{\{a\}}dx^n- \iota _{{\widetilde{\xi }}}{\underline{{\widetilde{c}}}}_n{}^{\!\!\!\dag }+{{\widetilde{c}}}_{an}{}^{\!\!\!\dag }{{\widetilde{\xi }}}{}^n{{\widetilde{e}}}_n^{\{a\}}dx^n. \end{aligned}$$

Its meaning will become manifest with the following:

#### Definition 52

We denote by $\iota _R{:}\,{\mathcal {F}}_{PC}^{\text {res}} \rightarrow {\mathcal {F}}_{PC}$ the subspace of BV Palatini–Cartan fields defined by the following equations, which we call *PC structural constraints*:

73a $$\begin{aligned}&{\epsilon }_n {\widetilde{e}}{}^{(N-4)} d_{{\widetilde{\omega }}} {\widetilde{e}}- {\epsilon }_n {\widetilde{e}}{}^{(N-4)} {W}_{{\widetilde{e}}{}^{N-3}}^{-1}({\mathfrak {W}}^\dag ) d {\widetilde{\xi }}{}^n \nonumber \\&\quad +\, ( [{\widetilde{c}}, \epsilon _n]+ L_{{\widetilde{\xi }}{}}^{{\widetilde{\omega }}}(\epsilon _n)-d_{{\widetilde{\omega }}_n} \epsilon _n]{\widetilde{\xi }}^{n})^{\{a\}}({\widetilde{\omega }}_a{}^{\!\!\!\dag } -{{\widetilde{c}}}_{an}{}^{\!\!\!\dag }{{\widetilde{\xi }}}{}^n) \in \mathrm {Im}({W}_{{\widetilde{e}}{}^{N-3}}^{1,1}) \end{aligned}$$

73b $$\begin{aligned}&{\mathfrak {W}}^\dag \in \mathrm {Im}({W}_{{\widetilde{e}}{}^{N-3}}^{1,1}) \end{aligned}$$

and by the condition that the metric $g_{\text {hor}}:={\widetilde{e}}^*\eta$ is nowhere degenerate.^25^

#### Remark 53

The PC structural constraints (73b) and (73a) are invariant under the action of $Q_{PC}$. Thus they define a BV theory

74 $$\begin{aligned} {\mathfrak {F}}_{PC}^{\text {res}}:=\left( {\mathcal {F}}_{PC}^{\text {res}},\varpi _{PC}^{\text {res}}=\iota _R^*\varpi _{PC},S_{PC}^{\text {res}}=\iota _R^*S_{PC},Q_{PC}^{\text {res}}\right) \end{aligned}$$

where $Q_{PC}^{\text {res}}$ is the restriction of $Q_{PC}$ to ${\mathcal {F}}_{PC}^{\text {res}}$. We will call this theory the *restricted BV Palatini–Cartan theory*. The direct proof of the invariance of the constraints is lengthy and involved, yet we get this result for free as a corollary of the following theorem, which also specifies the relations between the three BV theories ${\mathfrak {F}}_{PC}^{\text {res}}$, ${\mathfrak {F}}_{PC}$ and ${\mathfrak {F}}_{PC^\star }^{\textsf {\tiny AKSZ}}$.

#### Theorem 54

Upon choosing the same section $\epsilon _n\in \Gamma (M,{\mathcal {V}})$ and the same signature for $g_{\text {hor}}$ in the three theories, the following diagram commutes75Moreover, ${\underline{\varphi }}$ is a symplectomorphism and we have

76 $$\begin{aligned} \varpi ^{\textsf {\tiny AKSZ}}=({\underline{\varphi }}\circ \iota _R)^*\varpi _{PC}; \quad S^{\textsf {\tiny AKSZ}}_{PC}= ({\underline{\varphi }}\circ \iota _R)^* S_{PC}, \end{aligned}$$

so that ${\underline{\varphi }}$ and $\iota$ induce a strong BV equivalence and a BV inclusion, respectively:

$$\begin{aligned} {\mathfrak {F}}^{\textsf {\tiny AKSZ}}_{PC_\star } \xrightarrow {{\underline{\varphi }}} {\mathfrak {F}}_{PC}^{\text {res}} \quad {\mathfrak {F}}^{\textsf {\tiny AKSZ}}_{PC_\star } \xrightarrow {\varphi } {\mathfrak {F}}_{PC}. \end{aligned}$$

#### Remark 55

(Proof Strategy) In order to prove this, we will first show that there is an injective map $\varphi {:}\,{\mathcal {F}}_{PC^\star }^{\textsf {\tiny AKSZ}}\rightarrow {\mathcal {F}}_{PC}$ such that $\varphi ^*\varpi _{PC} = \varpi _{PC}^{\textsf {\tiny AKSZ}}$ and $\varphi ^*S_{PC} = S_{PC}^{\textsf {\tiny AKSZ}}$. Note that, as a symplectomorphism, $\varphi$ is then an immersion. Then we will show that ${\mathcal {F}}_{PC}^{\text {res}}$ is the image of this map, so that the PC structural constraints (73b) and (73a) are satisfied if and only if the AKSZ structural constraints (67b) and (67a) are. The fact that ${\underline{\varphi }}$ is a symplectomorphism preserving the action also proves indirectly that ${\mathfrak {F}}_{PC}^{\text {res}}$ is a BV theory.

#### Proof

Denoting by $\{{\mathbf {e}},\varvec{\omega },{\mathbf {c}},\varvec{\xi }\}$ the fields in ${\mathcal {F}}_{PC}$ (their antifields with a dagger), and following the notation of Eq. (65) for the variables in ${\mathcal {F}}^{\textsf {\tiny AKSZ}}_{PC_\star }$, we define the map $\varphi {:}\,{\mathcal {F}}^{\textsf {\tiny AKSZ}}_{PC_\star }\rightarrow {\mathcal {F}}_{PC}$ in terms of the splitting (70) (with $\varphi ^*$ implicit on the right hand sides):

77a $$\begin{aligned} \varphi ^*{\mathbf {e}}&= {\widetilde{e}}+ {\underline{{\widetilde{e}}}}_n \varphi ^*\varvec{\omega }= {\widetilde{\omega }}+ {\underline{{\widetilde{\omega }}}}_n \varphi ^*{\mathbf {e}}^{\dag }= {\widetilde{e}}{}^{\dag } + {\underline{{\widetilde{e}}}}_n{}^{\!\!\!\dag } \end{aligned}$$

77b $$\begin{aligned} \varphi ^*\varvec{\omega }^{\dag }&= {\widetilde{\omega }}{}^{\dag } + {\underline{{\widetilde{\omega }}}}_n{}^{\!\!\!\dag } \varphi ^*{\mathbf {c}}= {\widetilde{c}}\varphi ^*{\mathbf {c}}^{\dag } = {\underline{{\widetilde{c}}}}_n{}^{\!\!\!\dag } \end{aligned}$$

77c $$\begin{aligned} \varphi ^*\varvec{\xi }&= {\widetilde{\xi }}+ {\underline{{\widetilde{\xi }}}}{}^n \varphi ^*\varvec{\xi }^{\dag }={\underline{{\widetilde{\xi }}}}{}^{\dag }+ {\underline{{\widetilde{\xi }}}}_n{}^{\!\!\!\dag } \end{aligned}$$

where, using again the underlined notation to signify that the quantity is contains $dx^n$ or $\partial _n$, and $a\in \{1,2,\dots , N-1\}$:

78a $$\begin{aligned} {\widetilde{e}}= e + \lambda \mu ^{-1} f^{\dag }&{\underline{{\widetilde{e}}}}_n = \epsilon _n {\underline{\mu }}+\iota _{{\underline{z}}} e + \lambda \mu ^{-1}z^a {\underline{f}}^{\dag }_a \end{aligned}$$

78b $$\begin{aligned} {\widetilde{\omega }}= \omega - \lambda \mu ^{-1} u^{\dag }&{\underline{{\widetilde{\omega }}}}_n = {\underline{w}} - \iota _\xi {\underline{u}}^\dag - \lambda \mu ^{-1}z^a {\underline{u}}^{\dag }_a \end{aligned}$$

78c $$\begin{aligned} {\widetilde{e}}{}^\dag = e^\dag -\lambda \mu ^{-1} y_n^\dag&{\underline{{\widetilde{e}}}}_n{}^{\!\!\!\dag } = e^{N-3} {\underline{u}}^\dag + \iota _{{\underline{z}}} e^\dag -\lambda \mu ^{-1}z^a {\underline{y}}_a^\dag + (N-3)e^{N-4}\lambda \mu ^{-1}f^{\dag }{\underline{u}}^\dag \end{aligned}$$

78d $$\begin{aligned} {\widetilde{\omega }}{}^\dag = k^\dag&{\underline{{\widetilde{\omega }}}}_n{}^{\!\!\!\dag }= e^{N-3} {\underline{f}}^\dag + \iota _{{\underline{z}}} k^\dag + \iota _\xi {\underline{c}}^\dag \end{aligned}$$

78e $$\begin{aligned} {\widetilde{c}}= c - \lambda \mu ^{-1} \iota _{\xi }u^{\dag }&{\underline{{\widetilde{c}}}}_n{}^{\!\!\!\dag }= {\underline{c}}^\dag \end{aligned}$$

78f $$\begin{aligned} {\widetilde{\xi }}{}^{a}= \xi ^a + \lambda \mu ^{-1}z^a&{\underline{{\widetilde{\xi }}}}{}^{\dag }= e {\underline{y}}^\dag + {\underline{f}}^\dag e^\dag - {\underline{u}}^\dag k^\dag + {\underline{c}}^\dag \lambda \mu ^{-1} u^{\dag } \end{aligned}$$

78g $$\begin{aligned} {\underline{{\widetilde{\xi }}}}{}^{n}= {\widetilde{\xi }}{}^n\partial _n= \lambda \mu ^{-1} \partial _n&{\underline{{\widetilde{\xi }}}}_n{}^{\!\!\!\dag }= e_n {\underline{y}}^\dag + e^{N-3} f^\dag {\underline{u}}^\dag + f^\dag \iota _{{\underline{z}}} e^\dag +u^\dag \iota _{{\underline{z}}} k^\dag + c^\dag \lambda \mu ^{-1}z^a {\underline{u}}^{\dag }_a \end{aligned}$$

The explicit, long but straightforward calculation needed to prove that $\varphi$ is an inclusion of symplectic manifolds preserving the action functionals, i.e. such that $\varpi _{PC}^{\textsf {\tiny AKSZ}}=\varphi ^*\varpi _{PC}$ and $S_{PC}^{\textsf {\tiny AKSZ}}=\varphi ^*S_{PC}$, is given in “Appendix A”.

We then need to prove that $\mathrm {Im}(\varphi ) = {\mathcal {F}}_{PC}^{\text {res}}$. In other words we want to prove that the map defined in (78) will map a solution of the constraints (67b) and (67a) into a solution of (73b) and (73a). Applying (78) to the definition of ${\mathfrak {W}}^\dag$ as given in Eq. (72) we get:

$$\begin{aligned} {\mathfrak {W}}^\dag&= {\underline{{\widetilde{\omega }}}}^{\dag }_n - {\widetilde{\omega }}_a{}^{\!\!\!\dag } {{\widetilde{e}}}_n^{\{a\}}dx^n- \iota _{{\widetilde{\xi }}}{\underline{{\widetilde{c}}}}_n{}^{\!\!\!\dag }+{{\widetilde{c}}}_{an}{}^{\!\!\!\dag }{{\widetilde{\xi }}}{}^n{{\widetilde{e}}}_n^{\{a\}}dx^n \\&= e^{N-3} {\underline{f}}^\dag + \iota _{{\underline{z}}} k^\dag + \iota _\xi {\underline{c}}^\dag - k_a^\dag \underline{z_a} - \iota _{\xi }{\underline{c}}^\dag - {\underline{c}}_a^\dag \lambda \mu ^{-1}z^a + {c}_a^\dag \lambda \mu ^{-1}\underline{z_a} \\&= e^{N-3} {\underline{f}}^\dag , \end{aligned}$$

which is (73b). On the other hand, constraint (73a) is satisfied if (67b) and (67a) are, as it can be seen by direct inspection: using (78) we get^26^

$$\begin{aligned}&{\epsilon }_n {\widetilde{e}}{}^{(N-4)} d_{{\widetilde{\omega }}} {\widetilde{e}}- {\epsilon }_n {\widetilde{e}}{}^{(N-4)} {W}_{{\widetilde{e}}{}^{N-3}}^{-1}({\mathfrak {W}}^\dag ) d {\widetilde{\xi }}{}^n \\&\qquad +\, ( [{\widetilde{c}}, \epsilon _n]+ L_{{\widetilde{\xi }}{}}^{{\widetilde{\omega }}}(\epsilon _n)-d_{{\widetilde{\omega }}_n} \epsilon _n]{\widetilde{\xi }}^{n})^{\{a\}}({\widetilde{\omega }}_a{}^{\!\!\!\dag } -{{\widetilde{c}}}_{an}{}^{\!\!\!\dag }{{\widetilde{\xi }}}{}^n)\\&\quad = {\epsilon }_n e^{N-4} d_{\omega } e + (N-4){\epsilon }_n e^{N-5} \lambda \mu ^{-1}f^\dag d_{\omega } e + {\epsilon }_n e^{N-4} [\lambda \mu ^{-1}u^\dag , e] \\&\qquad +\, \epsilon _n e^{N-4} d_{\omega } (\lambda \mu ^{-1}f^\dag )+ (N-4)\epsilon _n e^{N-5} \lambda \mu ^{-1}f^\dag d_{\omega } (\lambda \mu ^{-1}f^\dag ) \\&\qquad +\, \, \epsilon _n f^\dag e^{N-4} d_{\omega } (\lambda \mu ^{-1})+ (N-4) \epsilon _n f^\dag e^{N-5} \lambda \mu ^{-1}f^\dag d_{\omega } (\lambda \mu ^{-1})\\&\qquad -\left( [c, \epsilon _n]-[c, \epsilon _n]^{(b)}\lambda \mu ^{-1}f_b^\dag +[\lambda \mu ^{-1}\iota _\xi u^\dag , \epsilon _n]\right) ^{(a)}(k_a^\dag + c_a^\dag \lambda \mu ^{-1})\\&\qquad +\, \left( L_{\xi }^{\omega }(\epsilon _n)-L_{\xi }^{\omega }(\epsilon _n)^{(b)}\lambda \mu ^{-1}f_b^\dag +[\lambda \mu ^{-1}\iota _\xi u^\dag , \epsilon _n]\right) ^{(a)}(k_a^\dag + c_a^\dag \lambda \mu ^{-1})\\&\qquad -[w-\iota _\xi u^\dag , \epsilon _n]^{(a)}k_a^\dag \lambda \mu ^{-1}\\&\quad = \epsilon _n e^{N-4} d_{\omega } e + \left( L_{\xi }^{\omega }(\epsilon _n) -[c, \epsilon _n]\right) ^{(a)} k^\dag _a \\&\qquad +\, \lambda \mu ^{-1}\Big (\epsilon _n \left\{ (N-4)f^\dag e^{N-5} d_\omega e + e^{N-4} d_{\omega } f^\dag + e^{N-4} [u^\dag , e]\right\} \\&\qquad +\, \left( \iota _z d_{\omega }\epsilon _n-[w-\iota _\xi u^\dag , \epsilon _n]\right) ^{(a)} k^\dag _a\\&\qquad +\, \left( L_{\xi }^{\omega }(\epsilon _n) -[c, \epsilon _n]\right) ^{(a)} c^\dag _a + \left( \left( L_{\xi }^{\omega }(\epsilon _n) -[c, \epsilon _n]\right) ^{(b)}f^\dag _b\right) ^{(a)}k^\dag _a\Big ) = (\spadesuit ). \end{aligned}$$

Using now the AKSZ constraints (67b) and (67a) we obtain

$$\begin{aligned} (\spadesuit )&= e^{N-3} \sigma + \lambda \mu ^{-1} (f^\dag e^{N-4} \sigma + e^{N-3} B)\\&={\widetilde{e}}^{N-3} ( \sigma + \lambda \mu ^{-1} B). \end{aligned}$$

Comparing the first and the last line of this computation we get the desired constraint (73a). Hence $\varphi$ defines a diffeomorphism ${\underline{\varphi }}{:}\,{\mathcal {F}}^{\textsf {\tiny AKSZ}}_{PC_\star } \rightarrow {\mathcal {F}}_{PC}^{\text {res}}$. Indeed, the inverse of this map is readily found, as follows.

It is easy to find $k^\dag ={\widetilde{\omega }}{}^\dag$, ${\underline{c}}^\dag ={\underline{{\widetilde{c}}}}_n{}^{\!\!\!\dag }$, and ${\widetilde{\xi }}{}^n=\lambda \mu ^{-1}$. Then we can write $e = {\widetilde{e}}- {{\widetilde{\xi }}}{}^{n}f^\dag$, so that ${{\widetilde{e}}}_n = \epsilon _n\mu + \iota _z {\widetilde{e}}$, and taking $\{{\widetilde{e}}_a,\epsilon _n\}$ as a basis, we have $z^a = {\widetilde{e}}_n{}^{a}$ and $\mu = {\widetilde{e}}_n{}^n$, which also implies $\lambda = {\widetilde{e}}_n{}^n {\widetilde{\xi }}{}^n$ and $\xi ^a = {\widetilde{\xi }}{}^a - {\widetilde{e}}_n{}^a{\widetilde{\xi }}{}^n$.

We now turn to Eq. (78d) which can be rewritten as

$$\begin{aligned} e^{N-3} {\underline{f}}^\dag = {\underline{{\widetilde{\omega }}}}_n{}^{\!\!\!\dag } - \iota _{{\underline{z}}}k^\dag - \iota _\xi {\underline{c}}^\dag \end{aligned}$$

Let us denote the known piece by $\underline{{\tilde{A}}}:=- \iota _{{\underline{z}}}k^\dag - \iota _\xi {\underline{c}}^\dag$, so that we have

$$\begin{aligned} {\left\{ \begin{array}{ll} e = {\widetilde{e}}- {\widetilde{\xi }}{}^n f^\dag \\ e^{N-3} {\underline{f}}^\dag = {\underline{{\widetilde{\omega }}}}_n{}^{\!\!\!\dag } + \underline{{\tilde{A}}} \end{array}\right. } \Longrightarrow {\widetilde{e}}{}^{N-3} {\underline{f}}^\dag = {\underline{{\widetilde{\omega }}}}_n{}^{\!\!\!\dag } + \underline{{\tilde{A}}} \end{aligned}$$

where we used that $f^\dag f^\dag = 0$. We see here that this equation can be solved only when

$$\begin{aligned} {\underline{{\widetilde{\omega }}}}_n{}^{\!\!\!\dag } - \iota _{{\underline{z}}}{\widetilde{\omega }}{}^\dag - \iota _\xi {\underline{{\widetilde{c}}}}{}^\dag \in \mathrm {Im}({W}^{1,1}_{{\widetilde{e}}{}^{N-3}}). \end{aligned}$$

From the equations

$$\begin{aligned} {\widetilde{e}}^{\dag } = e^\dag - {\widetilde{\xi }}{}^n y^\dag , \quad {\underline{{\widetilde{e}}}}_n{}^{\!\!\!\dag }=e^{N-3}{\underline{u}}^\dag + \iota _{{\underline{z}}}e^\dag - \lambda \mu ^{-1}z^a{\underline{y}}_a^\dag + (N-3)e^{N-4}\lambda \mu ^{-1}f^{\dag }{\underline{u}}^\dag , \end{aligned}$$

using again $e = {\widetilde{e}}- {\widetilde{\xi }}{}^n f^\dag$, we get

$$\begin{aligned} {\widetilde{e}}{}^{N-3}{\underline{u}}^\dag = {\underline{{\widetilde{e}}}}_n{}^{\!\!\!\dag } - \iota _{{\underline{z}}}{\widetilde{e}}{}^\dag . \end{aligned}$$

Since ${\underline{{\widetilde{e}}}}_n{}^{\!\!\!\dag } - \iota _{{\underline{z}}}{\widetilde{e}}{}^\dag \in \Omega ^{(N-2,N-1)}$, on which the map ${W}_{{\widetilde{e}}{}^{N-3}}$ is surjective, we conclude that, up to components $p({\underline{u}}^\dag )$ in the kernel of ${W}_{{\widetilde{e}}{}^{N-3}}$, we can find

$$\begin{aligned} u^\dag = {W}_{{\widetilde{e}}{}^{N-3}}^{-1}({\underline{{\widetilde{e}}}}_n{}^{\!\!\!\dag } - \iota _{{\underline{z}}}{\widetilde{e}}{}^\dag ) + p{\underline{u}}^\dag \end{aligned}$$

However, we know that $u^\dag$ must satisfy the constraint (67a), which (impliclty but uniquely) fixes $p{\underline{u}}^\dag$ as a function of ${\widetilde{e}}, {\widetilde{\omega }}, f^\dag$. We can use this directly to solve

$$\begin{aligned} \omega = {\widetilde{\omega }}+ {\widetilde{\xi }}{}^n u^\dag = {\widetilde{\omega }}+ {\widetilde{\xi }}{}^n\left( {W}_{{\widetilde{e}}{}^{N-3}}^{-1}({\underline{{\widetilde{e}}}}_n{}^{\!\!\!\dag } - \iota _{{\underline{z}}}{\widetilde{e}}{}^\dag ) + p{\underline{u}}^\dag \right) \end{aligned}$$

Analogously we can find ${\underline{w}}$ and *c* as follows

$$\begin{aligned} {\underline{w}}= & {} {\underline{{\widetilde{\omega }}}}_n + \iota _{{\widetilde{\xi }}}\left( {W}_{{\widetilde{e}}{}^{N-3}}^{-1}({\underline{{\widetilde{e}}}}_n{}^{\!\!\!\dag } - \iota _{{\underline{z}}}{\widetilde{e}}{}^\dag ) + p{\underline{u}}^\dag \right) , \\ c= & {} {\widetilde{c}}+{\widetilde{\xi }}{}^n\iota _{{\widetilde{\xi }}}\left( {W}_{{\widetilde{e}}{}^{N-3}}^{-1}({{\widetilde{e}}}_n{}^{\!\!\!\dag } - \iota _{{\underline{z}}}{\widetilde{e}}{}^\dag ) + p{u}^\dag \right) . \end{aligned}$$

Finally, we can conclude the calculation with $y^\dag$ and $e^\dag$ by inverting (78c), (78f) and (78g): it is useful to notice that it is possible to invert an equation of the form $e^\dag (1+ \lambda X)=Y$ for some *X*, *Y* as $e^\dag =Y(1-\lambda X)$. However, we will not write down in full these last equations as we will not need them in what follows.

The BV theory ${\mathfrak {F}}^{\textsf {\tiny AKSZ}}_{PC_\star }$ is obviously strongly equivalent to its image under the symplectomorphism $\varphi$, which is ${\mathfrak {F}}_{PC}^{\text {res}}$. Furthermore, since up to boundary terms $Q_{PC}$ is the Hamiltonian vector field of $S_{PC}$, and the same holds for $Q^{\textsf {\tiny AKSZ}}_{PC}$ and $S_{PC}^{\textsf {\tiny AKSZ}}$, we have that in the interior $M^\circ = M \backslash \partial M$ the compatibility $\varphi ^* Q_{PC} = Q_{PC}^{\textsf {\tiny AKSZ}} \varphi ^*$ is a consequence of $\varphi ^*\varpi _{PC}=\varpi ^{\textsf {\tiny AKSZ}}_{PC}$ and $\varphi ^*S_{PC}=S^{\textsf {\tiny AKSZ}}_{PC}$. However, this is a local condition that then extends to the whole of $(M,\partial M)$ and $\varphi$ is a BV inclusion. $\square$

#### Remark 56

The defining condition $\mu \ne 0$ and ${g}_{\text {hor}}={\widetilde{e}}^*\eta$ nondegenerate given in Definition 49, used in Theorem 54, are necessary in order to make ${\mathbf {e}}$ non degenerate in the bulk, to build the symplectomorphism (78) (since $\epsilon _n^{[n]} = \mu ^{-1}$).

#### Remark 57

The number of free components of $$\iota _R^*{\widetilde{\omega }}$$ is $$\frac{3 N(N-1)}{2}$$, since $$\omega $$ and *w* have respectively $$N(N-1)$$ and $$\frac{N(N-1)}{2}$$ free components. The $$\frac{N(N-1)(N-3)}{2}$$ missing components are those fixed by the condition in Eq. (73a). Correspondingly, also $$\iota _R^*{\widetilde{\omega }}{}^\dag $$ has $$\frac{3 N(N-1)}{2}$$ independent components: $$\frac{N(N-1)}{2}$$ coming from $$k^\dag $$ and $$ N(N-1)$$ from $$f^\dag $$.

### An interpretation of the restricted theory

We now want to shed some light on the interpretation of the restricted theory ${\mathfrak {F}}^{\text {res}}_{PC}$ defined in Theorem 54.

Recall that among the Euler–Lagrange equations of the classical PC theory we have^27^${\mathbf {e}}^{N-3}d_{\varvec{\omega }} {\mathbf {e}}=0$, which, thanks to the nondegeneracy of ${\mathbf {e}}$, is equivalent to $d_{\varvec{\omega }} {\mathbf {e}}=0$, i.e., the torsion-free condition for $\varvec{\omega }$. Imposing this condition forces $\varvec{\omega }$ to correspond to the Levi-Civita connection for the metric $g_{\mu \nu }=\eta ({\mathbf {e}}_\mu ,{\mathbf {e}}_\nu )$, which is used to recover the Einstein–Hilbert formulation of the theory. Note that this yields only a classical equivalence of the two theories, as the fluctuations might violate the condition $d_{\varvec{\omega }} {\mathbf {e}}=0$ at the quantum level. Only by forcing this condition on the space of fields (i.e., by freezing the fluctuations that might violate it) may one recover the quantum Einstein–Hilbert theory.

However, one can consider a whole family of theories between PC and EH where only some part of the condition $d_{\varvec{\omega }} {\mathbf {e}}=0$ is imposed on the fields, looking for a compromise.^28^ that retains the good feature of PC of dealing with differential forms but yields a compatible boundary BFV theory as in EH [CS16].

In particular, working on a cylinder $I\times \Sigma$, we may use the decomposition ${\mathbf {e}}= {\underline{{\widetilde{e}}}}_n+ {\widetilde{e}}$, $\varvec{\omega }= {\underline{{\widetilde{\omega }}}}_n + {\widetilde{\omega }}$. By choosing once and for all a nonzero section $\epsilon _n\in \Gamma (M,{\mathcal {V}})$ and requiring the components of ${\widetilde{e}}$ to span a transversal hyperplane in ${\mathcal {V}}$ at each point, we may expand ${\underline{{\widetilde{e}}}}_n$ in the basis $(\epsilon _n,{\widetilde{e}})$; moreover, we require ${\tilde{e}}$ to define a nondegenerate metric $\eta ({\widetilde{e}},{\widetilde{e}})$ at each point.^29^ Observe that the splitting of fields ${\mathbf {e}},\varvec{\omega }$ induced by the cylinder structure also allows the definition of the maps $W^{i,j}_{{\widetilde{e}}{}^{N-3}}$ given in Eq. (71).

With these notations we may impose the constraint

79 $$\begin{aligned} \epsilon _n{\widetilde{e}}^{N-3}d_{{\widetilde{\omega }}}{\widetilde{e}}\in \mathrm {Im}({W}_{{\widetilde{e}}{}^{N-3}}^{1,1}), \end{aligned}$$

which implements only some of the conditions in $d_{\varvec{\omega }} {\mathbf {e}}=0$.

Another interpretation of the constraints goes through considering a reduction of the fields instead of a restriction. Indeed we can also think of Eq. (79) as a classical constraint that freezes certain components of the connection. We need the following

#### Definition 58

We define the space of reduced connections on a cylinder to be the quotient

80 $$\begin{aligned} {\mathcal {A}}^{\mathrm {red}}(\Sigma \times I):={\mathcal {A}}(\Sigma \times I)/\mathrm {ker}({W}_{{\widetilde{e}}{}^{N-3}}^{1,2}), \end{aligned}$$

and denote by $F_{PC}^{\text {res}}$ the fiber bundle

81 $$\begin{aligned} F_{PC}^{\text {res}} \longrightarrow \Omega ^1_{nd}(\Sigma \times I,{\mathcal {V}}) \end{aligned}$$

with typical fiber ${\mathcal {A}}^{\mathrm {red}}(\Sigma \times I)$ obtained by reducing the fibers of the trivial bundle

$$\begin{aligned} {\mathcal {A}}(\Sigma \times I)\times \Omega ^1_{nd}(\Sigma \times I,{\mathcal {V}}) \longrightarrow \Omega ^1_{nd}(\Sigma \times I,{\mathcal {V}}) \end{aligned}$$

by $$\mathrm {ker}(W_{{\widetilde{e}}{}^{N-3}}^{1,2})$$.

#### Proposition 59

Consider the splitting ${\mathbf {e}}={\widetilde{e}}+ {\underline{{\widetilde{e}}}}_n$, with ${g}_{\text {hor}}:={\widetilde{e}}{}^*\eta$ nondegenerate. Then for every $([\omega ],{\widetilde{e}},{\underline{{\widetilde{e}}}}_n)$ there exists a unique $\omega \in {\mathcal {A}}(\Sigma \times I)$ such that

82 $$\begin{aligned} (N-3)\epsilon _n \wedge {\widetilde{e}}{}^{N-4} \wedge d_{\omega } {\widetilde{e}}\in \mathrm {Im}(W_{{\widetilde{e}}{}^{N-3}}^{1,1}), \end{aligned}$$

which induces a section of the fibration:

83 $$\begin{aligned} {\mathcal {A}}(\Sigma \times I)\times \Omega ^1_{nd}(\Sigma \times I,{\mathcal {V}}) \longrightarrow F^{\text {res}}_{PC}, \end{aligned}$$

#### Proof

This is a straightforward adaptation of [CCS20, Theorem 17], which holds at every point in *I*. $\square$

Hence, imposing only some part of the equation $d_{\varvec{\omega }}{\mathbf {e}}=0$ produces an intermediate theory, that in view of Proposition 59 can be alternatively thought of as Palatini–Cartan theory for a tetrad and a *reduced* connection. However, in both interpretations, fixing a condition only on the classical fields does not produce a symplectic submanifold of the space of BV fields.

If we want to consistently restrict the BV theory of Definition 50 we first have to impose some condition on the antifields as well, in order to ensure that we have a nondegenerate BV form. One can show that (79) actually fixes the components of ${\widetilde{\omega }}$ in the kernel of ${W}^{1,2}_{{\widetilde{e}}{}^{N-3}}$. As a consequence, we can get a symplectic submanifold if, in addition to (79), we impose^30^

84 $$\begin{aligned} {{\widetilde{\omega }}}^{\dag }_n\in \mathrm {Im}({W}_{{\widetilde{e}}{}^{N-3}}^{1,1}). \end{aligned}$$

The problem, though, is that (79) and (84) do not define a *Q*-submanifold, which is needed to have a BV theory. However, one can easily check that condition (79) is compatible with gauge transformations and diffeomorphisms upon using the Euler–Lagrange equations. This implies that it should be possible to correct (79)—and concurrently (84) because we want to preserve the condition that we get a symplectic submanifold—so as to obtain a *Q*-submanifold. The explicit solution to this problem is actually given by (73b) and (73a).

#### Remark 60

Observe that this solution might not be unique, as the choice of a structural constraint we made in Definition 25 was made to render the invariance of (19) more manifest. However, Theorem 54 tells us that a different choice of BFV structural constraint will provide a different extension of the constraint (82) in Palatini–Cartan theory.

### Three dimensional case

When $N=3$ some simplifications occur. Indeed, in this case the inclusion is actually an identity since there are no additional constraints on the field. Furthermore we know that the theory is strongly BV-equivalent, both in the bulk and on the boundary, to the topological *BF* theory, denoted here by ${\mathfrak {F}}^{\textsf {\tiny AKSZ}}_{BF'}$. Hence we can summarize the results in the following theorem.

#### Corollary 61

The theories ${\mathfrak {F}}^{\textsf {\tiny AKSZ}}_{PC_\star }$ and ${\mathfrak {F}}^{\textsf {\tiny AKSZ}}_{BF'}$ are strongly BV equivalent.

#### Proof

The claim follows directly from Theorem 16 given the results of Theorem 54 and of [CS19a], which proves the strong equivalence (at all codimensions) of non-degenerate BF theory and PC gravity in three dimensions. $\square$

Pictorially we can describe the content of Corollary 61 as follows 
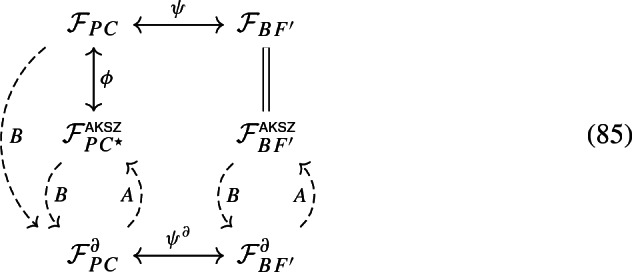
where the arrows *A* represent the AKSZ constructions, the arrows *B* represent the BV–BFV reductions, while $\psi$, $\psi ^{\partial }$ and $\phi$ are the symplectomorphisms mentioned above.

## Appendix A. Lengthy Calculations

We prove here that the transformation (78) is a symplectomorphism between ${\mathcal {F}}^{\textsf {\tiny AKSZ}}$ and ${\mathcal {F}}_R$ that preserves the action. In the computation we will use multiple times the following useful relation:

$$\begin{aligned} \epsilon ^{[a]}_n = - z^a \epsilon ^{[n]}_n, \quad \epsilon ^{[n]}_n = \mu ^{-1}. \end{aligned}$$

We now prove that $\varphi ^*\varpi _{PC} = \varpi _{PC}^{\textsf {\tiny AKSZ}}.$

86 $$\begin{aligned} \varphi ^*\varpi _{PC}&= \varphi ^*\int _M \delta {\mathbf {e}}\delta {\mathbf {e}}{}^{\dag } + \delta \varvec{\omega }\delta \varvec{\omega }{}^{\dag }+ \delta {\mathbf {c}}\delta {\mathbf {c}}{}^{\dag } + \iota _{\delta \varvec{\xi }} \delta \varvec{\xi }{}^{\dag } \nonumber \\&= \int _M \delta {\widetilde{e}}\delta {\underline{{\widetilde{e}}}}_n{}^{\!\!\!\dag } + \delta {\underline{{\widetilde{e}}}}_n \delta {\widetilde{e}}{}^{\dag }+ \delta {\widetilde{\omega }}\delta {\underline{{\widetilde{\omega }}}}_n{}^{\!\!\!\dag }+ \delta {\underline{{\widetilde{\omega }}}}_n \delta {\widetilde{\omega }}{}^{\dag }+ \delta {\widetilde{c}}\delta {\underline{{\widetilde{c}}}}{}^{\dag } + \delta {\widetilde{\xi }}{}^{a} {\underline{{\widetilde{\xi }}}}_a{}^{\!\!\!\dag } + \delta {\widetilde{\xi }}{}^{n} {\underline{{\widetilde{\xi }}}}_n{}^{\!\!\!\dag } \nonumber \\&= \int _M \delta e \delta (e^{N-3} {\underline{u}}^\dag ) + \delta e\delta (\iota _{{\underline{z}}} e^\dag ) - \delta e\delta (\lambda \epsilon _n^{[a]} {\underline{y}}_a^\dag ) +\delta e\delta ((N-3)e^{N-4} \lambda \epsilon _n^{[n]}f^{\dag } {\underline{u}}^\dag ) \nonumber \\&\quad +\, \delta (\lambda \epsilon _n^{[n]} f^{\dag })\delta (e {\underline{u}}^\dag ) + \delta (\lambda \epsilon _n^{[n]} f^{\dag })\delta (\iota _{{\underline{z}}} e^\dag ) -\delta (\lambda \epsilon _n^{[n]} f^{\dag })\delta (\lambda \epsilon _n^{[a]} {\underline{y}}_a^\dag ) \nonumber \\&\quad +\, \delta (\lambda \epsilon _n^{[n]} f^{\dag })\delta (\lambda \epsilon _n^{[n]}f^{\dag }{\underline{u}}^\dag ) + \delta (\iota _{{\underline{z}}} e) \delta e^\dag -\delta (\iota _{{\underline{z}}} e)\delta (\lambda \epsilon _n^{[n]} y^\dag ) + \delta ( \epsilon _n {\underline{\mu }}) \delta e^\dag \nonumber \\&\quad -\delta ( \epsilon _n {\underline{\mu }})\delta (\lambda \epsilon _n^{[n]} y^\dag ) + \delta (\lambda \epsilon _n^{[a]} {\underline{f}}^{\dag }_a)\delta e^\dag -\delta (\lambda \epsilon _n^{[a]} {\underline{f}}^{\dag }_a)\delta (\lambda \epsilon _n^{[n]} y^\dag )\nonumber \\&\quad +\, \delta \omega \delta (e^{N-3} {\underline{f}}^\dag ) + \delta \omega \delta (\iota _{{\underline{z}}} k^\dag ) + \delta \omega \delta (\iota _\xi {\underline{c}}^\dag ) - \delta (\lambda \epsilon _n^{[n]} u^{\dag }) \delta (e^{N-3} {\underline{f}}^\dag ) \nonumber \\&\quad -\, \delta (\lambda \epsilon _n^{[n]} u^{\dag })\delta (\iota _{{\underline{z}}} k^\dag ) - \delta (\lambda \epsilon _n^{[n]} u^{\dag })\delta (\iota _\xi {\underline{c}}^\dag ) + \delta {\underline{w}} \delta k^\dag - \delta (\iota _\xi {\underline{u}}^\dag )\delta k^\dag \nonumber \\&\quad -\, \delta (\lambda \epsilon _n^{[a]} {\underline{u}}^{\dag }_a)\delta k^\dag + \delta c \delta {\underline{c}}^\dag - \delta (\iota _{\xi } \lambda \epsilon _n^{[n]} u^{\dag })\delta {\underline{c}}^\dag + \iota _{\delta \xi } \delta (e {\underline{y}}^\dag ) \nonumber \\&\quad +\, \iota _{\delta \xi }\delta ({\underline{f}}^\dag e^\dag ) - \iota _{\delta \xi }\delta ({\underline{u}}^\dag k^\dag ) + \iota _{ \delta \xi }\delta ({\underline{c}}^\dag \lambda \epsilon _n^{[n]} u^{\dag }) + \delta (\lambda \epsilon ^{[a]}_n) \delta (e_a {\underline{y}}^\dag ) \nonumber \\&\quad +\, \delta (\lambda \epsilon ^{[a]}_n)\delta ({\underline{f}}_a^\dag e^\dag ) + \delta (\lambda \epsilon ^{[a]}_n)\delta ({\underline{u}}_a^\dag k^\dag ) + \delta (\lambda \epsilon ^{[a]}_n) \delta ({\underline{c}}_a^\dag \lambda \epsilon _n^{[n]} u^{\dag })\nonumber \\&\quad +\, \delta (\lambda \epsilon ^{[n]}_n) \delta ({\mathbf {e}}_n {\underline{y}}^\dag ) + \delta (\lambda \epsilon ^{[n]}_n) \delta (e^{N-3} f^\dag {\underline{u}}^\dag ) + \delta (\lambda \epsilon ^{[n]}_n) \delta ( f^\dag \iota _{{\underline{z}}} e^\dag )\nonumber \\&\quad +\, \delta (\lambda \epsilon ^{[n]}_n) \delta (u^\dag \iota _{{\underline{z}}} k^\dag ) + \delta (\lambda \epsilon ^{[n]}_n) \delta (c^\dag \lambda \epsilon _n^{[a]} {\underline{u}}^{\dag }_a) \end{aligned}$$

This expression should be compared with the symplectic form coming from the AKSZ construction:

87 $$\begin{aligned} \varpi ^{\textsf {\tiny AKSZ}}_{PC}&= \int _{I\times \partial M} \delta (e^{N-3} f^\dag ) \delta \omega + e^{N-3} \delta e \delta u^\dag + \delta w \delta k^\dag + \delta c \delta c^\dag + \delta u^\dag \delta (\iota _\xi k^\dag )\nonumber \\&\quad +\, \, \delta \omega \delta (\iota _z k^\dag ) + \delta \omega \delta (\iota _\xi c^\dag ) - \delta \mu \epsilon _n \delta e^\dag - \delta \lambda \epsilon _n \delta y^\dag \nonumber \\&\quad +\, \, \iota _{\delta z}\delta (e e^\dag ) +\iota _{\delta \xi } \delta (f^\dag e^\dag ) +\iota _{\delta \xi } \delta (e y^\dag ). \end{aligned}$$

Almost all the terms in (87) can be directly found in $\varphi ^*\varpi _{PC}$. The remaining terms can be identified using the following relations:

$$\begin{aligned} \delta {\underline{u}}^\dag \delta (\iota _\xi k^\dag )&=- \delta (\iota _\xi {\underline{u}}^\dag ) \delta k^\dag - \iota _{\delta \xi }\delta ( {\underline{u}}^\dag k^\dag );\\ -\delta ( \lambda \epsilon _n )\delta {\underline{y}}^\dag&= \delta (e_a \lambda \epsilon ^{[a]}_n )\delta {\underline{y}}^\dag +\delta ({\widetilde{e}}_n \lambda \epsilon ^{[n]}_n )\delta {\underline{y}}^\dag \\&= \delta ( \lambda \epsilon ^{[a]}_n )\delta (e_a{\underline{y}}^\dag ) - \delta e \delta ( \lambda \epsilon ^{[a]}_n {\underline{y}}_a^\dag ) \\&\quad + \delta ( \lambda \epsilon ^{[n]}_n )\delta ({\widetilde{e}}_n {\underline{y}}^\dag ) - \delta {\underline{{\widetilde{e}}}}_n \delta ( \lambda \epsilon ^{[n]}_n y^\dag );\\ \iota _{\delta {\underline{z}}}\delta (e e^\dag )&= \delta e \delta ( \iota _{{\underline{z}}}e^\dag ) + \delta ( \iota _{{\underline{z}}}e)\delta e^\dag \end{aligned}$$

All the other terms in (86) sum to zero because of the following identities:

$$\begin{aligned}&\delta (\lambda \epsilon _n^{[n]} f^{\dag })\delta (e^{N-3} {\underline{u}}^\dag ) +(N-3)\delta e\delta (e^{N-4}\lambda \epsilon _n^{[n]}f^{\dag } {\underline{u}}^\dag ) \\&\quad -\delta (\lambda \epsilon _n^{[n]} u^{\dag }) \delta (e^{N-3} {\underline{f}}^\dag )+ \delta (\lambda \epsilon ^{[n]}_n) \delta (e^{N-3} f^\dag {\underline{u}}^\dag )=0;\\&\delta (\lambda \epsilon _n^{[n]} f^{\dag })\delta (\iota _{{\underline{z}}} e^\dag )+ \delta (\lambda \epsilon _n^{[a]} {\underline{f}}^{\dag }_a)\delta e^\dag + \delta (\lambda \epsilon ^{[a]}_n)\delta ({\underline{f}}_a^\dag e^\dag )+ \delta (\lambda \epsilon ^{[n]}_n) \delta ( f^\dag \iota _{{\underline{z}}} e^\dag )=0;\\&\quad -\delta (\lambda \epsilon _n^{[n]} u^{\dag })\delta (\iota _{{\underline{z}}} k^\dag )- \delta (\lambda \epsilon _n^{[a]} {\underline{u}}^{\dag }_a)\delta k^\dag + \delta (\lambda \epsilon ^{[a]}_n)\delta ({\underline{u}}_a^\dag k^\dag )+ \delta (\lambda \epsilon ^{[n]}_n) \delta (u^\dag \iota _{{\underline{z}}} k^\dag ) =0;\\&\quad -\delta (\lambda \epsilon _n^{[n]} u^{\dag })\delta (\iota _\xi {\underline{c}}^\dag )-\delta (\iota _{\xi } \lambda \epsilon _n^{[n]} u^{\dag })\delta {\underline{c}}^\dag + \iota _{ \delta \xi }\delta ({\underline{c}}^\dag \lambda \epsilon _n^{[n]} u^{\dag })=0;\\&\delta (\lambda \epsilon ^{[a]}_n) \delta ({\underline{c}}_a^\dag \lambda \epsilon _n^{[n]} u^{\dag })+ \delta (\lambda \epsilon ^{[n]}_n) \delta (c^\dag \lambda \epsilon _n^{[a]} {\underline{u}}^{\dag }_a)=0;\\&\quad -\delta (\lambda \epsilon _n^{[n]} f^{\dag })\delta (\lambda \epsilon _n^{[a]} {\underline{y}}_a^\dag )-\delta (\lambda \epsilon _n^{[a]} {\underline{f}}^{\dag }_a)\delta (\lambda \epsilon _n^{[n]} y^\dag ) =0;\\&\quad (N-3)\delta (\lambda \epsilon _n^{[n]} f^{\dag })\delta (e^{N-4}\lambda \epsilon _n^{[n]}f^{\dag }{\underline{u}}^\dag )=0. \end{aligned}$$

We go on to show that the symplectomorphism $\varphi$ preserves the action i.e. $\varphi ^* S_{PC} = S^{\textsf {\tiny AKSZ}}_{PC}$. We do it by direct inspection^31^:

88 $$\begin{aligned} \varphi ^* S_{PC}&= \varphi ^*\int _{M} \frac{1}{N-2} {\mathbf {e}}{}^{N-2} F_{\varvec{\omega }} + \left( d_{\varvec{\omega }} {\mathbf {c}}- \iota _{\varvec{\xi }} F_{\varvec{\omega }} \right) \varvec{\omega }{}^{\dag } + \left( L_{\varvec{\xi }}^{\varvec{\omega }} {\mathbf {e}}- [{\mathbf {c}}, {\mathbf {e}}]\right) {\mathbf {e}}{}^{\dag }\\&\quad +\, \frac{1}{2}\left( [{\mathbf {c}},{\mathbf {c}}] - \iota _{\varvec{\xi }}\iota _{\varvec{\xi }} F_{\varvec{\omega }} \right) {\mathbf {c}}{}^{\dag } + \frac{1}{2}\iota _{[\varvec{\xi },\varvec{\xi }]} \varvec{\xi }{}^{\dag } \nonumber \\&= \int _{M} {\widetilde{e}}{}^{N-3} {\underline{{\widetilde{e}}}}_n F_{{\widetilde{\omega }}} + \frac{1}{N-2} {\widetilde{e}}{}^{N-2} \underline{F_{{\widetilde{\omega }}_n}}- \left( \iota _{{\widetilde{\xi }}} F_{{\widetilde{\omega }}}+ F_{{\widetilde{\omega }}_n}{\widetilde{\xi }}_{n} - d_{{\widetilde{\omega }}} {\widetilde{c}}\right) {\underline{{\widetilde{\omega }}}}_n{}^{\!\!\!\dag }\nonumber \\&\quad -\, \left( \iota _{{\widetilde{\xi }}{}} \underline{F_{{\widetilde{\omega }}_n}} - \underline{d_{{\widetilde{\omega }}_n}} {\widetilde{c}}\right) {\widetilde{\omega }}{}^{\!\!\!\dag } + \left( L_{{\widetilde{\xi }}}^{{\widetilde{\omega }}}{\widetilde{e}}+ d_{{\widetilde{\omega }}_n} {\widetilde{e}}{\widetilde{\xi }}_{n} + {\widetilde{e}}_n d {\widetilde{\xi }}_{n}- [{\widetilde{c}},{\widetilde{e}}]\right) {\underline{{\widetilde{e}}}}_n{}^{\!\!\!\dag }\nonumber \\&\quad +\, \left( \iota _{{\widetilde{\xi }}}d_{{\widetilde{\omega }}}{\underline{{\widetilde{e}}}}_n + \iota _{{\underline{\partial }}_n {\widetilde{\xi }}}{\widetilde{e}}- \underline{d_{{\widetilde{\omega }}_n}}({\widetilde{e}}_n {\widetilde{\xi }}_{n})- [{\widetilde{c}},{\underline{{\widetilde{e}}}}_n]\right) {\widetilde{e}}{}^\dag \nonumber \\&\quad -\, \left( \frac{1}{2}\iota _{{\widetilde{\xi }}}\iota _{{\widetilde{\xi }}} F_{{\widetilde{\omega }}} + \iota _{{\widetilde{\xi }}{}} F_{{\widetilde{\omega }}_n}{\widetilde{\xi }}_{n} - \frac{1}{2}[{\widetilde{c}},{\widetilde{c}}]\right) {\underline{{\widetilde{c}}}}{}^\dag +\frac{1}{2} \iota _{[{\widetilde{\xi }},{\widetilde{\xi }}]}\underline{{\widetilde{\xi }}{}^\dag }+ \frac{1}{2} \iota _{[{\widetilde{\xi }},{\widetilde{\xi }}]^n}{\underline{{\widetilde{\xi }}}}{}^{\!\!\!\dag } \nonumber \\&= \int _{M} e^{N-3} \epsilon _n {\underline{\mu }} F_{\omega } + e^{N-3} \iota _{{\underline{z}}} e F_{\omega } + e^{N-3} \lambda \epsilon _n^{[a]} {\underline{f}}^{\dag }_a F_{\omega } + (N-3) e^{N-4}\lambda \epsilon _n^{[n]} f^{\dag } \underline{e_n} F_{\omega } \nonumber \\&\quad -\, e^{N-3} \underline{e_n} d_{\omega }(\lambda \epsilon _n^{[n]} u^{\dag }) - e^{N-3}\lambda \epsilon _n^{[a]} {\underline{f}}^{\dag }_a d_{\omega }(\lambda \epsilon _n^{[n]} u^{\dag }) \nonumber \\&\quad -\, (N-3)e^{N-4}\lambda \epsilon _n^{[n]}f^{\dag }\underline{e_n}d_{\omega }(\lambda \epsilon _n^{[n]} u^{\dag }) \nonumber \\&\quad +\, \, \frac{1}{N-2} e^{N-2} \left( {\underline{\partial }}_n \omega - {\underline{\partial }}_n \lambda \epsilon _n^{[n]} u^{\dag } + d_{\omega } {\underline{w}} \right) \nonumber \\&\quad -\, \frac{1}{N-2} e^{N-2} \left( d_{\omega }( \iota _\xi {\underline{u}}^\dag ) + d_{\omega }(\lambda \epsilon _n^{[a]} {\underline{u}}^{\dag }_a) + [\lambda \epsilon _n^{[n]} u^{\dag }, {\underline{w}} - \iota _\xi {\underline{u}}^\dag ] \right) \nonumber \\&\quad +\, \, e^{N-3} \lambda \epsilon _n^{[n]} f^{\dag } \underline{F_{\omega _n}} \nonumber \\&\quad -\, \iota _{\xi }F_{\omega }(e^{N-3} {\underline{f}}^\dag + \iota _{{\underline{z}}} k^\dag + \iota _\xi {\underline{c}}^\dag ) + \iota _{\xi } d_{\omega }(\lambda \epsilon _n^{[n]} u^{\dag })(e^{N-3} {\underline{f}}^\dag + \iota _{{\underline{z}}} k^\dag + \iota _\xi {\underline{c}}^\dag ) \nonumber \\&\quad -\, F_{\omega _a}\lambda \epsilon ^{[a]}_n (e^{N-3} {\underline{f}}^\dag + \iota _{{\underline{z}}} k^\dag + \iota _\xi {\underline{c}}^\dag ) + d_{\omega }(\lambda \epsilon _n^{[n]} u^{\dag })_a \lambda \epsilon ^{[a]}_n (e^{N-3} {\underline{f}}^\dag + \iota _{{\underline{z}}} k^\dag + \iota _\xi {\underline{c}}^\dag ) \nonumber \\&\quad -\, F_{{\widetilde{\omega }}_n} \lambda \epsilon _n^{[n]}e^{N-3} {\underline{f}}^\dag - F_{{\widetilde{\omega }}_n} \lambda \epsilon _n^{[n]} \iota _{{\underline{z}}} k^\dag - F_{{\widetilde{\omega }}_n} \lambda \epsilon _n^{[n]} \iota _\xi {\underline{c}}^\dag \nonumber \\&\quad +\, \, d_{\omega }c (e^{N-3} {\underline{f}}^\dag + \iota _{{\underline{z}}} k^\dag + \iota _\xi {\underline{c}}^\dag ) - [\lambda \epsilon _n^{[n]} u^{\dag },c](e^{N-3} {\underline{f}}^\dag + \iota _{{\underline{z}}} k^\dag + \iota _\xi {\underline{c}}^\dag ) \nonumber \\&\quad -\, d_{\omega }(\iota _{\xi } \lambda \epsilon _n^{[n]} u^{\dag })(e^{N-3} {\underline{f}}^\dag + \iota _{{\underline{z}}} k^\dag + \iota _\xi {\underline{c}}^\dag ) \nonumber \\&\quad -\, \iota _{\xi }{\underline{\partial }}_n \omega k^\dag + \iota _{\xi }{\underline{\partial }}_n ( \lambda \epsilon _n^{[n]} u^{\dag }) k^\dag - \iota _{\xi }d_{\omega } {\underline{w}} k^\dag + \iota _{\xi }d_{\omega }(\iota _\xi {\underline{u}}^\dag ) k^\dag \nonumber \\&\quad +\, \, \iota _{\xi }d_{\omega }(\lambda \epsilon _n^{[a]} {\underline{u}}^{\dag }_a) k^\dag + \iota _{\xi }[\lambda \epsilon _n^{[n]} u^{\dag }, {\underline{w}} - \iota _\xi {\underline{u}}^\dag ] k^\dag - \underline{F_{{\widetilde{\omega }}_{an}}} \lambda \epsilon ^{[a]}_n k^\dag + {\underline{\partial }}_n c k^{\dag } \nonumber \\&\quad -\, {\underline{\partial }}_n (\iota _{\xi } \lambda \epsilon _n^{[n]} u^{\dag }) k^{\dag } + [ {\underline{w}},c]k^{\dag } - [\iota _\xi {\underline{u}}^\dag ,c]k^{\dag } - [\lambda \epsilon _n^{[a]} {\underline{u}}^{\dag }_a,c]k^{\dag } \nonumber \\&\quad +\, \, [ {\underline{w}} - \iota _\xi {\underline{u}}^\dag ,\iota _{\xi } \lambda \epsilon _n^{[n]} u^{\dag }]k^{\dag } \nonumber \\&\quad +\, \, L_{\xi }^{\omega } e e^{N-3} {\underline{u}}^{\dag } + L_{\xi }^{\omega } e \iota _{{\underline{z}}} e^\dag - L_{\xi }^{\omega } e \lambda \epsilon _n^{[a]} {\underline{y}}_a^\dag + (N-3) L_{\xi }^{\omega } e e^{N-4}\lambda \epsilon _n^{[n]}f^{\dag }{\underline{u}}^\dag \nonumber \\&\quad + ((d_{\omega }e)_a \lambda \epsilon _n^{[a]} - d_{\omega }(e_a \lambda \epsilon _n^{[a]})) e^{N-3} {\underline{u}}^{\dag } + ((d_{\omega }e)_a \lambda \epsilon _n^{[a]} - d_{\omega }(e_a \lambda \epsilon _n^{[a]})) \iota _{{\underline{z}}} e^\dag \nonumber \\&\quad +\, d_{\omega }(e_a \lambda \epsilon _n^{[a]})\lambda \epsilon _n^{[a]} {\underline{y}}_a^\dag - (N-3) d_{\omega }(e_a \lambda \epsilon _n^{[a]})e^{N-4}\lambda \epsilon _n^{[n]}f^{\dag }{\underline{u}}^\dag \nonumber \\&\quad +\, \, L_{\xi }^{\omega } (\lambda \epsilon _n^{[n]} f^{\dag }) e^{N-3} {\underline{u}}^{\dag }\nonumber \\&\quad +\, \, L_{\xi }^{\omega } (\lambda \epsilon _n^{[n]} f^{\dag }) \iota _{{\underline{z}}} e^\dag - L_{\xi }^{\omega } (\lambda \epsilon _n^{[n]} f^{\dag }) \lambda \epsilon _n^{[a]} {\underline{y}}_a^\dag + (d_{\omega }(\lambda \epsilon _n^{[n]} f^{\dag }))_a \lambda \epsilon _n^{[a]} e^{N-3} {\underline{u}}^{\dag } \nonumber \\&\quad +\, \, (d_{\omega }(\lambda \epsilon _n^{[n]} f^{\dag }))_a \lambda \epsilon _n^{[a]}\iota _{{\underline{z}}} e^\dag ) - [\iota _{\xi }\lambda \epsilon _n^{[n]} u^{\dag },e] (e^{N-3} {\underline{u}}^{\dag } + \iota _{{\underline{z}}} e^\dag ) + \partial _n e \lambda \epsilon _n^{[n]} e^{N-3} {\underline{u}}^{\dag } \nonumber \\&\quad +\, \, \partial _n e \lambda \epsilon _n^{[n]} \iota _{{\underline{z}}} e^\dag + [w - \iota _{\xi }u^{\dag }, e] \lambda \epsilon _n^{[n]} e^{N-3} {\underline{u}}^{\dag } + [w - \iota _{\xi }u^{\dag }, e] \lambda \epsilon _n^{[n]}\iota _{{\underline{z}}} e^\dag \nonumber \\&\quad +\, \partial _n (\lambda \epsilon _n^{[n]} f^{\dag }) \lambda \epsilon _n^{[n]}(e^{N-3} {\underline{u}}^{\dag } + \iota _{{\underline{z}}} e^\dag ) + \underline{e_n}d(\lambda \epsilon _n^{[n]}) e^{N-3} {\underline{u}}^{\dag } + \underline{e_n}d(\lambda \epsilon _n^{[n]})\iota _{{\underline{z}}} e^\dag \nonumber \\&\quad +\, \, \lambda \epsilon _n^{[a]} f^{\dag }_a d(\lambda \epsilon _n^{[n]}) e^{N-3} {\underline{u}}^{\dag } + \lambda \epsilon _n^{[a]} f^{\dag }_a d(\lambda \epsilon _n^{[n]}) \iota _{{\underline{z}}} e^\dag - \underline{e_n}d(\lambda \epsilon _n^{[n]}) \lambda \epsilon _n^{[a]} {\underline{y}}_a^\dag \nonumber \\&\quad + (N-3) \underline{e_n}d(\lambda \epsilon _n^{[n]})e^{N-4}\lambda \epsilon _n^{[n]}f^{\dag }{\underline{u}}^\dag - [c,e]e^{N-3} {\underline{u}}^{\dag } - [c,e] \iota _{{\underline{z}}} e^\dag + [c,e]\lambda \epsilon _n^{[a]} {\underline{y}}_a^\dag \nonumber \\&\quad -\, (N-3)[c,e]e^{N-4}\lambda \epsilon _n^{[n]}f^{\dag }{\underline{u}}^\dag - [c, \lambda \epsilon _n^{[n]} f^{\dag }] e^{N-3} {\underline{u}}^{\dag } - [c, \lambda \epsilon _n^{[n]} f^{\dag }]\iota _{{\underline{z}}} e^\dag \nonumber \\&\quad +\, [\iota _{\xi } \lambda \epsilon _n^{[n]} u^{\dag },e](e^{N-3} {\underline{u}}^{\dag } + \iota _{{\underline{z}}} e^\dag ) \nonumber \\&\quad +\, \, \iota _{\xi } d_{\omega }( \epsilon _n {\underline{\mu }})e^\dag + \iota _{\xi } d_{\omega }(\iota _{{\underline{z}}} e )e^\dag + \iota _{\xi } d_{\omega }(\lambda \epsilon _n^{[a]} {\underline{f}}^{\dag }_a)e^\dag + d_{\omega _a}( \underline{e_n})\lambda \epsilon _n^{[a]} e^\dag \nonumber \\&\quad +\, \, d_{\omega _a}(\lambda \epsilon _n^{[a]} {\underline{f}}^{\dag }_a)\lambda \epsilon _n^{[a]} e^\dag - \iota _{\xi } [\lambda \epsilon _n^{[n]} u^{\dag }, \underline{e_n}] e^\dag - \iota _{\xi } d_{\omega }( \underline{e_n} )\lambda \epsilon _n^{[n]} y_n^\dag \nonumber \\&\quad -\, \iota _{\xi } d_{\omega }(\lambda \epsilon _n^{[a]} {\underline{f}}^{\dag }_a)\lambda \epsilon _n^{[n]} y_n^\dag - \iota _{{\underline{\partial }}_n \xi } e e^\dag + e_a {\underline{\partial }}_n (\lambda \epsilon _n^{[a]}) e^\dag - \iota _{{\underline{\partial }}_n \xi {} } \lambda \epsilon _n^{[n]} f^{\dag } e^\dag \nonumber \\&\quad +\, \iota _{{\underline{\partial }}_n \xi {} } e \lambda \epsilon _n^{[n]} y_n^\dag - {\underline{\partial }}_n ( {\widetilde{e}}_n \lambda \epsilon _n^{[n]} )e^\dag - [{\underline{w}} - \iota _\xi {\underline{u}}^\dag , {\widetilde{e}}_n \lambda \epsilon _n^{[n]}]e^\dag \nonumber \\&\quad +\, \, {\underline{\partial }}_n ( {\widetilde{e}}_n \lambda \epsilon _n^{[n]} )\lambda \epsilon _n^{[n]} y_n^\dag - [c, \epsilon _n {\underline{\mu }} ]e^\dag - [c,\iota _{{\underline{z}}} e]e^\dag - [c, \lambda \epsilon _n^{[a]} {\underline{f}}^{\dag }_a]e^\dag \nonumber \\&\quad +\, \, [\iota _{\xi } \lambda \epsilon _n^{[n]} u^{\dag }, \underline{e_n} ]e^\dag + [c, \underline{e_n} ]\lambda \epsilon _n^{[n]} y_n^\dag \nonumber \\&\quad -\, \frac{1}{2} \iota _{\xi }\iota _{\xi } F_{\omega } {\underline{c}}^{\dag } + \frac{1}{2} \iota _{\xi }\iota _{\xi }d_{\omega }(\lambda \epsilon _n^{[n]} u^{\dag }){\underline{c}}^{\dag } - \iota _{\xi } F_{\omega _a} \lambda \epsilon _n^{[a]} {\underline{c}}^{\dag } \nonumber \\&\quad +\, \, \iota _{\xi }d_{\omega }(\lambda \epsilon _n^{[n]} u^{\dag })_a \lambda \epsilon _n^{[a]} {\underline{c}}^{\dag } - \iota _{\xi } F_{{\widetilde{\omega }}_n} \lambda \epsilon _n^{[n]} {\underline{c}}^{\dag } + \frac{1}{2}[c,c]{\underline{c}}^{\dag } - [\iota _{\xi } \lambda \epsilon _n^{[n]} u^{\dag },c]{\underline{c}}^{\dag } \nonumber \\&\quad -\, \xi ^b \partial _b \xi ^a e_a {\underline{y}}^\dag - \xi ^b \partial _b (\lambda \epsilon _n^{[a]})e_a {\underline{y}}^\dag - \lambda \epsilon _n^{[b]}\partial _b \xi ^a e_a {\underline{y}}^\dag - \lambda \epsilon _n^{[b]}\partial _b (\lambda \epsilon _n^{[a]}) e_a {\underline{y}}^\dag \nonumber \\&\quad -\, \lambda \epsilon _n^{[n]}\partial _n \xi {a} e_a {\underline{y}}^\dag - \xi ^b \partial _b \xi ^a{\underline{f}}_a^\dag e^\dag - \xi ^b \partial _b (\lambda \epsilon _n^{[a]}){\underline{f}}_a^\dag e^\dag - \lambda \epsilon _n^{[b]}\partial _b \xi ^a {\underline{f}}_a^\dag e^\dag \nonumber \\&\quad -\, \lambda \epsilon _n^{[b]}\partial _b (\lambda \epsilon _n^{[a]}) {\underline{f}}_a^\dag e^\dag - \lambda \epsilon _n^{[n]}\partial _n \xi {a} {\underline{f}}_a^\dag e^\dag - \xi ^b \partial _b \xi ^a {\underline{u}}_a^\dag k^\dag - \xi ^b \partial _b (\lambda \epsilon _n^{[a]}){\underline{u}}_a^\dag k^\dag \nonumber \\&\quad -\, \lambda \epsilon _n^{[b]}\partial _b \xi ^a {\underline{u}}_a^\dag k^\dag + \lambda \epsilon _n^{[b]}\partial _b (\lambda \epsilon _n^{[a]}){\underline{u}}_a^\dag k^\dag - \lambda \epsilon _n^{[n]}\partial _n \xi ^a{\underline{u}}_a^\dag k^\dag \nonumber \\&\quad -\, \lambda \epsilon _n^{[n]}\partial _n (\lambda \epsilon _n^{[a]}){\underline{u}}_a^\dag k^\dag - \xi ^b \partial _b \xi ^a {\underline{c}}_a^\dag \lambda \epsilon _n^{[n]} u^{\dag } - \xi ^b \partial _b (\lambda \epsilon _n^{[a]}){\underline{c}}_a^\dag \lambda \epsilon _n^{[n]} u^{\dag } \nonumber \\&\quad -\, \xi ^a \partial _a (\lambda \epsilon _n^{[n]}) {\widetilde{e}}_n {\underline{y}}^\dag - \lambda \epsilon _n^{[a]}\partial _a (\lambda \epsilon _n^{[n]}){\widetilde{e}}_n {\underline{y}}^\dag - \lambda \epsilon _n^{[n]}\partial _n (\lambda \epsilon _n^{[n]}){\widetilde{e}}_n {\underline{y}}^\dag \nonumber \\&\quad -\, \xi ^a \partial _a (\lambda \epsilon _n^{[n]})f^\dag e^{N-3} {\underline{u}}^\dag - \lambda \epsilon _n^{[a]}\partial _a (\lambda \epsilon _n^{[n]})f^\dag e^{N-3} {\underline{u}}^\dag - \lambda \epsilon _n^{[n]}\partial _n (\lambda \epsilon _n^{[n]})f^\dag e^{N-3} {\underline{u}}^\dag \nonumber \\&\quad -\, \xi ^a \partial _a (\lambda \epsilon _n^{[n]})f^\dag \iota _{{\underline{z}}} e^\dag - \lambda \epsilon _n^{[a]}\partial _a (\lambda \epsilon _n^{[n]}) f^\dag \iota _{{\underline{z}}} e^\dag - \lambda \epsilon _n^{[n]}\partial _n (\lambda \epsilon _n^{[n]})f^\dag \iota _{{\underline{z}}} e^\dag \nonumber \\&\quad + \xi ^a \partial _a (\lambda \epsilon _n^{[n]})u^\dag \iota _{{\underline{z}}} k^\dag + \lambda \epsilon _n^{[a]}\partial _a (\lambda \epsilon _n^{[n]})u^\dag \iota _{{\underline{z}}} k^\dag + \lambda \epsilon _n^{[n]}\partial _n (\lambda \epsilon _n^{[n]})u^\dag \iota _{{\underline{z}}} k^\dag \nonumber \\&\quad -\, \xi ^a \partial _a (\lambda \epsilon _n^{[n]})c^\dag \lambda \epsilon _n^{[a]} {\underline{u}}^{\dag }_a \nonumber \end{aligned}$$

We want to compare this with the AKSZ action:

89 $$\begin{aligned} S^{\textsf {\tiny AKSZ}}_{PC}&=\int _{I\times \partial M} {\underline{w}} e^{N-3} d_{\omega } e +(N-3) c e^{N-4} \underline{f^\dag } d_{\omega } e +c e^{N-3} [\underline{u^\dag }, e] +c e^{N-3} d_{\omega } \underline{f^\dag } \nonumber \\&\quad +\, \iota _{{\underline{z}}} e e^{N-3} F_{\omega } + \iota _{\xi }( e^{N-3} \underline{f^\dag }) F_{\omega } + \iota _{\xi } e e^{N-3} d_{\omega }\underline{u^\dag } + \epsilon _n {\underline{\mu }} e^{N-3} F_{\omega }\nonumber \\&\quad +\, (N-3) \epsilon _n \lambda e^{N-4} \underline{f^\dag } F_{\omega } + \epsilon _n \lambda e^{N-3} d_{\omega }\underline{u^\dag } + [{\underline{w}},c] k^{\dag } +\frac{1}{2} [c,c] \underline{c^{\dag }} \nonumber \\&\quad -\, \iota _{{\underline{z}}} d_{\omega } c k^{\dag } - [\iota _{\xi }\underline{u^\dag },c] k^{\dag } - \iota _{\xi } d_{\omega }{\underline{w}} k^{\dag } - \iota _{\xi } d_{\omega } c \underline{c^{\dag }} + \iota _{{\underline{z}}}\iota _{\xi } F_{\omega }k^{\dag }\nonumber \\&\quad +\, \frac{1}{2} \iota _{\xi }\iota _{\xi } d_{\omega }\underline{u^\dag } k^{\dag } + \frac{1}{2} \iota _{\xi }\iota _{\xi } F_{\omega }\underline{c^{\dag }} -[{\underline{w}}, \epsilon _n \lambda ]e^{\dag } -[c, \epsilon _n {\underline{\mu }}]e^{\dag } -[c, \epsilon _n \lambda ]\underline{y^{\dag }} \nonumber \\&\quad +\, \iota _{{\underline{z}}}d_{\omega } ( \epsilon _n\lambda )e^{\dag } + [\iota _{\xi }\underline{u^\dag }, \epsilon _n\lambda ]e^{\dag } + \iota _{\xi }d_{\omega } ( \epsilon _n{\underline{\mu }})e^{\dag } + \iota _{\xi }d_{\omega } ( \epsilon _n\lambda )\underline{y^{\dag }} + \iota _{[{\underline{z}},\xi ]}e e^{\dag }\nonumber \\&\quad +\, \frac{1}{2}\iota _{[\xi ,\xi ]}\underline{f^\dag } e^{\dag } + \frac{1}{2}\iota _{[\xi ,\xi ]}e \underline{y^{\dag }} + \frac{1}{N-2} e^{N-2} \underline{\partial _n} \omega + c \underline{\partial _n} k^\dag + \underline{\partial _n} \omega \iota _\xi k^\dag \nonumber \\&\quad -\, \iota _{\underline{\partial _n} \xi } e e^\dag + \underline{\partial _n} \lambda \epsilon _n e^\dag . \end{aligned}$$

We proceed as follows: we first check that all terms in (89) can be found in (88), then we show that all other terms in (88) sum to zero.

We can easily recognized many terms identically repeated in both expressions. Some other terms in (89) can be recovered in (88) using Leibniz rule and Cartan calculus.

$$\begin{aligned}&(N-3) c e^{N-4} {\underline{f}}^\dag d_{\omega } e + c ^{N-3} d_{\omega } {\underline{f}}^\dag = d_{\omega } c ( e^{N-3} {\underline{f}}^\dag ); \\&\quad -\,\frac{1}{N-2} e^{N-2} d_{\omega }( \iota _\xi {\underline{u}}^\dag ) +L_{\xi }^{\omega } e e^{N-3} {\underline{u}}^{\dag }= +\iota _{\xi } e e^{N-3} d_{\omega } {\underline{u}}^{\dag }; \\&\quad -\, \frac{1}{2}\iota _{[\xi ,\xi ]}{\underline{u}}^\dag k^\dag = - \iota _{\xi } d_{\omega }\iota _{\xi }{\underline{u}}^\dag k^\dag + \frac{1}{2} \iota _{\xi } \iota _{\xi }d_{\omega } {\underline{u}}^\dag k^\dag ; \\&\quad \quad \iota _{[{\underline{z}},\xi ]}e e^{\dag } = \iota _{\xi } d_{\omega } \iota _{{\underline{z}}} e e^{\dag } + L_{\xi }^{\omega } e \iota _{{\underline{z}}} e^{\dag } \end{aligned}$$

All the other relations involving terms of (88) are based on the expansion

$$\begin{aligned} \epsilon _n = e_a \epsilon _n^{(a)} +e_n \epsilon _n^{(n)}. \end{aligned}$$

It is a long but rather easy computation to show that the remaining terms in (89) sum to zero. This is done by making repeated use of Cartan calculus and Leibniz rule. Notice also that some terms containing expressions of the form $\epsilon _{n}^{[a]}\epsilon _{n}^{[b]} X_{ab}$ vanish by antisymmetry.

## Footnotes

1 There appears to be no uniform consensus on the nomenclature to best attribute and label the theory that will be described in Sects. 2.2 and 4. We discuss our choice “Palatini–Cartan” in [CS19c] (see also references therein). Other choices include the names of Einstein, Weyl, Sciama and Kibble, in various combinations.

2 To the best of our knowledge, the first explicit application of the AKSZ construction to a BFV target to produce a BV structure in one dimension goes back to [GD00].

3 This construction is clarified in Theorem 14, and ${{\,\mathrm{\textit{AKSZ}}\,}}({\mathfrak {F}}^\partial )$ will be denoted ${\mathfrak {F}}^{\textsf {\tiny AKSZ}}(I;{\mathfrak {F}}^\partial )$.

4 This is more appropriately called BV-pushforward or BV fiber integral, see [CMR18, Section 2.2.2].

5 On an open subset of the moduli space of solutions.

6 This association is the natural restriction of fields and normal jets to the boundary, see [CMR14].

7 In the math literature, a map with this compatibility between the symplectic structures and the cohomological vector fields is known as a morphism of dg symplectic manifolds.

8 Classical equivalence is the less restrictive requirement that only their 0-cohomologies be isomorphic.

9 In an optimal scenario, to auxiliary fields one can associate a subcomplex with trivial cohomology. More generally, one has a filtration of the original BV complex, so that the associated spectral sequence converges. However, this is in general quite hard to prove.

10 Observe that this is essentially the strictification requirement of the presymplectic BFV data, see Remark 29.

11 Recall that a function on T[1]N is the same as a differential form on N. Integrating a function on *T*[1]*N* against the canonical Berezinian $\mu _N$ is by definition the same as integrating the corresponding differential form on *N*, which we assume to be oriented.

12 In this paper we will mostly focus on the case where $\Sigma$ is a Riemannian manifold, seen as a spacelike boundary of a cylinder $\Sigma \times {\mathbb {R}}$. Generalisations of this to the timelike case are straightforward. The relevant BFV data can be found in [CS16].

13 This is not really problematic, since its variation reads $\delta \sqrt{{\gamma }}= \frac{1}{2} \sqrt{{\gamma }}{\gamma }^{ab}\delta {\gamma }_{ab} = -\frac{1}{2} \sqrt{{\gamma }}{\gamma }_{ab}\delta {\gamma }^{ab}$ and we can use either formula according to our needs. If we wanted to use the correct notation we should simply replace $\sqrt{{\gamma }}$ with its reciprocal, in formula (13).

14 For simplicity we orient $\Sigma$ and *V*, but the formalism generalizes to nonorientable $\Sigma$ as well, see [CCS20].

15 This BFV structural constraint differs from the classical one of Eq. (17) by a term depending on ghosts and their antifields, so it reduces to the classical structural constraint if we restrict it to the classical space of boundary fields (the body of ${\check{{\mathcal {F}}}}_{PC}(\Sigma )$).

16 This version of the BFV data features a particularly simple action functional, at the price of not expressing the symplectic form in its Darboux form. An alternative can be found in [CCS20].

17 Also note that this term vanishes on the body of ${{\check{{\mathcal {F}}}}}_{PC}$ when imposing the classical constraints, i.e., the expressions paired to the ghosts in the first three terms of (20). The two BFV constructions, by (17) or by (19), describe the characteristic distribution of the coisotropic submanifold determined by the constraints. They extend this distribution in a different way outside of it, but this is irrelevant.

18 Equation (19) can be extended to the space of fields over a cylinder. We consider this point of view in Definition 58.

19 The motivation for this particular choice of notation will be manifest very soon.

20 To be precise, ${\mathsf {W}}_\gamma$ is the inverse of the metric introduced by deWitt, due to our choice of working with inverse metrics $\gamma$.

21 We stress that, as it is, the structure one can extract from the BFV differential $Q^\partial$ is that of a curved $L_\infty$ algebroid, due to the dependency on fields of negative degree. We thank A. Weinstein, C. Blohmann and N. L. Delgado for enlightening discussions on this matter.

22 In this paper we will assume that the manifold *M* has a global product structure $M=\Sigma \times \mathrm {R}$, and the induced metric on $\Sigma$ will be Riemannian, i.e. the leaves $\Sigma _t$ are spacelike submanifolds of *M*. It is straightforward to generalise this to the *timelike* case. The relevant formulas for EH theory in the BV–BFV formalism have been given in [CS16].

23 Strictly speaking this is only true on an open subset of the moduli space of solutions, due to the nondegeneracy condition on $\gamma (t)$ enforced on the whole cylinder by the AKSZ construction, while Definition 53 only requires the nondegeneracy of ${\widetilde{g}}\vert _{\partial M}$.

24 See [MSW19] for the comparison between strict and lax BV–BFV structures.

25 Notice that the condition on $g_{\text {hor}}$ will restrict the moduli space of solutions of the theory to an open subset.

26 Note that $$X^{\{a\}}=X^{(a)}-(X^{(b)} \lambda \mu ^{-1}f_b^\dag )^{(a)}$$.

27 We use boldface letters to denote fields in PC theory.

28 Imposing too few conditions out of $d_\omega e=0$ would not solve the compatibility problem with the boundary. Imposing too many generates other problems (see, e.g., [CS19c, Section 4.3], where the whole of $d_\omega e=0$ is imposed manually).

29 A more physical requirement, as one would like the two boundary components of $I\times \Sigma$ to be space-like Cauchy surfaces, consists in choosing $\epsilon _n$ to be a time-like section and ${\widetilde{e}}$ to define a positive definite metric.

30 This condition requires that the antifield of ${\widetilde{\omega }}$ be in the dual of the complement of the kernel of $W_{{\widetilde{e}}{}^{N-3}}^{1,2}$, which is the image of $W_{{\widetilde{e}}{}^{N-3}}^{1,1}$ because ${\widetilde{\omega }}$ is tangent to the slice $\Sigma \times \{t\}$, and its antifield is of the form ${\widetilde{\omega }}^\dag _n dx^n$, with ${\widetilde{\omega }}^\dag _n\in \Omega ^{N-2}(M,\wedge ^{N-2}{\mathcal {V}})$.
